# Vision and convolutional transformers for Alzheimer’s disease diagnosis: a systematic review of architectures, multimodal fusion and critical gaps

**DOI:** 10.1186/s40708-025-00286-7

**Published:** 2025-12-17

**Authors:** Ibrahem Afifi, Mostafa Elgendy, Mohamed Abdelfatah, Shaker El-Sappagh

**Affiliations:** 1 Information Systems Department, Faculty of Computers and Artificial Intelligence, Benha University, Benha, 13518 Egypt; 2 Computer Science Department, Faculty of Computers and Artificial Intelligence, Benha University, Benha, 13518 Egypt; 3 Faculty of Computer Science and Engineering, Galala University, Suez, 435611 Egypt

**Keywords:** Alzheimer’s disease (AD), Mild cognitive impairment (MCI), Deep learning (DL), Vision transformers (ViTs), Convolutional vision transformers (CViTs), Systematic review, Multimodal fusion, Disease progression prediction, Large vision models (LVMs), Explainable AI (XAI)

## Abstract

Alzheimer’s disease (AD), a significant public health challenge, requires accurate early diagnosis to improve patient outcomes. Vision Transformers (ViTs) and Convolutional Vision Transformers (CViTs) have emerged as powerful Deep Learning architectures for this task. Following PRISMA guidelines, this systematic review analyzes 68 studies selected from 564 publications (2021–2025) across five major databases: Scopus, Web of Science, ScienceDirect, IEEE Xplore, and PubMed. We introduce novel taxonomies to systematically categorize these works by model architecture, data modality, fusion strategy, and diagnostic objective. Our analysis reveals key trends, such as the rise of hybrid CViT frameworks, and critical gaps, including a limited focus on Mild Cognitive Impairment-to-AD progression. Critically, we also assess practical implementation details, revealing widespread challenges in algorithmic reproducibility. The discussion culminates in a forward-looking analysis of Large Vision Models and proposes future directions emphasizing the need for robust multimodal integration, lightweight transformer designs, and Explainable AI to advance AD research and bridge the critical gap between high-performance modeling and clinical applicability.

## Introduction

The aging of the global population presents numerous societal challenges, among which the increasing prevalence of AD stands out as a significant public health concern [1–3]. As a progressive neurodegenerative disorder, AD leads to a debilitating decline in memory and other cognitive functions, severely impacting the quality of life and daily functioning of affected individuals and their families. The impact of AD escalates sharply with age; statistical data highlight that while approximately 3% of individuals aged $$65-74$$ are affected, the incidence rises substantially to about 19% within the $$75-84$$ age bracket, and approaches nearly 47% in those over 84 years old [1, 2, 4]. These figures expose the deep-seated connection between advancing age and the probability of developing AD, a relationship that is now translating into a formidable societal challenge [5]. The burden of the disease is intensifying on a global scale, with current estimates placing over 55 million people worldwide with dementia. Projections indicate this number will dramatically escalate to 139 million by mid-century, with low- and middle-income countries facing the most severe consequences [6, 7]. The financial implications of this trend are equally severe. The global annual expenditure for dementia care, currently estimated at US$1.3 trillion, is on a path to more than double, reaching an estimated US$2.8 trillion by 2030 [6]. The United States provides a stark case study of this trajectory, with projections showing a potential rise in its AD population from 6.5 million to 13.8 million by 2060, should no major preventative or therapeutic advances be made [4]. This escalating epidemiological and economic impact underscores the urgent need to advance our diagnostic toolkit. Consequently, achieving earlier and more accurate disease detection has become a paramount objective, essential for optimizing patient care and enabling the successful development of new therapeutic strategies [8].

Traditional diagnostic approaches for AD typically combine clinical evaluations and neuropsychological testing with insights from neuroimaging and biomarker analysis [9, 10]. Structural Magnetic Resonance Imaging (sMRI) provides information on brain atrophy patterns, while Positron Emission Tomography (PET) can detect metabolic changes and specific molecular pathologies like amyloid-beta and tau deposits [11, 12]. Analysis of Cerebrospinal Fluid (CSF) or blood offers further specificity regarding these core biomarkers [13–15]. However, these conventional methods possess inherent limitations, including inter-observer variability in image interpretation, the need for specialized equipment and expertise which limits accessibility, and the significant challenge of effectively integrating information from these diverse modalities to achieve a comprehensive understanding of an individual’s disease status and trajectory [16, 17].

While initial research into computer-aided AD diagnosis utilized traditional Machine Learning (ML) techniques [13, 14, 18, 19], DL approaches have subsequently demonstrated superior performance [20–23]. Both ML and DL aim to identify salient features or patterns within complex datasets to enable optimal classification or prediction [24–26]. More recently, ViTs, adapted from their success in natural language processing [27], have emerged as a powerful alternative to Convolutional Neural Networks (CNNs) for analyzing medical images [28, 29]. ViTs employ self-attention mechanisms capable of capturing both local and global dependencies within imaging data, making them potentially more effective for identifying the complex neuroimaging patterns associated with AD [28]. Recognizing the high computational demands and data requirements of pure ViTs, hybrid architectures known as CViTs have been developed. These models combine the strengths of CNNs for efficient local feature extraction with the global contextual modeling capabilities of transformers [30–33].

Despite rapid advancements, a noticeable gap exists in the literature regarding comprehensive reviews specifically focused on the role and effectiveness of transformer-based architectures (ViTs and CViTs) in neuroimaging for AD diagnosis and MCI progression prediction. Existing surveys often concentrate on earlier DL methods or evaluate models in isolation, without sufficient emphasis on the critical aspects of multimodal data integration, algorithm reproducibility, and implementation frameworks [16, 34–45]. Given that AD is a multifactorial disease, integrating multiple data modalities is crucial for enhancing diagnostic accuracy and predicting progression [46].

The diagnostic complexity of AD stems from its multifaceted pathophysiology, mandating a shift towards multimodal data fusion to enable customized, individualized patient assessment [47]. While earlier DL models, notably CNNs, demonstrated utility in this domain, transformer-based architectures have emerged as a more powerful paradigm [29, 48]. Their fundamental self-attention mechanism is exceptionally well-suited for capturing the intricate, long-range dependencies not only within high-dimensional neuroimaging data but, critically, across heterogeneous data types [49, 50]. This technological superiority underscores the critical need for a systematic survey dedicated to ViT and CViT applications in multimodal AD analysis. However, the current body of literature is conspicuously sparse in reviews that critically appraise the methodologies, fusion strategies, and clinical applicability of these nascent approaches [51]. This review is designed to address this pivotal gap. We provide a comprehensive and critical examination of the contemporary literature on ViTs and CViTs within the AD domain, with a particular emphasis on elucidating the diverse strategies for multimodal data fusion and their implications for diagnostic and prognostic accuracy.

This review aims to bridge this gap by providing a systematic and comprehensive examination of recent studies (between 2021 and 2025) utilizing ViT and CViT models for AD research. The key objectives are to:

- Synthesize recent advances in the architectures and applications of ViT and CViT for the tasks of AD classification, MCI progression prediction, and early-stage disease detection.
- Critically evaluate the documented strengths and limitations of the architectures of these transformer-based approaches specifically within the AD diagnostic context.
- Examine the strategies employed for integrating multimodal data, characterizing how various information sources, including neuroimaging modalities (MRI, PET, DTI, etc.), clinical assessments, genetic profiles, and molecular biomarkers, are combined in pursuit of a holistic AD assessment.
- Characterize the reported implementation details across the selected studies, specifically documenting the computational frameworks utilized, assessing the reproducibility of the presented algorithms, and summarizing the performance evaluation metrics used to quantify model effectiveness.
- Delineate persistent challenges within this research area and identify promising future directions, with particular attention to enhancing model interpretability, improving computational efficiency, addressing dataset limitations and generalizability, refining multimodal fusion techniques, and ultimately improving the clinical applicability of these advanced models.

**Fig. 1:**
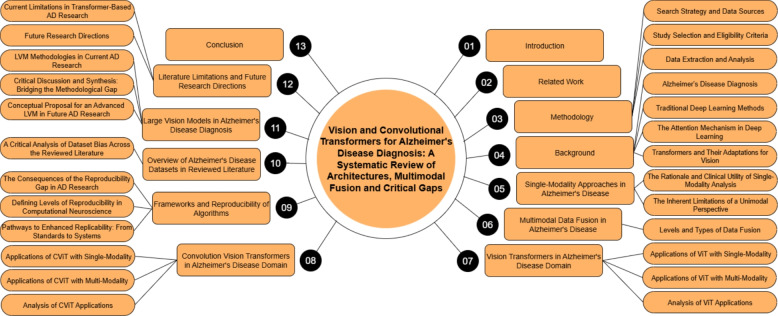
The structure of our study

This review study is organized into several main sections to facilitate a clear and comprehensive exploration of the topic, with its overall structure schematically represented in Fig. 1. Following this introduction, Section 2 discusses previous related studies and positions this review within the existing literature. Section 3 details the systematic methodology employed for study selection and data extraction. Section 4 discusses the necessary background. Section 5 discusses the use and limitations of analyzing individual data types in AD research. Section 6 provides an in-depth analysis of multimodal data fusion strategies used in AD diagnosis. Section 7 systematically reviews studies utilizing ViTs for AD classification and MCI progression prediction. Section 8 systematically reviews studies utilizing CViTs for AD classification and MCI progression prediction. Section 9 explores the frameworks and reproducibility of codes in the reviewed studies. Section 10 reviews the datasets used for AD research. Section 11 discusses how LVM principles are currently utilized in the reviewed ViT/CViT literature and proposes a conceptual advanced LVM architecture for future AD research, focusing on pretraining and multimodal fusion. Section 12 discusses the limitations and future research directions. Finally, Section 13 presents the conclusion of the review.

## Related work

The advent of artificial intelligence (AI) has inaugurated a new epoch in AD research, offering powerful computational approaches to address persistent challenges in early-stage classification, MCI conversion forecasting, and multimodal data analysis [44, 52]. This technological evolution—from traditional ML to DL and now to transformer-based models—has been charted in a substantial body of review literature. Initial syntheses meticulously evaluated classical ML paradigms, such as Support Vector Machines (SVM) and Random Forests, which relied on hand-engineered features derived from neuroimaging data like regional volumes or cortical thickness [13, 14, 53, 54]. The field subsequently pivoted to DL, with extensive reviews detailing the capacity of architectures like CNNs to autonomously extract salient features from raw imaging data [51, 55, 56] and Recurrent Neural Networks (RNNs) to model longitudinal patterns [16, 40, 43]. A foundational insight from these syntheses is the validated superiority of multimodal data fusion, demonstrating that integrating complementary information from sources like MRI, PET, and clinical assessments yields greater diagnostic power than any unimodal approach [42, 46, 47].

While these reviews have effectively mapped the landscape of what can be termed the ’pre-transformer era’—highlighting enduring needs for better model transparency [57] and standardized benchmarks [51]—they naturally precede the current wave of innovation. Consequently, a conspicuous gap remains in the literature. A critical analysis consolidating the research on ViT and CViT models for AD classification and MCI progression prediction is notably absent. Furthermore, there is a scarcity of reviews that systematically scrutinize crucial implementation details such as algorithmic reproducibility [51, 58], the computational frameworks employed, and the nuanced strategies required for integrating neuroimaging with both structured and unstructured clinical data [44, 52].

Several previous reviews have contributed valuable insights into ML and DL applications for AD diagnosis. For instance, Sharma et al. [16] provided an overview of ML techniques applied to multimodal neuroimaging, emphasizing the necessity of advanced AI approaches, including DL and hybrid models, to improve early detection and classification accuracy. Similarly, Nazir et al. [43] examined DL methodologies, particularly CNNs and RNNs, for analyzing MRI, PET, and fMRI data. The study highlighted existing challenges, such as the need for improved model interpretability, standardized evaluation benchmarks, and enhanced multimodal data fusion techniques. Odusami et al. conducted in [42] systematically assessed ML approaches for classifying AD stages using multimodal neuroimaging data. The study reviewed 47 research studies, evaluating classification techniques, feature fusion strategies, and diagnostic accuracy. The findings underscored the effectiveness of combining MRI, PET, and other imaging modalities to enhance classification performance in distinguishing MCI from AD and its progressive stages. However, the study emphasized the need for further research to improve clinical applicability, algorithmic reliability, and multimodal fusion standardization.

Another study, Toumaj et al. [40], systematically examined DL applications in AD classification, focusing on models applied to both single-modality and multimodal neuroimaging data. It also explored the frameworks used in implementing these techniques, identifying research gaps, particularly in XAI and robust multimodal integration. The review proposed future research directions, including enhanced data fusion techniques, the development of more interpretable DL models, and addressing security concerns in AI-driven AD diagnostics. Moreover, Valizadeh et al. [44] provided a systematic review of DL strategies for predicting MCI progression to AD, analyzing studies from 2013 to 2023. The review evaluated various DL architectures, including CNNs, RNNs, autoencoders, and ensemble models, focusing on MRI and clinical data. The study highlighted challenges such as subtle structural brain changes, the necessity of XAI, and the importance of integrating multimodal datasets for improved prediction accuracy. It also emphasized the need for standardized evaluation metrics to enhance the reliability of AI-based diagnostic frameworks.

Further, Hcini et al. [57] investigated the role of DL in early AD detection, specifically comparing CNNs and ViTs in their ability to analyze neuroimaging data. The study discussed recent advancements in both architectures, highlighting the advantages of ViTs in capturing long-range dependencies in brain imaging data. However, it also pointed out the need for data standardization, explainability, and multimodal integration to improve AD diagnostic accuracy. Additionally, Bravo et al. [58] focused on ViTs and CNNs for AD classification using 3D MRI scans, assessing the reproducibility of algorithms and the frameworks used in their implementation. The review followed a structured methodology, analyzing 44 selected studies and identifying key trends in DL-based AD diagnosis. The findings reinforced the significance of multimodal fusion techniques, the necessity for model interpretability, and the development of standardized AI benchmarks for improved AD classification performance. Finally, Nagarajan et al.[45] reviewed recent advancements in DL for AD classification, particularly emphasizing models applied to single-modality and multimodal neuroimaging data.

To contextualize the contribution of this review within the broader body of literature, Table 1 provides a comparative overview of previously published surveys on the application of AI in AD research. This table systematically contrasts key characteristics across these studies, including the reference, year of publication, the temporal coverage of each review, and the number of primary studies analyzed. Furthermore, it delineates the scope of each review in terms of its focus on ViTs and CViTs, specifically regarding their application to AD classification and MCI progression prediction. In addition to temporal and model-specific coverage, the table categorizes earlier surveys based on the types of data modalities considered, namely, single-modality inputs (e.g., structural MRI (sMRI)), multimodal neuroimaging (e.g., MRI combined with PET), and integrated multimodal approaches combining imaging with clinical or biological data. For consistency, the term "clinical data" is interpreted broadly to include demographic information, patient history, cognitive assessments, fluid biomarkers, and genetic profiles that contribute to a holistic understanding of the disease.

A critical analysis of the comparative data in Table 1 reveals several conspicuous voids in the existing survey literature, which this review is designed to fill. Foremost is a technological lag: the vast majority of prior reviews concentrate on pre-transformer architectures, with only a few recent publications beginning to touch upon ViTs and CViTs, yet none offer the comprehensive scope presented here. Second, while AD classification is a frequent topic, a consolidated analysis of MCI-to-AD progression prediction—a clinically vital prognostic task—is notably underdeveloped in the context of these newer models.

Perhaps most significantly, the table highlights a near-universal omission of practical and implementation-level details. Crucial aspects such as the reproducibility of algorithms and the specific computational frameworks used are rarely scrutinized. Similarly, a deep, critical examination of multimodal fusion strategies, particularly those integrating neuroimaging with non-imaging clinical and biological data, remains a significant gap. By systematically addressing not only the models and modalities but also these critical, often-overlooked dimensions, our review provides a uniquely holistic and practical assessment of the field.

**Table 1:** A comparison of our review to the existing reviews

Review study	Year	Period	No. of studies	AD classification	MCI prediction	ViTs	CViTs	Single-modal data	Multimodal (Neuroimaging)	Multimodal (Neuroimaging & clinical data)	Fusion strategies	Algorithms reproducibility	Frameworks
[16]	2022	Past 3 decades	230	$\checkmark$					$\checkmark$		$\checkmark$		
[42]	2024	(2016–2022)	47	$\checkmark$	$\checkmark$			$\checkmark$	$\checkmark$	$\checkmark$	$\checkmark$		
[43]	2024	–	147	$\checkmark$					$\checkmark$				
[40]	2024	(2017–2024)	50	$\checkmark$				$\checkmark$	$\checkmark$				
[44]	2024	(2013–2023)	78		$\checkmark$			$\checkmark$		$\checkmark$			
[57]	2024	(2019–2024)	61	$\checkmark$		$\checkmark$		$\checkmark$	$\checkmark$				
[58]	2024	(2016–2024)	44	$\checkmark$		$\checkmark$	$\checkmark$	$\checkmark$				$\checkmark$	$\checkmark$
[45]	2025	(2005–2024)	34	$\checkmark$				$\checkmark$	$\checkmark$				
Our	2025	(2021–2025)	68	$\checkmark$	$\checkmark$	$\checkmark$	$\checkmark$	$\checkmark$	$\checkmark$	$\checkmark$	$\checkmark$	$\checkmark$	$\checkmark$

## Methodology

This systematic review adheres to the methodological framework for conducting literature reviews [59] and is reported in accordance with the Preferred Reporting Items for Systematic Reviews and Meta-Analyses (PRISMA) 2020 statement [60]. The PRISMA guidelines provide a robust, evidence-based checklist designed to ensure the transparent and complete reporting of systematic reviews, which is critical for methodological soundness. The primary objective is to evaluate the effectiveness of ViT and CViT models in AD classification and MCI conversion prediction. This assessment specifically examines model applications based on different input data types: single-modality data (e.g., sMRI, fMRI, cognitive scores, PET, and clinical notes), multimodal neuroimaging (e.g., MRI combined with PET), or combined neuroimaging and clinical data (i.e., structured and unstructured). For this review’s categorization, "clinical data" encompasses information gathered through direct patient interaction (evaluations, cognitive tests, history, demographics) and laboratory tests (fluid biomarkers, genetics) that contribute to understanding the patient’s condition and prognosis, especially when integrated with imaging findings [61, 62]. By systematically analyzing existing research across these data configurations, our objective is to identify current knowledge gaps and propose future research directions to enhance the applicability of AI-driven neuroimaging techniques in AD diagnostics.

### Search strategy and data sources

Recognizing the need for a comprehensive and reproducible synthesis of the literature, a rigorous search strategy was devised to retrieve studies published only in peer-reviewed journals. Multiple scientific databases were systematically searched (see Table 2) to ensure broad coverage of significant research contributions, a practice recommended to minimize publication bias [63]. The search was conducted on February 20, 2025, utilizing a meticulously constructed query designed for high sensitivity and methodological precision.

This query was built from four conceptual domains, enriched with an expanded set of keywords, synonyms, and common abbreviations to maximize retrieval:

- *Disease and patient cohort*: To encompass the target population, terms included "Alzheimer’s disease", "AD", "Dementia", "Mild Cognitive Impairment", "MCI", terms related to prognosis such as "pMCI" and "sMCI", and broader descriptors like "Cognitive Decline" and "Neurodegenerative".
- *Core technology and architecture*: To identify the specific models, keywords included "Vision Transformer", "ViT", "Convolutional Vision Transformer", "CViT", prominent variants like "Swin Transformer", the underlying mechanism of "self-attention", and more general terms such as "Transformer-based model" and "Attention-based".
- *Primary objective and task*: To capture the application’s goal, the query used a range of terms including "classification", "prediction", "diagnosis", "detection", "prognosis", and "staging".
- *Data and methodology*: To ensure coverage of data handling, a broad set of ancillary keywords such as "multimodal", "multimodality", "data fusion", "integration", "neuroimaging", "MRI", "PET", "fMRI", "clinical data", and "biomarkers" were used in supplementary searches and to manually screen results.

These keyword domains were integrated using Boolean operators to form a robust foundational query. This core string was designed to be broad enough to capture all relevant papers while remaining focused on the intersection of the disease, the technology, and the task. The final, expanded search string was formulated as follows, with minor syntactical adjustments made for each database:

*("Alzheimer’s disease" OR "AD" OR "Dementia" OR "Mild Cognitive Impairment" OR "MCI" OR "pMCI" OR "sMCI" OR "Cognitive Decline" OR "Neurodegenerative") AND ("Vision Transformer" OR "ViT" OR "Convolutional Vision Transformer" OR "CViT" OR "Swin Transformer" OR "Transformer-based model" OR "self-attention" OR "Attention-based") AND ("Classification" OR "Prediction" OR "Diagnosis" OR "Prognosis" OR "Detection" OR "Staging")*

The search covered five major databases, and the results obtained from each data source are summarized in Table 2. The review focuses on studies that present novel approaches to AD classification, MCI progression prediction, or both using ViTs or CViTs in the context of neuroimaging and different clinical data.

**Table 2:** Literature search results

Database	Filters applied for the search	Number of publications found	Number of publications selected
Scopuse	Title, abstract and keywords	116	21
Web of Science	All Text	121	11
ScienceDirect	All Text	232	25
IEEExplore	All Text	60	11
PubMed	All Text	35	–

### Study selection and eligibility criteria

Following the collection of relevant studies, a systematic screening process was carried out. First, duplicate records were removed to ensure uniqueness. Next, the titles and abstracts of the remaining studies were screened to eliminate research that was outside the scope of this review. The full texts of the shortlisted studies were then carefully examined to determine their eligibility based on predefined inclusion and exclusion criteria (outlined in Table 3). This multi-stage screening is a standard and critical component of systematic reviews, designed to progressively refine the study pool to only the most relevant and high-quality evidence [60].

The final selection of studies was made through a consensus-based decision by two independent reviewers to enhance the objectivity and reliability of the selection process and to minimize selection bias [63]. This step is particularly crucial in a field where methodological rigor can vary, and it helps to ensure that the final synthesis is based on a consistently evaluated set of studies. Critically, during the full-text review, studies were carefully assessed for potential methodological flaws such as data leakage, a significant concern in ML-based medical research that can lead to biased and overly optimistic performance estimates [51].

**Table 3:** Overview of inclusion and exclusion criteria considered

Parameters	Inclusion criteria	Exclusion criteria
Investigation	Studies that include AD classification or MCI conversion prediction and include neuroimaging data or clinical data.	Studies that do not include AD classification or MCI conversion prediction and do not include neuroimaging data or clinical data.
Type of AI algorithm	Studies that incorporate any form of ViTs or CViTs in their architectures.	Studies that exclude all types of ViTs and CViTs, relying solely on traditional DL methods (e.g., CNN, 3DCNN) or only ML techniques.
Type of Study	Studies published in journals only.	Studies are not published in journals.
Experimental results	Studies including experimental results	Studies that have not reported performance metrics (e.g., accuracy, sensitivity, specificity, etc.), Studies that lack sufficient information to extract relevant details, reviews, patents, conference abstracts, book chapters, surveys, preprints.
Year of the Study	Studies published between 2021 and 2025 (last five years).	Studies that are not in this custom range.
Language of the Study	Studies written in English.	Studies written in a language other than English.
Database	Studies found in the databases in Table 2	Studies not found in the mentioned databases.

### Data extraction and analysis

**Fig. 2:**
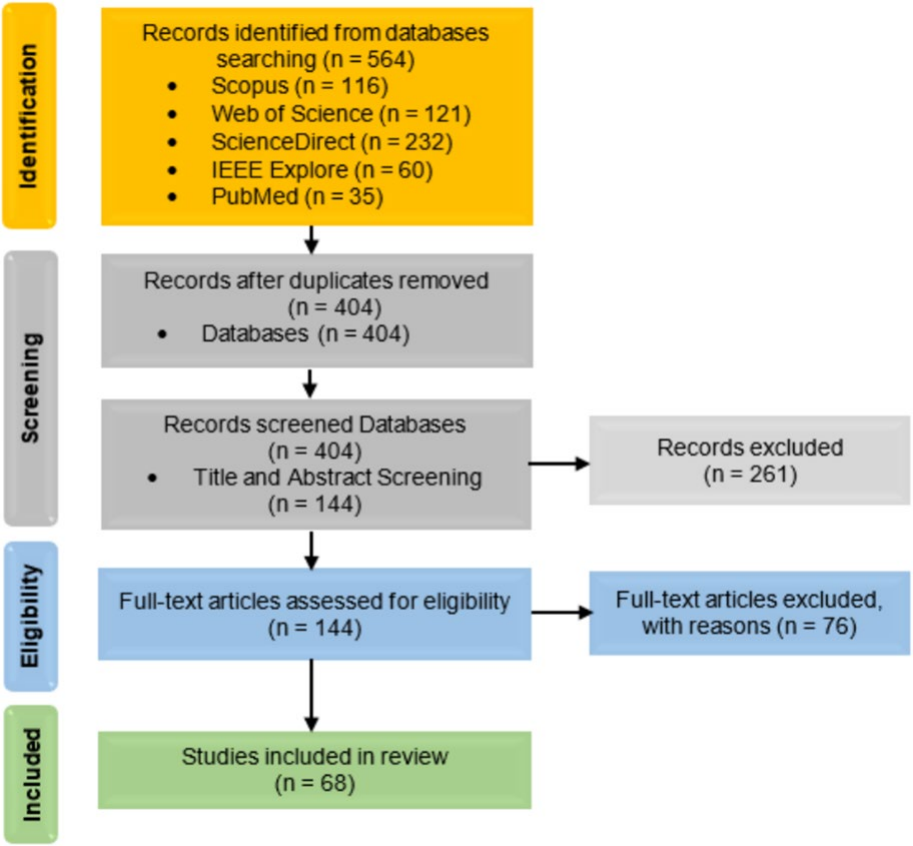
PRISMA flow diagram of the search and exclusion process

For each study that met the inclusion criteria, relevant information was systematically extracted, including: publication year, diagnostic focus (AD classification or MCI conversion prediction), modality configuration (Single, Multi), medical modality utilized (MRI, PET, DTI, CT, CSF, Genetic etc.), fusion strategies (for multimodal inputs), dataset specifics, AI technique applied, classification categories, internal validation, external validation, and performance evaluation metrics (e.g., accuracy, AUC, sensitivity, specificity). This systematic approach to data extraction is essential for creating a structured evidence base and mitigating the risk of reviewer bias [63]. Following a systematic literature search using predefined search strings across multiple information sources, a total of 564 studies were initially identified, as outlined in Table 2. The systematic review process involved several key stages, which are detailed below and illustrated in Fig. 2.

- *Defining search parameters*: Establishing relevant search strings and selecting appropriate databases.
- *Conducting the search*: Executing the predefined search queries across selected databases, yielding 564 studies.
- *Duplicate removal*: Identifying and consolidating duplicate records appearing in multiple databases, reducing the dataset to 404 unique studies.
- *Title and abstract screening*: Evaluating study titles and abstracts for relevance, resulting in the acceptance of 144 studies and the exclusion of 261.
- *Full-text review*: Conducting an in-depth assessment of the full text, ultimately selecting 68 studies and rejecting 76.

**Fig. 3:**
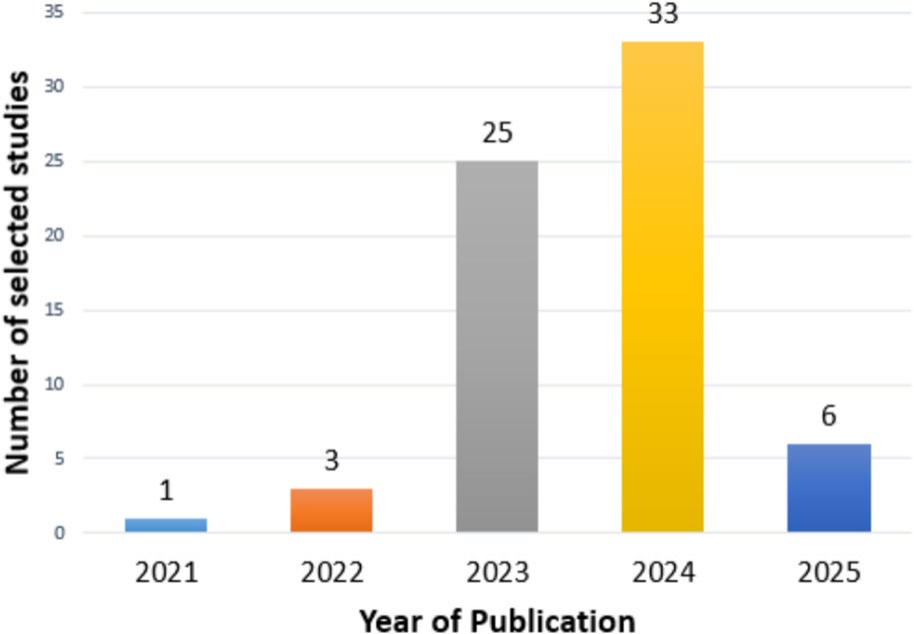
The bar chart displays the number of selected studies versus publication years

The final 68 studies included in this review, selected through a systematic screening process, were published in high-impact international journals, indicating their scientific reliability. Figure 3 further illustrates the distribution of these selected studies across the five years from 2021 to 2025, providing insight into the temporal research trends within the domain of AD diagnosis. This distribution highlights a growing research interest in AD diagnosis using advanced DL techniques, particularly during 2023 and 2024.

## Background

This section provides essential background information crucial for understanding the application of advanced computational techniques, particularly transformer-based models, in AD research. First, Subsect. 4.1 details the clinical continuum of AD, outlining its progressive stages from Cognitively Normal (CN) through various phases of MCI (Early MCI (EMCI), MCI, Late MCI (LMCI)) to AD dementia. It also describes the primary neuroimaging (sMRI, PET, DTI, fMRI), fluid biomarker (CSF, blood), and genetic (APOE, GWAS) data modalities commonly employed for jointly diagnosis and their respective roles in identifying structural, functional, molecular, and genetic changes associated with the disease. Building on this, Subsect. 4.2 offers a concise overview of traditional DL methods, namely CNNs, including their 3D variants, and RNN architectures such as LSTMs and GRUs, which formed the bedrock for analyzing neuroimaging and timeseries clinical data before the advent of transformers. Subsequently, Subsect. 4.3 provides a focused review of the attention mechanism, a pivotal innovation that underpins the capabilities of transformer models by enabling dynamic weighting of input features. Finally, Subsect. 4.4 describes the Transformer and its adaptations for computer vision, particularly the ViT and CViT, detailing their core components and operational principles. This foundational knowledge facilitates a deeper comprehension of the methodologies, challenges, and advancements discussed in the subsequent systematic review sections.

### Alzheimer’s disease diagnosis

**Fig. 4:**
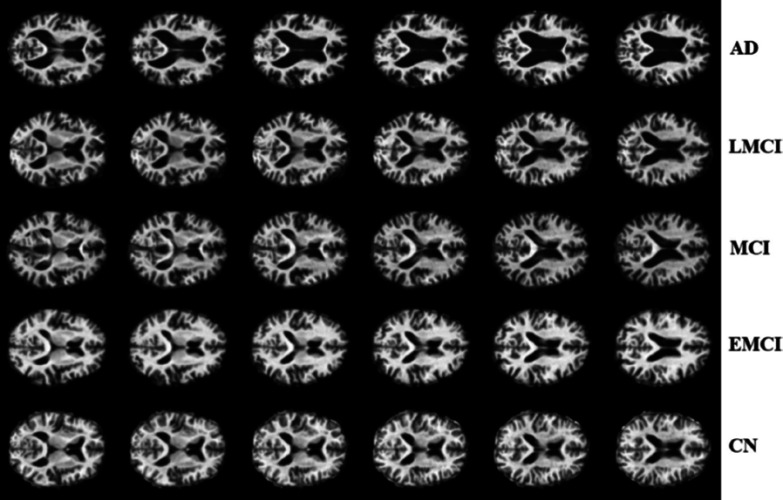
MRI in CN, EMCI, MCI, LMCI, and AD. Figure adapted from [58]

AD is a progressive neurodegenerative disorder characterized by a gradual decline in cognitive functions that eventually impairs daily functioning. AD pathology develops over a long preclinical period and advances through a continuum of stages with evolving cognitive impairments and underlying biological changes, as defined by the NIA-AA research framework [17]. The key stages are:

**CN:** This stage represents individuals with no significant cognitive decline for their age [64]. Although clinically silent, underlying pathologies like A$\beta$ plaques and tau tangles may be present and detectable by advanced biomarkers, making this group an essential baseline for longitudinal studies [65] (see Fig. 4, row CN).

**EMCI:** This stage marks the onset of subtle, measurable cognitive changes that do not significantly impair daily functioning [66]. Neuroimaging may reveal early signs like mild hippocampal atrophy, making it a critical stage for intervention research (see Fig. 4, row EMCI).

**MCI:** In this stage, cognitive decline becomes more clinically evident, affecting functions like memory and attention, though most daily activities remain manageable [67]. MRI often shows progressive atrophy, and around 10-15% of individuals with MCI progress to AD dementia annually [68] (see Fig. 4, row MCI).

**LMCI:** As a transitional phase to dementia, cognitive impairments in LMCI begin to interfere with complex tasks, and neuroimaging reveals more widespread cortical atrophy (see Fig. 4, row LMCI).

**AD Dementia:** This stage is defined by severe cognitive and functional impairment that fulfills dementia criteria [10]. It involves severe memory deficits, language difficulties, and emergent behavioral symptoms, with MRI showing extensive brain atrophy. Patients eventually require full-time care, and while symptomatic treatments exist, disease-modifying therapies show promise when initiated early [69] (see Fig. 4, row AD).

Accurate AD diagnosis requires a comprehensive, multimodal approach integrating clinical assessments, medical imaging (MRI, PET), biomarkers (CSF, blood), and genetic risk analysis (e.g., *APOE* $\varepsilon$4). Combining these data sources is crucial for improving diagnostic precision and supporting therapeutic development [17].

#### Structural imaging in AD

Structural neuroimaging provides a macroscopic view of brain anatomy, allowing for the quantification of morphological changes linked to neurodegeneration [23]. The primary modalities are sMRI and Computed Tomography (CT).

***Medical and clinical significance***

The central role of structural imaging in AD diagnosis lies in its ability to detect and map patterns of cerebral atrophy. High-resolution sMRI is particularly sensitive to the hallmark volumetric losses of the disease, with the earliest and most significant finding often being atrophy of the medial temporal lobe, including the hippocampus [11, 70]. As AD advances, atrophy becomes more widespread, accompanied by ventricular enlargement (Fig. 4). To enable precise, quantitative analysis beyond visual inspection, techniques such as Voxel-Based Morphometry (VBM) [71] and automated segmentation algorithms [72] are used to measure regional brain volumes and gray matter density. Furthermore, longitudinal sMRI studies have confirmed that tracking these volumetric changes over time, particularly the rate of hippocampal shrinkage, serves as a powerful predictive biomarker for MCI-to-AD conversion [73].

Beyond gray matter, Diffusion Tensor Imaging (DTI) assesses the microstructural integrity of white matter tracts [74]. In AD, DTI reveals degradation of key neural pathways, offering insight into the structural disconnection underlying cognitive deficits [75, 76]. CT remains valuable for excluding other pathologies like stroke or tumors that can mimic dementia [10].

***AI and computational perspective***

From an AI perspective, structural neuroimaging data presents distinct challenges and opportunities related to its dimensionality and representation.

**Fig. 5:**
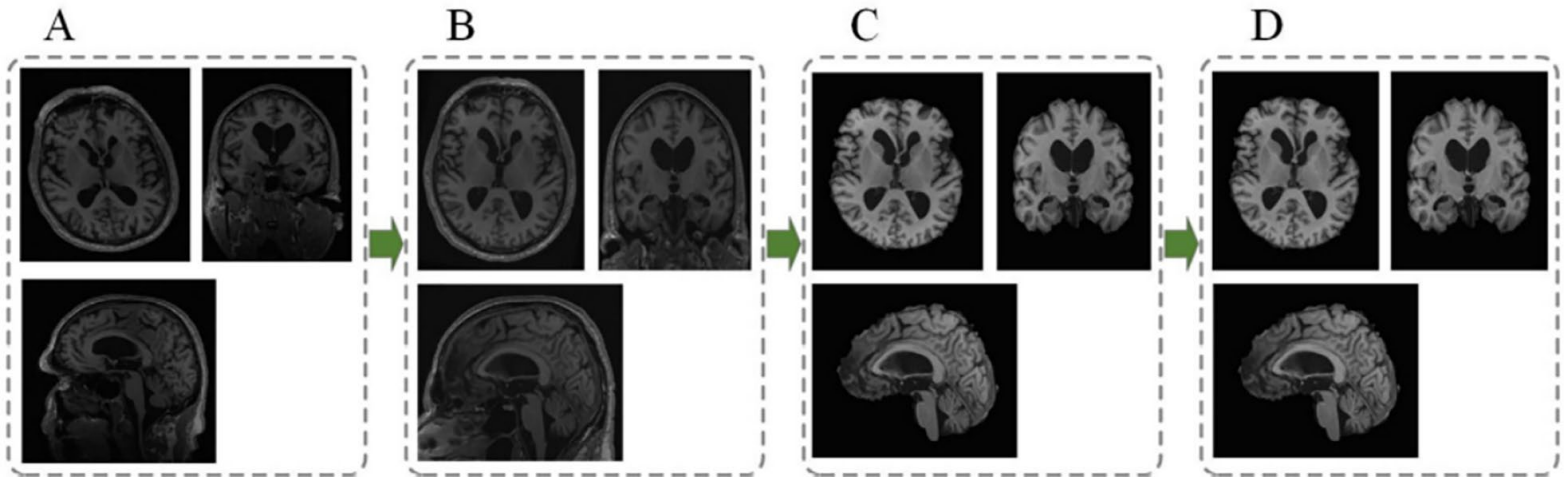
MRI images preprocessing pipeline.** A**: Original brain MRI images. **B**: MRI images after space normalization. **C**: MRI images after skull dissection. **D**: MRI images after N4 bias field correction. Figure adapted from [81]

- *Dimensionality and preprocessing*: A typical T1-weighted sMRI scan is a 3D volumetric dataset. Before use in an AI pipeline, this raw data requires a standardized preprocessing cascade to ensure consistency, including intensity normalization, spatial registration to a common template like MNI, and skull stripping to isolate brain tissue [77–79]. This standard pipeline is shown in Fig. 5.
- *Data representation for AI models*: The preprocessed 3D volume can be fed into AI models using several strategies:
  - *Full 3D volume*: The most complete representation, ideal for 3D CNNs and 3D ViTs, but computationally intensive [51].
  - *Sequence of 2D slices*: A common approach that treats the 3D volume as a sequence of 2D slices, enabling the use of powerful, pre-trained 2D models but risking the loss of inter-slice context [43].
  - *Voxel-based representation*: A classical ML approach where features are extracted from each voxel to create feature maps for classification [71].
  - *Region of Interest (RoI) analysis*: A knowledge-guided method that focuses on specific anatomical regions known to be affected by AD (e.g., hippocampus), reducing dimensionality and concentrating the model on clinically relevant areas [80].

#### Functional imaging in AD

Functional imaging techniques provide a dynamic view of the brain’s physiological and metabolic processes, offering insights complementary to static anatomical information. The principal modalities are PET and functional Magnetic Resonance Imaging (fMRI).

***Medical and clinical significance***

Functional imaging is pivotal for detecting the molecular and physiological consequences of AD, often before significant structural atrophy is apparent. PET imaging, using specific radiotracers, can directly visualize the core proteinopathies of AD [12].

**Fig. 6:**
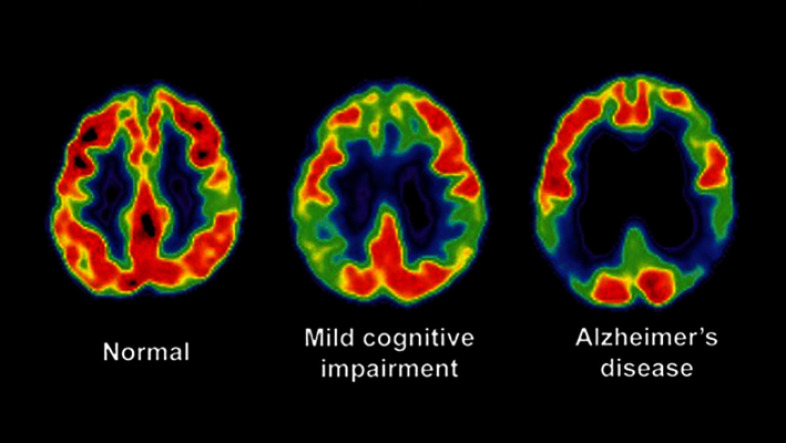
Illustrative Amyloid-PET scans showing the progressive accumulation of amyloid-beta plaques across different stages of AD. The increasing intensity of the color signal (typically red/yellow) from CN to MCI and finally to AD corresponds to a higher cerebral amyloid burden

- *Amyloid-PET* maps the brain’s amyloid-beta plaque burden, a crucial biomarker for early risk assessment and diagnosis, as shown in Fig. 6 [82, 83].
- *Tau-PET* visualizes neurofibrillary tangles. The spatial pattern of tau pathology correlates strongly with cognitive decline, making it an excellent marker for disease staging [84, 85].
- *Fluorodeoxyglucose PET (FDG-PET)* measures regional cerebral glucose metabolism. AD is associated with a characteristic pattern of hypometabolism, reflecting synaptic dysfunction [86, 87].

**Functional MRI (fMRI)** assesses brain activity indirectly by detecting changes in blood-oxygen-level-dependent (BOLD) signals. In AD research, resting-state fMRI (rs-fMRI) is widely used to investigate functional connectivity. A consistent finding in AD is the disruption of large-scale brain networks, most notably the Default Mode Network (DMN), which is implicated in memory consolidation [88, 89].

***AI and computational perspective***

The dynamic nature of functional imaging data introduces unique computational considerations.

- **Dimensionality and preprocessing:**
  - *PET* scans, like sMRI, are typically processed as 3D volumes. Preprocessing involves spatial normalization and co-registration to a corresponding sMRI scan for anatomical context [90].
  - *fMRI* data is inherently 4D (H x W x D x Time). Its preprocessing is more complex, including steps like motion correction and temporal filtering to remove physiological noise [91].
- **Data representation for AI models:**
  - *PET*: Can be handled using the same strategies as sMRI (full 3D volumes, 2D slices, RoI-based quantification).
  - *fMRI*: The temporal dimension offers several representation strategies, including:
    1. *Functional connectivity (FC) matrices*: The most common approach, where a correlation matrix is computed from RoI time-series signals to represent the strength of connectivity between regions. This matrix can be used as input for a CNN or GNN [92].
    2. *Dynamic FC (dFC)*: A sliding-window approach can generate a sequence of FC matrices, ideal for RNNs or Transformers to model network evolution [93].
    3. *Direct spatio-temporal data*: Using the raw 4D fMRI data is computationally demanding but allows advanced architectures to learn complex dynamic patterns without predefined RoIs [94].

#### Clinical assessments and fluid biomarkers in AD

While neuroimaging reveals structural and functional changes, clinical assessments and fluid biomarkers are essential for capturing the disease’s clinical manifestation and underlying molecular pathology.

***Medical and clinical significance***

Clinical assessments are fundamental for quantifying the impact of the disease on an individual’s cognition and daily functioning.

- *Cognitive screening*: Instruments like the Mini-Mental State Examination (MMSE) [78] and Montreal Cognitive Assessment (MoCA) [95] provide a rapid, quantitative measure of global cognitive status.
- *Disease staging*: Comprehensive tools like the Clinical Dementia Rating (CDR) scale [96] are used to stage disease severity by integrating performance across multiple cognitive and functional domains.

Direct molecular insights are derived from analyzing biomarkers in CSF and blood.

- *CSF biomarkers*: Analysis of CSF reveals a characteristic AD signature involving decreased A$\beta$42 (amyloid) and increased total tau (t-tau) and phosphorylated tau (p-tau), reflecting plaque deposition and neuronal injury [15, 97].
- *Blood-based biomarkers*: Less invasive blood-based plasma biomarkers, such as specific p-tau species (p-tau217, p-tau181) and neurofilament light chain (NfL), have emerged as highly promising alternatives for detecting and monitoring AD-related changes [98, 99].

***AI and computational perspective***

Unlike high-dimensional imaging data, clinical assessments and biomarkers typically constitute low-dimensional, structured tabular data, presenting distinct computational needs.

**Fig. 7:**
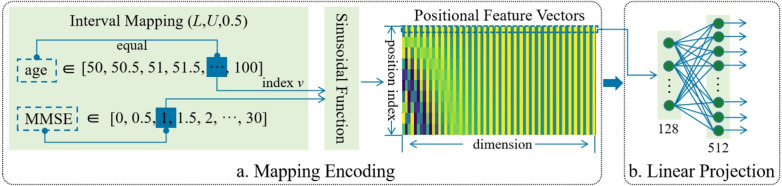
Architecture of the linear encoder for non-image data. Figure adapted from [102]

- *Dimensionality and preprocessing*: This tabular data is organized as a feature vector per patient, containing continuous and categorical/ordinal values. Standard preprocessing is essential and includes handling missing values (e.g., via imputation), scaling continuous features, and encoding categorical features (e.g., via one-hot encoding) [100].
- **Data representation for AI models:**
  - The preprocessed features are concatenated into a single feature vector.
  - This vector is an ideal input for feed-forward neural networks like a Multi-Layer Perceptron (MLP) [101].
  - In multimodal transformer models, this feature vector is typically projected into a higher-dimensional embedding space to create a single "non-image token," as shown in Fig. 7. This token can then be processed alongside image-derived tokens within the transformer’s self-attention mechanism [49, 102].

#### Genetic factors in AD

Genetic predisposition plays a pivotal role in determining AD risk and influencing its pathological course, with factors differing for early-onset versus late-onset forms.

***Medical and clinical significance***

- *Early-Onset familial AD (EOAD)*: A small fraction of AD cases are caused by highly penetrant mutations in one of three genes: APP, PSEN1, and PSEN2. These mutations directly impact amyloid processing and almost invariably lead to aggressive, early-onset disease [3, 103].
- *Late-Onset AD (LOAD)*: For the vast majority of cases, genetics confers risk rather than causation. The most significant genetic risk factor for LOAD is the Apolipoprotein E (APOE) gene, which has three common alleles: $\varepsilon$4 (high risk), $\varepsilon$3 (baseline risk), and $\varepsilon$2 (protective) [104, 105].
- *Other LOAD risk variants and polygenic risk*: Genome-Wide Association Studies (GWAS) have identified dozens of other common genetic variants that are associated with smaller changes in LOAD risk [106, 107]. The cumulative effect of these variants can be aggregated into a Polygenic Risk Score (PRS), which provides a more comprehensive measure of an individual’s genetic liability for developing AD [108].

***AI and computational perspective***

Genetic data, being fundamentally different from imaging, introduces unique computational requirements for preprocessing and integration.

- **Dimensionality and preprocessing:**
  - *APOE status*: This is a low-dimensional categorical feature, typically encoded as the number of $\varepsilon$4 alleles (0, 1, or 2).
  - *GWAS/SNP data*: This is high-dimensional categorical data. The primary preprocessing step is dimensionality reduction, most effectively achieved by calculating a Polygenic Risk Score (PRS). A PRS reduces millions of SNPs into a single, continuous variable that is readily usable by AI models.
- **Data representation for AI models:**
  - The processed genetic information (APOE count or PRS) is treated as a single feature in a patient’s tabular data vector.
  - Similar to other clinical features, this genetic variable can be projected into a high-dimensional embedding space to form a "genetic token," which can be integrated within a transformer architecture [49, 109].

### Traditional deep learning methods

Prior to the adoption of transformers, CNNs and RNNs were the dominant DL paradigms for neuroimaging analysis in AD research. CNNs excel at processing grid-like data by using learnable filters to automatically and hierarchically extract spatial features, from simple edges to complex patterns [23, 110, 111]. Key components include convolutional layers, non-linear activation functions (e.g., ReLU), pooling layers for dimensionality reduction, and fully connected layers for final classification [112]. For volumetric data like MRI, 3D CNNs use 3D kernels to directly capture inter-slice spatial relationships, a technique that proved effective for AD classification [113–115].

For sequential data, such as longitudinal clinical scores or fMRI time-series, RNNs were the traditional choice [116]. However, standard RNNs struggle with long-term dependencies due to vanishing and exploding gradients [117, 118]. To mitigate this, Long Short-Term Memory (LSTM) networks [119] introduced a memory cell and gating mechanisms (forget, input, output) to regulate information flow, enabling the retention of information over long sequences. Gated Recurrent Units (GRUs) [120] offer a simpler, more computationally efficient alternative with only two gates, often achieving comparable performance [121]. For a more detailed background on these classical DL models, readers are guided to excellent surveys on the topic [16, 43, 51, 55].

### The attention mechanism in deep learning

The attention mechanism, a pivotal innovation in DL, allows models to dynamically weigh the importance of different parts of an input, emulating human selective focus [27, 122]. Initially developed for sequence-to-sequence tasks [122], its application has expanded to computer vision and biomedical informatics [123, 124]. It addresses key limitations in traditional architectures, such as the difficulty of RNNs in capturing long-range dependencies and enhances the contextual awareness of CNNs by modeling non-local interactions [124, 125].

**Fig. 8:**
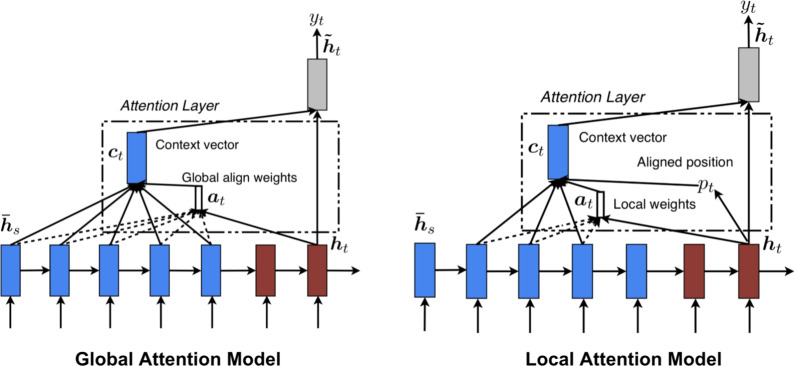
Global and local attention. The global approach considers all source states, while the local approach focuses on a small window. Figure adapted from [127]

**Fig. 9:**
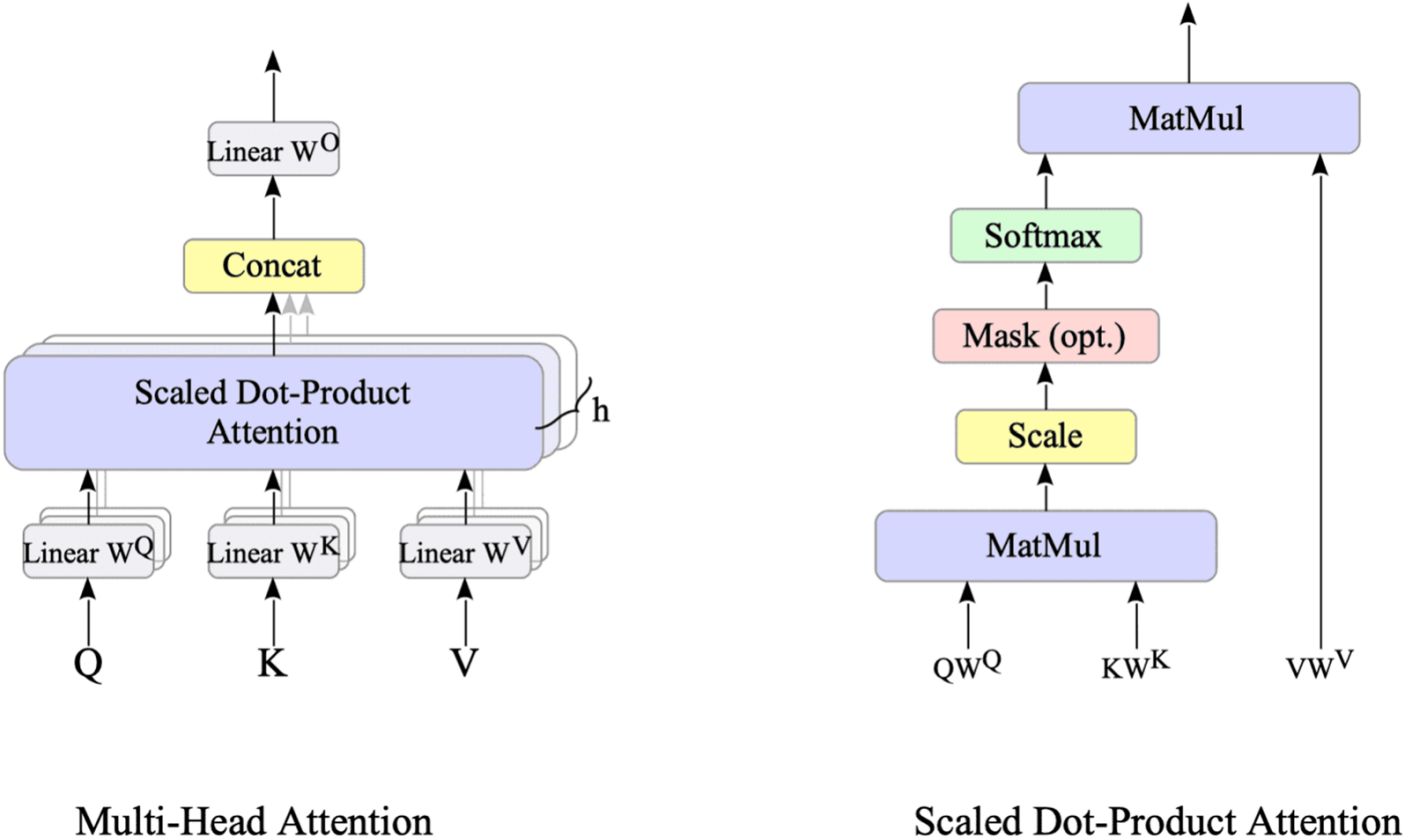
Conceptual diagrams of (left) scaled dot-product attention and (right) multi-head attention. Figure adapted from [128]

**Fig. 10:**
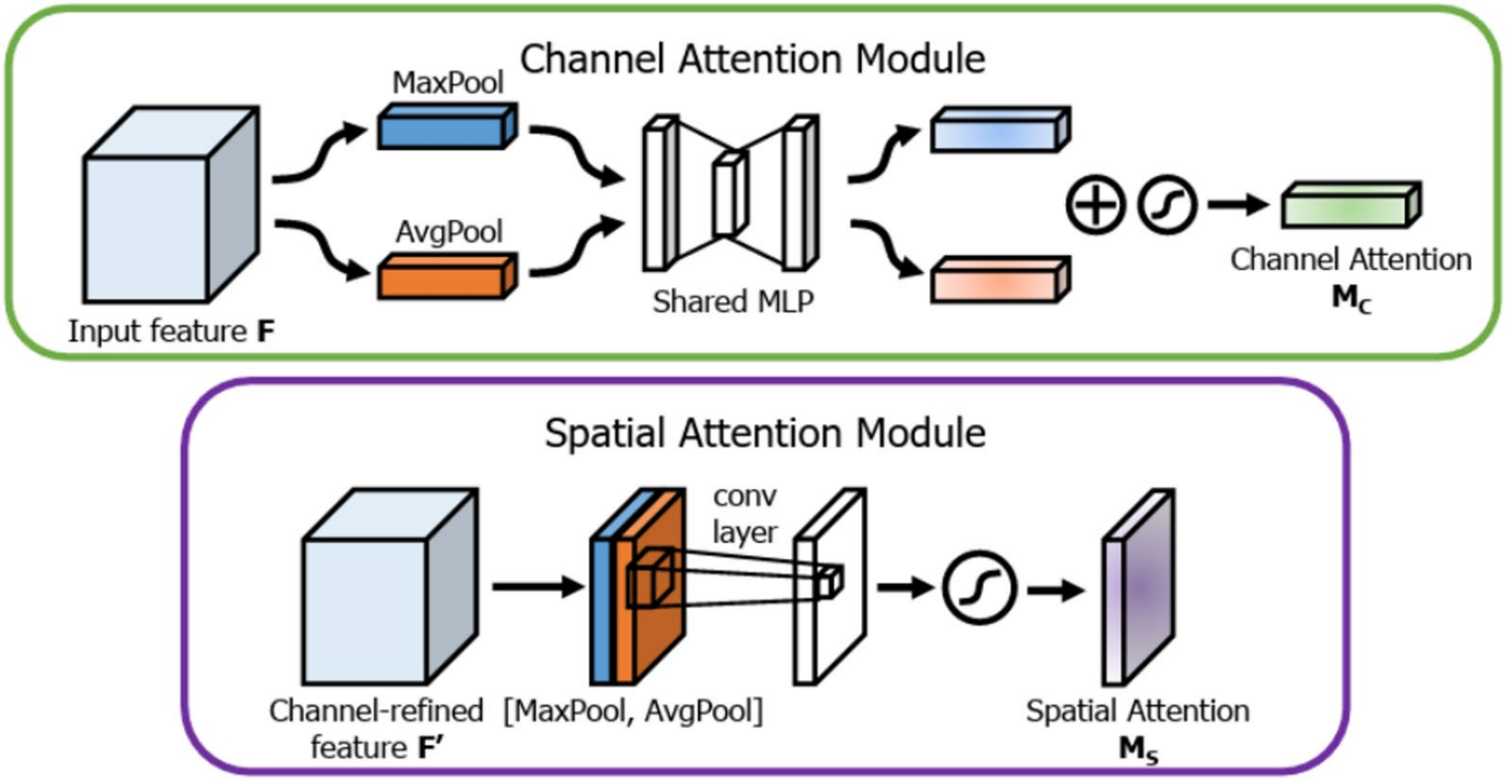
Sequential application of channel and spatial attention modules in the CBAM architecture. Figure adapted from [129]

The primary variants of attention can be systematically categorized as follows:

- **Soft versus hard attention**: This dichotomy relates to the differentiability of the selection process. Soft attention represents a fully differentiable approach where a weighted average of source information is computed. Given a set of *n* source hidden states (or feature vectors) $H = [h_1, \dots , h_n] \in \mathbb {R}^{n \times d_h}$ and a query vector $s_t \in \mathbb {R}^{d_s}$, an alignment model or scoring function *a* first computes a relevance score for each source state:
  1 $$\begin{aligned} e_{ti} = a(s_t, h_i) \end{aligned}$$
  These unnormalized scores are transformed into a probability distribution of attention weights, $\alpha _t = [\alpha _{t1}, \dots , \alpha _{tn}]$, using a softmax function, ensuring that $\sum _{i=1}^{n} \alpha _{ti} = 1$:
  2 $$\begin{aligned} \alpha _{ti} = \frac{\exp (e_{ti})}{\sum _{j=1}^{n} \exp (e_{tj})} \end{aligned}$$
  The resulting context vector $c_t \in \mathbb {R}^{d_h}$ is the weighted sum over all source states, encapsulating the most relevant information for the query $s_t$:
  3 $$\begin{aligned} c_t = \sum _{i=1}^{n} \alpha _{ti} h_i \end{aligned}$$
  Conversely, hard attention, first proposed by Xu et al. [123], formulates the selection as a stochastic process, sampling a single source index $z_t$ from a categorical distribution parameterized by the attention weights: $z_t \sim \text {Categorical}(\alpha _t)$. The context vector is then simply $c_t = h_{z_t}$. Because this sampling operation is non-differentiable, training requires specialized techniques such as policy gradient methods from reinforcement learning (e.g., REINFORCE) or the Gumbel-Softmax reparameterization trick to enable end-to-end training [123, 126].
- **Global versus local attention**: This distinction, introduced by Luong et al. [127] and illustrated in Fig. 8, defines the scope of the alignment function. In **global attention**, the context vector is computed by attending to *all* source hidden states, following the soft attention formulation. While comprehensive, this can be computationally expensive for long sequences. In contrast, **local attention** first predicts an aligned position $p_t$ for the query $s_t$ in the source sequence, often via a small feed-forward network:
  4 $$\begin{aligned} p_t = n \cdot \text {sigmoid}(\textbf{v}_p^T \tanh (W_p s_t)) \end{aligned}$$
  where $W_p$ and $\textbf{v}_p$ are learnable parameters. A computational window of size $2D+1$ is then centered at $p_t$. To provide a soft focus within this hard window, the alignment scores are modulated by a Gaussian distribution centered at $p_t$:
  5 $$\begin{aligned} \alpha _{ti} = \frac{\exp (a(s_t, h_i)) \cdot \exp \left( -\frac{(i - p_t)^2}{2\sigma ^2}\right) }{\sum _{j=p_t-D}^{p_t+D} \exp (a(s_t, h_j)) \cdot \exp \left( -\frac{(j - p_t)^2}{2\sigma ^2}\right) } \end{aligned}$$
  where the standard deviation $\sigma$ is empirically set, often as $\sigma =D/2$. This reduces computational complexity from $\mathcal {O}(n)$ to a constant $\mathcal {O}(D)$.
- **Self-attention (Scaled dot-product attention)**: As the foundational block of Transformers [27], self-attention relates different positions within a single input sequence to compute a new, contextualized representation of that same sequence (Fig. 9). For an input sequence $X \in \mathbb {R}^{n \times d_{\text {model}}}$, the mechanism first projects it into three matrices: Queries ($Q \in \mathbb {R}^{n \times d_k}$), Keys ($K \in \mathbb {R}^{n \times d_k}$), and Values ($V \in \mathbb {R}^{n \times d_v}$), using learnable weight matrices $W_Q, W_K \in \mathbb {R}^{d_{\text {model}} \times d_k}$ and $W_V \in \mathbb {R}^{d_{\text {model}} \times d_v}$:
  6 $$\begin{aligned} Q = XW_Q, \quad K = XW_K, \quad V = XW_V \end{aligned}$$
  The output is a weighted sum of the values, where the weights are derived from the dot-product similarity between queries and keys. A scaling factor, $1/\sqrt{d_k}$, is applied to counteract the tendency for large dot products (which occur when $d_k$ is large) to push the softmax function into regions with vanishingly small gradients, thereby stabilizing training:
  7 $$\begin{aligned} \text {Attention}(Q, K, V) = \text {softmax}\left( \frac{QK^T}{\sqrt{d_k}}\right) V \end{aligned}$$
- **Multi-head attention (MHA)**: MHA enhances self-attention by performing multiple attention computations in parallel (Fig. 9). This allows the model to jointly attend to information from different representation subspaces at different positions [27]. The input is projected into *h* different sets of queries, keys, and values using distinct weight matrices for each head. Let the head dimensions be $d_k' = d_v' = d_{\text {model}}/h$. For each head $i \in \{1, \dots , h\}$:
  8 $$\begin{aligned} \text {head}_i = \text {Attention}(XW_i^Q, XW_i^K, XW_i^V) \end{aligned}$$
  where $W_i^Q, W_i^K \in \mathbb {R}^{d_{\text {model}} \times d_k'}$ and $W_i^V \in \mathbb {R}^{d_{\text {model}} \times d_v'}$ are the projection matrices for the *i*-th head. The outputs of all heads are concatenated, forming a matrix in $\mathbb {R}^{n \times (h \cdot d_v')}$, and then linearly projected back to the original dimension using a final weight matrix $W_O \in \mathbb {R}^{d_{\text {model}} \times d_{\text {model}}}$:
  9 $$\begin{aligned} \text {MultiHead}(Q, K, V) = \text {Concat}(\text {head}_1, \dots , \text {head}_h)W_O \end{aligned}$$
- **Additive versus multiplicative attention**: These terms describe two common instantiations of the alignment scoring function $a(s_t, h_i)$.
  - **Additive attention**, proposed by Bahdanau et al. [122], employs a feed-forward network with a single hidden layer and a $\tanh$ non-linearity. This allows for complex, non-linear interactions to be learned and is effective even when query and key dimensions differ:
    10 $$\begin{aligned} a(s_t, h_i) = \textbf{v}_a^T \tanh (W_s s_t + W_h h_i) \end{aligned}$$
    Here, $W_s \in \mathbb {R}^{d_a \times d_s}$, $W_h \in \mathbb {R}^{d_a \times d_h}$, and $\textbf{v}_a \in \mathbb {R}^{d_a}$ are learnable weight parameters, where $d_a$ is the attention dimension.
  - **Multiplicative (Dot-product) attention**, popularized by Luong et al. [127], is computationally more efficient. It uses a bilinear scoring function:
    11 $$\begin{aligned} a(s_t, h_i) = s_t^T W_a h_i \end{aligned}$$
    where $W_a \in \mathbb {R}^{d_s \times d_h}$ is a learnable weight matrix. The self-attention in Transformers is a special, scaled case of this where $d_s = d_h$ and $W_a$ is an identity matrix.
- **Spatial versus channel attention**: These mechanisms are canonical in modern CNNs, operating on an intermediate feature map $F \in \mathbb {R}^{C \times H \times W}$ to selectively emphasize informative features (Fig. 10).
  - **Spatial attention** focuses on *where* information is located by generating a 2D attention map $M_s(F) \in \mathbb {R}^{1 \times H \times W}$. This map re-weights the feature map spatially:
    12 $$\begin{aligned} F' = M_s(F) \otimes F \end{aligned}$$
    where $\otimes$ denotes element-wise multiplication with broadcasting. A common method to generate $M_s$ is to pool along the channel dimension, concatenate the results, and pass them through a convolutional layer with a sigmoid activation $\sigma (\cdot )$ [125, 129]:
    13 $$\begin{aligned} M_s(F) = \sigma (\text {Conv}^{7 \times 7}([\text {AvgPool}_{\text {ch}}(F); \text {MaxPool}_{\text {ch}}(F)])) \end{aligned}$$
  - **Channel attention** focuses on *what* information is important by generating a 1D attention vector $M_c(F) \in \mathbb {R}^{C \times 1 \times 1}$. The canonical Squeeze-and-Excitation (SE) block [130] generates $M_c$ by first "squeezing" spatial dimensions via global average pooling (GAP) to produce a channel descriptor $z \in \mathbb {R}^C$. This is then "excited" through a bottleneck MLP:
    14 $$\begin{aligned} M_c(F) = \sigma (W_2(\text {ReLU}(W_1(\text {GAP}(F))))) \end{aligned}$$
    where $W_1 \in \mathbb {R}^{(C/r) \times C}$ and $W_2 \in \mathbb {R}^{C \times (C/r)}$ define the MLP, with *r* being the channel reduction ratio. The final output is $F'' = M_c(F) \otimes F$.
- **Cross-attention**: This is a structural adaptation of self-attention, crucial for tasks involving the fusion of information from two distinct input sequences, such as in multimodal learning or encoder-decoder frameworks [27]. Given two sequences, a query sequence $X_1 \in \mathbb {R}^{n_1 \times d_1}$ and a key-value sequence $X_2 \in \mathbb {R}^{n_2 \times d_2}$, cross-attention generates queries from the first sequence and keys/values from the second:
  15 $$\begin{aligned} Q = X_1W_Q, \quad K = X_2W_K, \quad V = X_2W_V \end{aligned}$$
  where $W_Q \in \mathbb {R}^{d_1 \times d_k}$, $W_K \in \mathbb {R}^{d_2 \times d_k}$, and $W_V \in \mathbb {R}^{d_2 \times d_v}$ are the projection matrices. The resulting attention output, which lies in $\mathbb {R}^{n_1 \times d_v}$, is a new representation of the query sequence ($X_1$) that has been contextualized by attending to the most salient information within the key/value sequence ($X_2$).

The integration of these diverse attention mechanisms has profoundly advanced DL capabilities, enabling more nuanced feature extraction and superior context modeling across a wide spectrum of applications.

### Transformers and their adaptations for vision

The Transformer architecture, introduced by Vaswani et al. [27], marked a paradigm shift in sequence modeling. Its design effectively models long-range dependencies by exclusively relying on attention mechanisms, circumventing the sequential processing limitations of recurrent architectures [27, 131].

#### Vision transformer

**Fig. 11:**
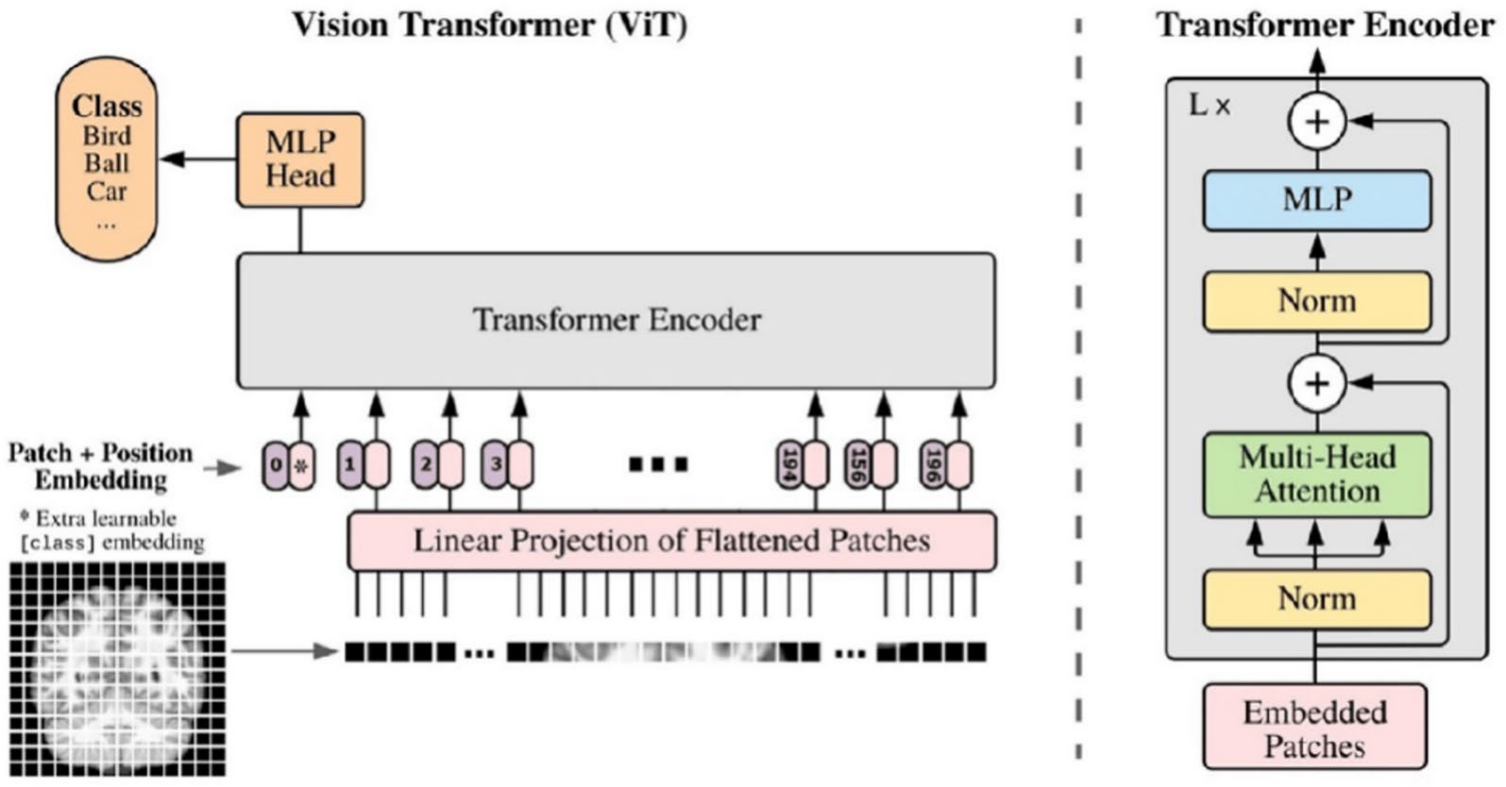
A visual summary of ViTs. ViTs consist of several transformer blocks. Each transformer block comprises two sub-layers, a feed-forward layer and a MHSA layer. Figure adapted from [134]

The ViT adapts the Transformer encoder for image classification by treating an image as a sequence of patches [28]. This reframing of a spatial problem into a sequential one allows the direct application of self-attention to visual data (Fig. 11).

**Image patching:** An input image $x \in \mathbb {R}^{H \times W \times C}$ is divided into a grid of *N* non-overlapping square patches $x_p \in \mathbb {R}^{N \times (P^2 \cdot C)}$, where (*P*, *P*) is the patch resolution and $N = HW / P^2$ is the number of patches.

**Patch embedding:** Each flattened patch vector $x_{p_i}$ is linearly projected into a *D*-dimensional embedding space using a learned weight matrix $E \in \mathbb {R}^{(P^2 \cdot C) \times D}$:

16 $$\begin{aligned} \hat{x}_i = x_{p_i} E \quad \text {for } i=1...N \end{aligned}$$

A learnable class token ($x_{class}$) embedding is typically prepended to the sequence of embedded patches ($\hat{x} = [\hat{x}_1;...; \hat{x}_N]$). This token’s final output state is used to represent the entire image for the classification task [28].

**Positional embeddings:** To retain spatial information, which would otherwise be lost in the tokenization process, learnable 1D positional embeddings $E_{pos} \in \mathbb {R}^{(N+1) \times D}$ are added element-wise to the patch embeddings:

17 $$\begin{aligned} z_0 = [x_{\text {class}}; \hat{x}] + E_{\text {pos}} \end{aligned}$$

This allows the model to learn the relative positions of the patches.

**Transformer encoder processing:** This sequence of $N+1$ vectors ($z_0$) serves as input to a standard Transformer encoder comprising *L* layers. Each layer *l* applies Multi-Head Self-Attention (MHSA) and a Multilayer Perceptron (MLP), incorporating Layer Normalization (LN) [132] and residual connections [133]:

18 $$\begin{aligned} z'_l= & \text {MSA}(\text {LN}(z_{l-1})) + z_{l-1} \end{aligned}$$

19 $$\begin{aligned} z_l= & \text {MLP}(\text {LN}(z'_l)) + z'_l \end{aligned}$$

**Classification head:** The final output state corresponding to the class token after the last layer, $z_L^0$, is typically passed through Layer Normalization and then a classification head (often a simple MLP with one hidden layer) to produce the final prediction *Y*:

20 $$\begin{aligned} Y = \text {ClassificationHead}(\text {LN}(z_L^0)) \end{aligned}$$

While powerful at modeling global context, ViTs often require substantial pre-training on large datasets (e.g., ImageNet-21k, JFT-300 M) due to their limited inherent inductive bias compared to CNNs. This has led to the development of data-efficient training strategies and architectures like DeiT (Data-efficient image Transformers) [135].

#### Convolutional vision transformer: a synthesis of architectures

While ViTs excel at modeling global relationships, their effectiveness can be constrained by a lack of strong inductive biases, making them data-hungry and sometimes less efficient at learning the fine-grained local features critical for medical imaging [136]. To address this, hybrid architectures, known as CViTs, have emerged as a dominant paradigm [33].

A CViT architecture signifies a deliberate fusion of two paradigms: the CNN component for its inherent proficiency in processing local spatial information, and the transformer component for modeling global context [137, 138]. CNNs use stacked convolutional layers to build a hierarchical representation of features, adeptly capturing details like texture and edges. In neuroimaging, this corresponds to identifying subtle textural changes or delineating atrophied structures. Conversely, the transformer’s self-attention mechanism can assess the relationship between any two regions, regardless of distance, which is crucial for understanding the distributed nature of AD pathology. Therefore, a CViT is designed to achieve a more comprehensive representation by integrating these complementary strengths [139].

**Fig. 12:**
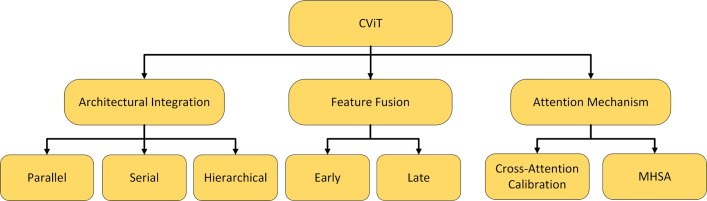
Categorization of CViT architectures

The design of these hybrid architectures can be classified according to their integration strategy, providing a framework for understanding the diverse CViT models in AD research. The primary integration strategies can be organized into three main categories: **Architectural Integration**, **Feature Fusion Strategies**, and **Attention Mechanism Integration** (Fig. 12).

**Fig. 13:**
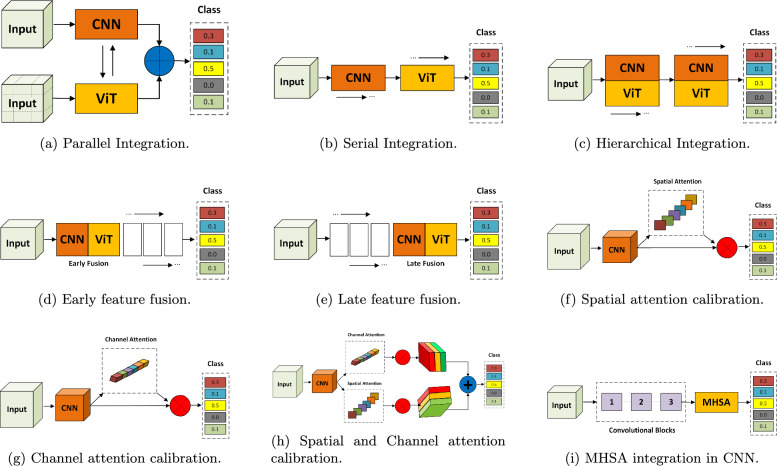
Types of CViT architectures

***Architectural integration patterns***

This category describes the fundamental backbone structure of the hybrid model, focusing on how the CNN and transformer components are arranged.

- **Parallel integration:** In a parallel integration architecture, CNN and ViT branches operate concurrently on the input data, as depicted in Fig. 13a. Each branch extracts features independently, and these distinct feature sets, $F_{cnn} = \phi _{cnn}(X)$ and $F_{vit} = \psi _{vit}(X)$, are then merged using a fusion module, $f_{fuse}$:
  21 $$\begin{aligned} F_{fused} = f_{fuse}(F_{cnn}, F_{vit}) \end{aligned}$$
  This design is effective for both unimodal and multimodal tasks and is exemplified in studies like [140–142].
- **Serial integration:** This strategy follows a sequential "CNN ->Transformer" pipeline, where the CNN acts as a powerful feature extraction front-end (see Fig. 13b). The initial convolutional layers process the input image to learn a rich set of localized, low-level feature maps. These maps are then "tokenized" and fed into the subsequent transformer encoder. Let an input image be denoted as $X \in \mathbb {R}^{H \times W \times C}$. The CNN front-end, $\phi _{cnn}$, extracts a feature map $F_{cnn} = \phi _{cnn}(X) \in \mathbb {R}^{H' \times W' \times D}$. This map is then reshaped into a sequence of *N* tokens, $Z_0 = [t_1, t_2,..., t_N]$, which is processed by the transformer encoder, $\psi _{vit}$:
  22 $$\begin{aligned} Y_{out} = \psi _{vit}(Z_0) \end{aligned}$$
  This is the most common pattern observed in the reviewed literature, seen in models like DAFN [143] and numerous others that use a CNN-based stem [31, 81, 102, 144].
- **Hierarchical integration:** This represents the deepest level of synergy, where convolutional operations are interleaved with self-attention mechanisms within the transformer blocks themselves (see Fig. 13c). This design infuses the transformer with CNN-like properties at multiple scales and is demonstrated in advanced models that alternate specialized convolution and attention modules [30, 32, 145–147].

***Feature fusion strategies***

This classification focuses on *when* in the processing pipeline the information from convolutional and transformer elements is combined.

- **Early fusion:** Fusion occurs at the initial stages of the network (Fig. 13d). This can include signal-level fusion, where different imaging modalities are merged at the pixel level before being fed into the main architecture, as demonstrated in the frameworks of [148–151].
- **Late fusion:** This involves merging features at deeper layers or at the decision level (Fig. 13e). An example is the ensemble model by [152], which uses voting on the final outputs of multiple models.

***Attention mechanism integration***

This category describes hybrids where attention mechanisms are strategically integrated into a predominantly CNN-based architecture.

- **Spatial and channel attention:** These mechanisms help a CNN focus on *where* (spatial) and *what* (channel) information is most important.
  - **Spatial attention:** This mechanism dynamically computes a 2D attention map ($M_s$) to highlight salient spatial regions, as illustrated in Fig. 13f. The refined output $F'$ is obtained by re-weighting the input feature map *F*: $F' = M_s \otimes F$. This principle is applied in the DAFN model to guide the network’s focus [143].
  - **Channel attention:** This mechanism models interdependencies across channels, learning a 1D attention vector ($M_c$) to amplify informative feature channels (see Fig. 13g). The CrossViT architecture reviewed uses channel attention to refine its features [153].
  - **Combined channel and spatial attention:** To achieve a more comprehensive feature refinement, some models integrate both mechanisms (see Fig. 13h). The HAMMF model is an example from our reviewed studies that effectively utilizes this principle within its Contextual Hierarchical Attention Module (CHAM) [154].
- **Cross-attention:** This powerful mechanism facilitates direct information exchange between two distinct sets of features, such as those from different modalities. Instead of performing self-attention, query vectors (*Q*) from one modality’s features attend to the key (*K*) and value (*V*) vectors from another modality’s features:
  23 $$\begin{aligned} \text {Attention}(Q_{A}, K_{B}, V_{B}) = \text {softmax}\left( \frac{Q_{A} K_{B}^T}{\sqrt{d_k}}\right) V_{B} \end{aligned}$$
  This technique is fundamental to many advanced multimodal models in our review, including MCAD [155], 3MT [50], and CsAGP [156].
- **MHSA integration:** This strategy involves directly replacing convolutional layers within a CNN, typically in deeper stages, with a MHSA block, as conceptually illustrated in Fig. 13(i). This architectural shift endows a CNN with the capacity for long-range dependency modeling while retaining the efficiency of convolutions for early-stage feature extraction. The RepBoTNet-CESA model by Zhang et al. [157] is a direct application of this principle in our review.

***Transformer-based architectures demonstrate superiority over traditional CNNs***

The widespread adoption of transformer-based models in AD research stems directly from the inherent limitations of their architectural predecessors, particularly CNNs. The primary constraint of CNNs is their reliance on local receptive fields. Modeling the relationship between distant brain regions–a hallmark of the diffuse, network-level pathology of AD–requires stacking many convolutional layers, an inefficient process for capturing global signatures of neurodegeneration. This architectural gap created a clear motivation for the adoption of ViTs. By employing a self-attention mechanism, ViTs can directly model the relationship between any two image patches, regardless of their spatial distance, offering a more powerful method for capturing the global patterns critical for AD diagnosis.

A central finding of this systematic review is that this theoretical advantage translates into tangible, and often statistically significant, performance gains. Multiple head-to-head comparisons reported in the analyzed literature confirm this trend. For instance, in a direct benchmark, Carcagn et al. [158] revealed that a ViT could "significantly outperform" several modern CNN architectures on AD classification tasks. This pattern of superiority is further reinforced in the CViT literature, where frameworks from Khatri and Kwon [30] and Hosny et al. [159] were explicitly benchmarked to demonstrate higher accuracy than established CNN baselines. However, the flexibility of pure ViTs comes at a cost: they are notoriously "data-hungry" and computationally intensive. It is precisely this trade-off–the powerful global context modeling of transformers versus the efficient local feature extraction of CNNs–that spurred the development of hybrid CViTs, which aim to synthesize the strengths of both paradigms and represent the field’s current state-of-the-art.

***A critical comparison of ViT and CViT performance in AD research***

While both transformer variants show advantages over CNNs, a critical comparison between them reveals a clear and pragmatic trend within the field towards the adoption of hybrid CViTs. The principal strength of a pure ViT lies in its architectural simplicity for modeling global, long-range dependencies. However, as evidenced by the studies reviewed, this comes at a significant cost. Lacking strong local inductive biases, pure ViTs are notoriously data-hungry and computationally expensive, often struggling to learn fine-grained neuropathological features from limited medical imaging datasets [136]. Consequently, their application is often constrained to less complex, single-modality tasks.

In contrast, the overwhelming trend across the 68 studies analyzed in this review points to the emergence of CViTs as a more robust, versatile, and ultimately superior paradigm for AD research. The critical strength of the CViT lies in its synergistic design, which directly mitigates the primary weakness of the pure ViT. By leveraging a CNN front-end for efficient local feature extraction, the CViT provides its transformer backbone with a high-quality, spatially-rich set of tokens. As our review demonstrates, this makes the CViT architecture exceptionally well-suited for the field’s most complex challenges, evidenced by its clear preference in the majority of recent, high-impact multimodal studies [50, 102, 155]. While the relative weakness of CViTs lies in their increased architectural complexity, the empirical evidence is compelling: their superior performance and wider adoption in complex settings confirm them as the current de facto state-of-the-art for tackling the multifaceted diagnostic challenges of AD.

## Single-modality approaches in Alzheimer’s disease

While the integration of multiple data streams represents a frontier in AD research, single-modality analysis remains a cornerstone of both clinical practice and scientific investigation. Before examining the inherent limitations of a unimodal perspective, it is crucial to understand the practical and methodological justifications for its continued use. The decision to employ a single data source is often not a sign of methodological simplicity, but rather a deliberate choice driven by specific clinical, economic, or scientific objectives.

### The rationale and clinical utility of single-modality analysis

Several key scenarios underscore the value and necessity of single-modality approaches in the AD landscape:

- *Clinical pragmatism and accessibility:* In real-world clinical settings, multimodal data acquisition is not always feasible. sMRI is a widely available, non-invasive, and relatively low-cost imaging technique, making it a first-line diagnostic tool in most healthcare systems [10]. Therefore, developing robust models that can extract maximum diagnostic value from this single, commonly available modality (e.g., [160, 161]) is of immense practical importance, particularly for widespread screening and diagnosis in resource-constrained environments.
- *Focused biomarker assessment and research:* Hypothesis-driven scientific inquiry often requires isolating the effect of a specific biological process. A model trained exclusively on amyloid-PET data (e.g., [162]) can serve as a powerful tool to specifically quantify the cerebral amyloid burden [82] or to track target engagement in a clinical trial for an anti-amyloid therapy [12]. In such cases, introducing additional modalities could introduce confounding variables, making it more difficult to assess the specific contribution of the biomarker of interest. Single-modality models are thus essential for building a deep, mechanistic understanding of individual components of AD pathophysiology.
- *Establishing methodological baselines:* From a ML perspective, single-modality models provide an indispensable performance baseline. The claim that a multimodal fusion approach is superior can only be substantiated if it demonstrates a statistically significant improvement over a well-optimized, state-of-the-art single-modality model (e.g., [163]). Without these foundational baselines, the incremental benefit of more complex, computationally expensive multimodal architectures remains unproven [51]. These models therefore serve as the essential “control group” in the development and validation of new computational techniques.

### The inherent limitations of a unimodal perspective

While these justifications underscore the continued relevance of unimodal approaches, the multifaceted pathophysiology of AD inherently limits the diagnostic ceiling of any single perspective. Relying on one data source provides an incomplete snapshot of a complex and progressive disease. Each modality, when analyzed in isolation, offers a view of the disease process that is constrained by its particular sensitivity and specificity, often leading to diagnostic ambiguity, particularly in the early or atypical stages of AD.

The distinct limitations of each primary modality illustrate this challenge:

- *sMRI*, while excellent for detailing brain anatomy, primarily detects macroscopic atrophy [11, 73]. Such volumetric loss is often a lagging indicator of the disease, becoming most apparent after significant and irreversible neurodegeneration has already occurred [4, 164]. Furthermore, patterns of atrophy are not exclusively specific to AD; similar changes can be observed in other neurodegenerative conditions like frontotemporal dementia or vascular dementia, as well as in normal aging, thus limiting its diagnostic specificity.
- *FDG-PET*, which measures cerebral glucose metabolism, can detect neuronal dysfunction before substantial atrophy is visible. However, despite its utility in modern DL frameworks (e.g., [165, 166]), the observed patterns of hypometabolism can also be confounded by other conditions affecting brain function, such as Lewy body dementia or severe depression, again posing a challenge to differential diagnosis [86].
- *Molecular PET* (e.g., Amyloid-PET or Tau-PET) provides high specificity for the core proteinopathies of AD. Yet, the presence of amyloid plaques, for instance, is not a perfect proxy for clinical dementia; a significant portion of cognitively unimpaired older adults exhibit substantial amyloid burden. Amyloid levels tend to plateau early in the clinical course of the disease and correlate poorly with the severity of cognitive decline, limiting their prognostic value for individual patients [17, 84].
- *Fluid biomarkers* from CSF or blood offer direct, quantitative measures of AD pathology (A$\beta$42, p-tau). However, their acquisition can be invasive (in the case of CSF), and they provide a global, non-localized measure of the brain’s biochemical state. They can confirm the presence of AD pathology but lack the spatial resolution to map its topographical distribution or to quantify its downstream consequences on brain structure and function [15].
- *Electrophysiological measures*, such as electroencephalography (EEG), reflect neural activity and have been explored for diagnostic utility [167]. However, they suffer from a low spatial resolution and are highly susceptible to noise and artifacts, making it challenging to pinpoint the precise anatomical sources of abnormal activity related to AD pathology.
- *Genetic markers* like the APOE $\varepsilon$4 allele are powerful for assessing risk [104], and large-scale Genome-Wide Association Studies (GWAS) have identified numerous other risk variants [106]. However, these are not deterministic; they represent a static risk factor rather than a dynamic measure of active disease, providing probabilistic information that is insufficient for a definitive diagnosis or for tracking disease progression [168].

Collectively, these individual constraints underscore a fundamental principle: no single modality can fully capture the complex interplay between molecular pathology, structural degradation, functional disruption, and clinical manifestation that defines the AD continuum. The recognized limitations of relying on any one of these valuable but incomplete data sources provide the critical impetus for the development and application of multimodal data fusion techniques, which aim to synthesize these complementary information streams into a more comprehensive and accurate diagnostic model [17, 46].

## Multimodal data fusion in Alzheimer’s disease

The complex, multifactorial nature of AD, characterized by a cascade of structural, functional, molecular, and genetic alterations, necessitates a departure from unimodal analytical approaches [46, 52]. Relying solely on a single data source—be it neuroimaging (sMRI, PET, DTI, fMRI), CSF analysis, genetic markers, or clinical assessments—provides an inherently incomplete perspective on an individual’s disease status and trajectory [16, 17, 42]. Each modality captures distinct, often complementary, facets of AD pathophysiology; for instance, sMRI delineates patterns of brain atrophy (visible in Fig. 4), PET can reveal metabolic dysfunction or specific proteinopathies (amyloid-beta as in Fig. 6, tau), and CSF analysis offers direct biochemical evidence of these pathological proteins [102, 148, 169]. The inherent limitations of any single modality in comprehensively characterizing AD underscore the critical need for robust multimodal data fusion strategies [170].

Multimodal data fusion, in the context of AD research, refers to the principled and synergistic integration of information from two or more distinct data modalities [47, 171]. The objective is to construct a more holistic, accurate, and robust model of the disease process than could be achieved by analyzing any individual modality in isolation [16, 49, 52]. By effectively combining diverse biomarkers and clinical indicators, advanced fusion techniques aim to enhance the detection of subtle early-stage changes, improve differential diagnosis from other neurodegenerative conditions, and increase the precision of prognostic models, particularly for predicting MCI progression [156, 172, 173]. Despite extensive research demonstrating the benefits of such integrative approaches [49, 50, 62, 172], the identification of a universally optimal fusion strategy remains an ongoing challenge, with the most suitable method often contingent upon the specific research question, the involved modalities, and the dataset characteristics.

### Levels and types of data fusion

Data fusion techniques can be systematically categorized based on the stage within the analytical pipeline at which information integration occurs, a taxonomy that is foundational to the field [174, 175]. This hierarchical perspective distinguishes between three primary fusion levels: Input-Level (Early), Intermediate (Feature-Level), and Output-Level (Late) [47]. Furthermore, fusion methods can be guided by principles, such as being knowledge-based, which can be applied at any level [16, 52, 173, 176]. A model is considered to use Hybrid Fusion when it explicitly combines strategies from more than one of these distinct fusion levels (e.g., Input-Level + Intermediate) or when it integrates fundamentally different and complex methodologies, such as combining generative models with discriminative classifiers [177] or graph-based learning with traditional feature fusion [109].

**Fig. 14:**
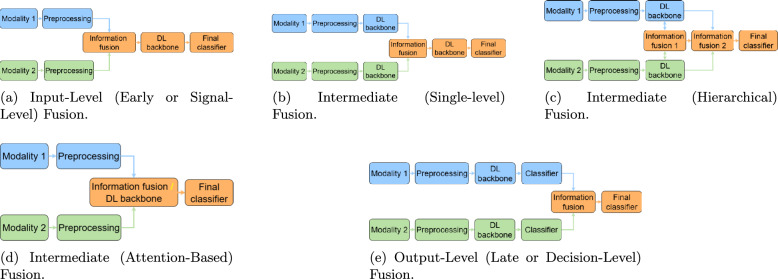
Levels and types of fusion mechanisms

#### Input-level (Early or signal-level) fusion

This strategy, also referred to as data-level or signal-level fusion, involves the integration of raw or minimally processed data from different modalities at the earliest possible stage, typically before any significant feature extraction by a DL backbone (see Fig. 14a) [47]. For neuroimaging data, this often entails merging pixel or voxel information from co-registered scans (e.g., sMRI and PET) to generate a composite, richer input representation. The work by Odusami et al. is a prime example of this, using techniques like DWT and transposed convolutions to merge images before classification [148–151]. Another example is the approach by Kadri et al., where multimodal data is fused prior to being fed into the main classification architecture [178].

Mathematically, let’s consider two perfectly co-registered imaging modalities, an sMRI scan $I_{MRI} \in \mathbb {R}^{H \times W \times D}$ and a PET scan $I_{PET} \in \mathbb {R}^{H \times W \times D}$, where *H*, *W*, *D* are the height, width, and depth dimensions. The simplest fusion method is **channel concatenation**, where the modalities are stacked to form a multi-channel input tensor $I_{fused}$:

24 $$\begin{aligned} I_{\text {fused}} = \text {Concat}(I_{MRI}, I_{PET}) \in \mathbb {R}^{H \times W \times D \times 2} \end{aligned}$$

Alternatively, a **weighted average** can be computed at each voxel (*i*, *j*, *k*):

25 $$\begin{aligned} I_{\text {fused}}(i,j,k) = w_{MRI} \cdot I_{MRI}(i,j,k) + w_{PET} \cdot I_{PET}(i,j,k) \end{aligned}$$

Here, $w_{MRI}$ and $w_{PET}$ are scalar weights, often satisfying $w_{MRI} + w_{PET} = 1$, which can be fixed or learned. More advanced techniques like the Discrete Wavelet Transform (DWT), as explored by Odusami et al. [148], decompose each image into approximation and detail coefficients, which are then fused using specific rules before an inverse transform reconstructs the fused image.

The primary advantage of input-level fusion is the potential preservation of all original information from each modality, allowing the subsequent DL model to learn inter-modal relationships from the ground up. However, this approach is highly sensitive to noise inherent in raw data and critically depends on meticulous spatial alignment (co-registration) between modalities [179]. Misregistration artifacts can propagate and severely degrade the performance of the fused model. Furthermore, combining raw data from highly disparate sources (e.g., imaging and non-imaging tabular data) at this level can be challenging due to differences in data structure and scale [180].

**Spatial fusion**, as a specific subtype of input-level fusion primarily applied to imaging data, focuses on combining information at the pixel or voxel level to enhance spatial resolution, improve signal-to-noise ratio, or create a more comprehensive spatial representation. Techniques often involve sophisticated image registration followed by methods like Gaussian/Laplacian Pyramid (GLP) decomposition and Spatial Pulsed Neural Coupling Networks (SPCNN) to merge sMRI and PET data, as demonstrated by Odusami et al. [151]. While sharing the "early" characteristic of signal-level fusion, spatial fusion often implies more advanced, spatially-aware combination techniques.

#### Intermediate (Feature-level) fusion

Intermediate, or feature-level, fusion is arguably the most prevalent and versatile strategy in AD research employing ML and DL [46, 52]. In this paradigm, relevant features are first extracted independently from each modality using dedicated encoders (e.g., CNNs for imaging data, MLPs for tabular data). These modality-specific feature sets are then combined to form a unified, often lower-dimensional, representation that is subsequently fed into a classifier or predictor module [181, 182].

Let’s assume we have *N* modalities. For each modality $m_i$, a dedicated feature extractor function $\phi _i$ (e.g., a CNN for an image or an MLP for tabular data) produces a feature vector $f_i \in \mathbb {R}^{d_i}$.

26 $$\begin{aligned} f_i = \phi _i(m_i) \end{aligned}$$

The fusion process combines these *N* feature vectors into a single representation $f_{\text {fused}}$. The most common method is concatenation:

27 $$\begin{aligned} f_{\text {fused}} = [f_1; f_2; \dots ; f_N] \in \mathbb {R}^{\sum d_i} \end{aligned}$$

This concatenated vector $f_{\text {fused}}$ is then used for the final prediction. Other methods include element-wise operations like summation or multiplication if the feature vectors share the same dimension ($d_i=d$).

This approach offers considerable flexibility, allowing for the integration of highly heterogeneous data types. Modern DL architectures, particularly transformers and attention-based models, excel at processing these combined feature vectors, learning complex dependencies and interactions across modalities. Within intermediate fusion, several specific architectures can be identified:

- **Single-level fusion (Classic and network fusion) Fig.** 14b: This involves a single stage of feature fusion. *Classic Fusion* concatenates or merges high-level features extracted by separate DL backbones before classification. *Network Fusion* might involve fusing intermediate-level features and then passing them through additional shared DL layers for further abstraction prior to classification. Numerous reviewed studies exemplify this approach, particularly those integrating imaging with clinical data [49, 102, 142, 169, 183, 184]. The DE-JANet model [102] is a quintessential example of single-level network fusion.
- **Hierarchical fusion Fig.** 14c: This involves multiple stages of feature fusion at different levels of abstraction within the network, allowing for the learning of increasingly complex feature combinations. This strategy is clearly demonstrated in the MMTFN framework by Miao et al. [147], the Dual-3DM^3^-AD model by Khan et al. [182], and the hierarchical frameworks of [181] and [62].
- **Attention-based fusion Fig.** 14d: This employs attention mechanisms to dynamically weigh the importance of different features and modalities. Let the concatenated features be represented as a sequence of tokens $F = [f_1, f_2, \dots , f_N]$. A self-attention mechanism [27] can be applied to learn a contextually-aware fused representation:
  28 $$\begin{aligned} \text {Attention}(Q, K, V) = \text {softmax}\left( \frac{QK^T}{\sqrt{d_k}}\right) V \end{aligned}$$
  where the Query (*Q*), Key (*K*), and Value (*V*) matrices are derived from *F*. Alternatively, **cross-attention** is used to fuse two feature sets, $f_A$ and $f_B$. Here, one modality’s feature acts as the query, attending to the other:
  29 $$\begin{aligned} f'_{A} = \text {CrossAttention}(Q=f_A, K=f_B, V=f_B) \end{aligned}$$
  This process is central to models that explicitly use cross-modal attention, such as MCAD [155] and CsAGP [156]. The AD-Transformer by Yu et al. [49] also uses self-attention on a unified sequence of tokens from sMRI, clinical, and genetic data.

While powerful, simple concatenation in intermediate fusion can lead to very high-dimensional and often redundant feature spaces—a phenomenon known as the ’curse of dimensionality’ [185]—that can degrade model performance by introducing noise and complicating the learning process [186]. Without sophisticated subsequent processing like attention or dimensionality reduction, as explored by Sait and Nagaraj [187], the model may not optimally balance contributions from different modalities. Careful feature engineering, selection, and the design of the fusion mechanism are critical for success. **Temporal Fusion**, crucial for longitudinal AD studies, often falls under intermediate fusion. It involves combining information (typically extracted features) from multiple time points for the same subject to model disease progression or stability. The VGG-TSwinformer by Hu et al. [131] is a prime example of this "Intermediate (Temporal)" approach, using a transformer to fuse features from sMRI at two time points.

#### Output-level (Late or decision-level) fusion

In output-level fusion, also known as decision-level or late fusion, separate predictive models are trained independently for each modality or subset of modalities (see Fig. 14e) [47]. The outputs from these individual models (e.g., class probabilities, regression values, or discrete decisions) are then combined at the final stage to arrive at a consensus prediction. The work by Kadri et al. [188], which explicitly tested and compared the performance of late fusion against other strategies as part of a broader study, serves as a clear example of this approach in the reviewed literature.

Let’s say we have *N* models, where each model $M_i$ produces a probability distribution vector $p_i \in [0, 1]^C$ for *C* classes. The final fused prediction $p_{\text {fused}}$ can be obtained through several aggregation rules. A common method is averaging:

30 $$\begin{aligned} p_{\text {fused}} = \frac{1}{N} \sum _{i=1}^{N} p_i \end{aligned}$$

Alternatively, a **weighted average** can be used, where each model’s prediction is weighted by its confidence or predetermined importance, $\alpha _i$:

31 $$\begin{aligned} p_{\text {fused}} = \sum _{i=1}^{N} \alpha _i p_i \quad \text {where} \quad \sum _{i=1}^{N} \alpha _i = 1 \end{aligned}$$

In **majority voting**, the class that receives the most votes (highest probability) across the individual models is chosen as the final prediction. In **stacking**, the prediction vectors $[p_1, p_2, \dots , p_N]$ are concatenated and used as input to a final "meta-learner" model, $M_{\text {meta}}$ [189]:

32 $$\begin{aligned} p_{\text {fused}} = M_{\text {meta}}([p_1; p_2; \dots ; p_N]) \end{aligned}$$

This approach offers excellent modularity, allowing for the development and optimization of specialized models tailored to the characteristics of each data type. It can also be inherently robust to noise or missing data in a particular modality, as the final decision is not solely reliant on any single input stream. However, a potential drawback is that valuable correlation information and subtle inter-modal dependencies that could have been captured at the intermediate level might be discarded. The overall performance of late fusion heavily depends on the accuracy and diversity of the individual unimodal predictors and the effectiveness of the chosen combination rule [190].

#### Knowledge-based and hybrid fusion strategies

**Knowledge-based fusion**, as defined in this review, is not a fusion level but rather a guiding principle that can be incorporated at any stage. It involves integrating domain-specific prior knowledge into the fusion process to enhance model performance or interpretability [52]. This is exemplified in a wide range of studies that leverage different forms of knowledge:

- *Clinical and demographic data:* Many models directly integrate features like age, cognitive scores (MMSE, ADAS-Cog), and education to provide clinical context to imaging data [49, 143, 183, 184, 191].
- *Biochemical and genetic knowledge:* Some architectures fuse imaging with direct pathological evidence from CSF biomarkers [155] or use genetic information (like APOE status) to inform the model [49, 192].
- *Anatomical knowledge:* Other models incorporate prior anatomical knowledge by using brain atlases to define Regions of Interest (ROIs), which guides the model to focus on pathologically relevant areas [193], or by building knowledge graphs of brain regions and genes [109].
- *Multi-task learning constraints:* A sophisticated form of knowledge injection involves training the model on auxiliary tasks, such as predicting age or cognitive scores, which forces the model to learn clinically relevant representations, as demonstrated by Liu et al. [154].

Mathematically, this can be represented in several ways. A sophisticated approach is to use the knowledge to **modulate** imaging features, for example, by using age to dynamically compute scaling ($\gamma$) and shifting ($\beta$) parameters:

33 $$\begin{aligned} f'_{\text {img}} = \gamma (f_{\text {know}}) \odot f_{\text {img}} + \beta (f_{\text {know}}) \end{aligned}$$

Here, $\odot$ represents element-wise multiplication, and $\gamma (\cdot )$ and $\beta (\cdot )$ are small neural networks that learn to control the influence of the image features based on the provided knowledge. This allows the model to, for example, interpret brain atrophy patterns differently for an 85-year-old versus a 65-year-old, as seen in the work of Gao et al. [184]. This approach requires careful validation to ensure that the incorporated knowledge is accurate and does not introduce undue bias.

**Hybrid fusion approaches**, in contrast, are defined as those that explicitly combine strategies from more than one distinct fusion level (i.e., Input, Intermediate, or Output) or that integrate fundamentally different methodologies (e.g., generative and discriminative) into a single, cohesive framework. Based on this broader definition, several reviewed studies are categorized as hybrid. We can formalize these complex systems as follows:

- **Hybrid (Generative) models**: These systems often involve a generator *G* and a classifier *C*. The generator might learn to create a fused or completed data representation from multiple inputs, which is then classified. For modalities $M_A$ and $M_B$, the process can be abstracted as:
  34 $$\begin{aligned} \hat{M}_{\text {fused}} = G(M_A, M_B; \theta _G) \end{aligned}$$
  35 $$\begin{aligned} \text {Prediction} = C(\phi (\hat{M}_{\text {fused}}); \theta _C) \end{aligned}$$
  where $\phi$ is a feature extractor. This approach is seen in the work of Gao et al. [172], Zuo et al. [177, 194], and Liu et al. [192].
- **Hybrid (Graph-based) models**: Here, multimodal features are first structured into a graph $\mathcal {G} = (\mathcal {V}, \mathcal {E})$, where vertices $\mathcal {V}$ represent features (e.g., brain regions, genes) and edges $\mathcal {E}$ represent their relationships. A Graph Transformer, $f_{\text {GNN}}$, then learns on this structure:
  36 $$\begin{aligned} \mathcal {V}_{\text {fused}} = f_{\text {GNN}}(\mathcal {V}, \mathcal {E}) \end{aligned}$$
  This is a hybrid of graph theory and attention mechanisms, as demonstrated by Zou et al. [109].
- **Hybrid (Multi-level system) models**: These systems combine operations at different fusion levels. For instance, a model might have a primary intermediate fusion pathway, $f_{\text {feat}}$, and an auxiliary output-level guidance pathway, $f_{\text {out}}$. The total loss $\mathcal {L}_{\text {total}}$ is a weighted sum:
  37 $$\begin{aligned} \mathcal {L}_{\text {total}} = \mathcal {L}_{\text {primary}}(f_{\text {feat}}(M_A, M_B)) + \lambda \sum _{i} \mathcal {L}_{\text {aux},i}(f_{\text {out},i}(M_A, M_B)) \end{aligned}$$
  This is characteristic of the 3MT model by Liu et al. [50]. The approach of explicitly comparing different fusion levels, as done by Kadri et al. [188], also falls into this category.
- **Hybrid (Systemic) models**: These integrate complex, distinct subsystems. For example, a model might combine a main fusion pipeline $f_{\text {fuse}}$ with an iterative knowledge-guided loop $f_{\text {guide}}$:
  38 $$\begin{aligned} \text {Prediction} = f_{\text {fuse}}(M_A, M_B, f_{\text {guide}}(\text {Knowledge})) \end{aligned}$$
  This is seen in architectures that use a separate, iterative knowledge-guided loop [193] or a multi-task learning framework, as in the HAMMF model [154].

These advanced architectures offer maximum flexibility but demand careful design and intricate schemes for balancing contributions from different stages and methodologies.

Multimodal data fusion is an indispensable component of advanced AD research, providing a pathway to more accurate, reliable, and comprehensive diagnostic and prognostic models. The choice of fusion strategy—spanning input, intermediate, and output levels, and incorporating spatial, temporal, and knowledge-based considerations—is highly context-dependent. While intermediate (feature-level) fusion, particularly when enhanced by attention mechanisms within sophisticated DL frameworks like transformers, has shown considerable promise [49, 50, 155, 156, 172], significant challenges remain. These include managing data heterogeneity, mitigating the curse of dimensionality, addressing computational costs, and the persistent need for large, well-curated, and diverse multimodal datasets. Future research must continue to innovate and rigorously validate robust, interpretable, and scalable fusion techniques tailored to the multifaceted complexities of AD, with the ultimate aim of translating these advanced computational methods into tangible clinical benefits for patients.

**Fig. 15:**
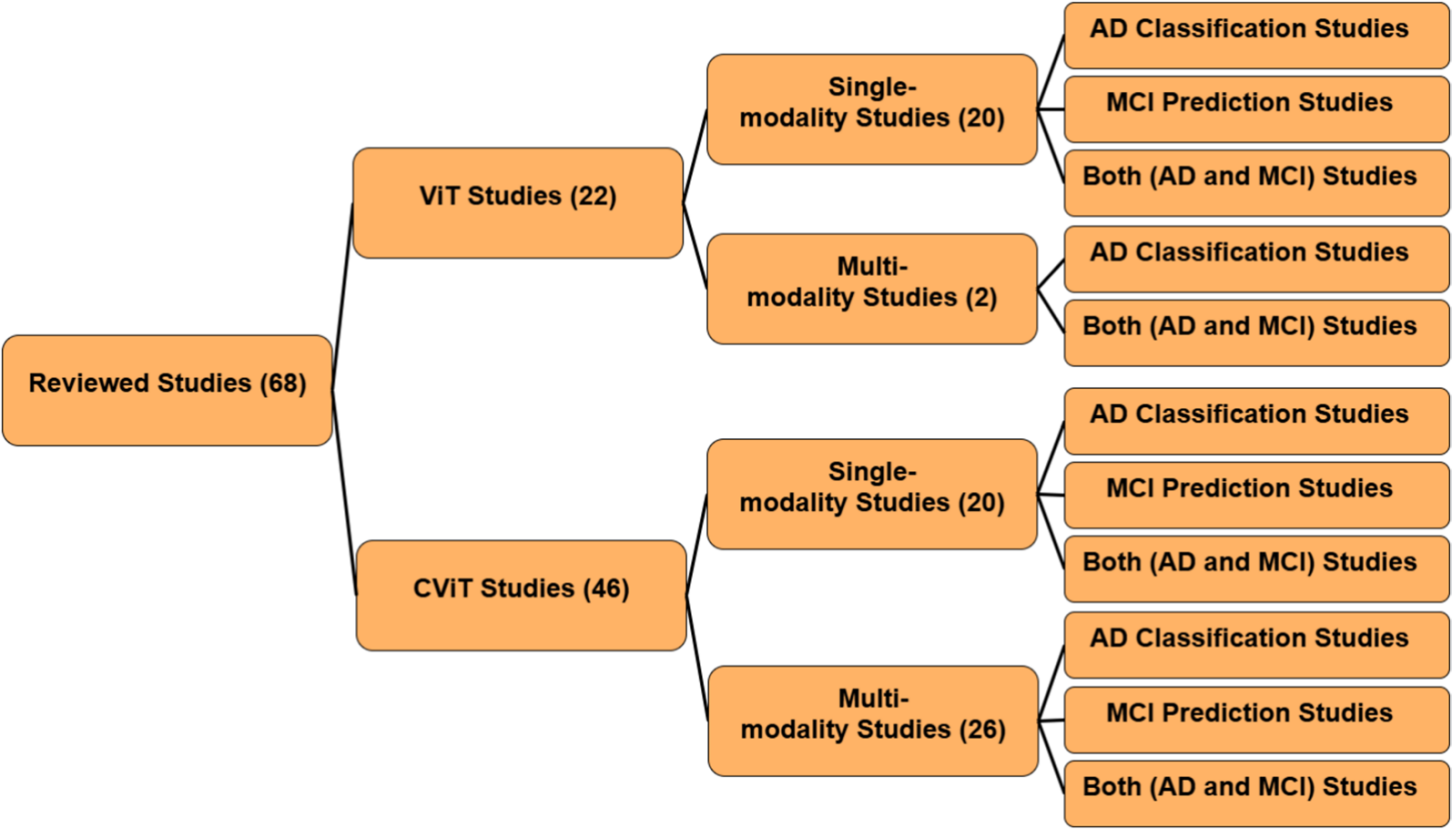
Details of reviewed studies categorization

The subsequent sections present a systematic analysis of 68 contemporary studies examining the application of ViT architectures (Sect. 7) and hybrid CViT models (Sect. 8) in AD research. Collectively, these investigations target two primary diagnostic challenges: the classification of AD stages (e.g., CN, MCI, AD) and the prediction of MCI progression to AD, as taxonomized in Fig. 15.

**Fig. 16:**
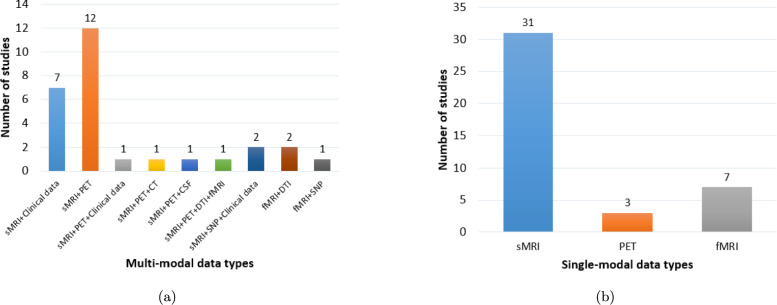
The two bar chart display:** a** medical data types used in multimodal studies,** b** medical data types used in single-modality studies

- Most recent studies use CViT-based models. These combine CNNs for local feature extraction with ViTs for global context modeling. This shows a shift toward hybrid architectures.
- Most work uses single-modality data, mainly sMRI. Fewer studies use multimodal data that combine neuroimaging with clinical, demographic, or genetic information.
- Most models focus on AD stage classification. Fewer address MCI-to-AD progression. This shows that progression prediction is still underexplored, despite its importance for early intervention.
- Figure 16a presents the medical data types used in multimodal studies.
- Figure 16b shows the medical data types used in single-modality studies.

Detailed summaries of these studies, encompassing authorship, publication year, diagnostic task, modality configuration (single modality vs. multimodality), fusion strategies (for multimodal inputs), dataset specifics, technique used, classification categories, validation approaches, and performance metrics (e.g. accuracy, sensitivity, specificity, precision, and AUC) are provided in Table 4 for ViT-based approaches and Table 5 for CViT-based models. This structured synthesis aims to elucidate current trends, identify existing research lacunae, and underscore opportunities for advancing AD diagnostic precision.

## Vision transformers in Alzheimer’s disease domain

Building upon the foundational concepts of the ViT architecture detailed in Subsect. 4.4.1, this section provides a focused examination of the applications based on ViT architectures within AD research. The subsequent discussion reviews 22 studies, as identified in Fig. 15 and summarized in Table 4, that utilize ViTs for AD diagnosis and MCI progression prediction. The section is primarily structured based on the input data modality, first detailing applications with single-modal data (Subsect. 7.1), followed by those employing multimodal data (Subsect. 7.2). A comprehensive analysis, encompassing operational pipelines, observed trends, and key conclusions, is presented in Subsect. 7.3.

### Applications of ViT with single-modality

**Fig. 17:**
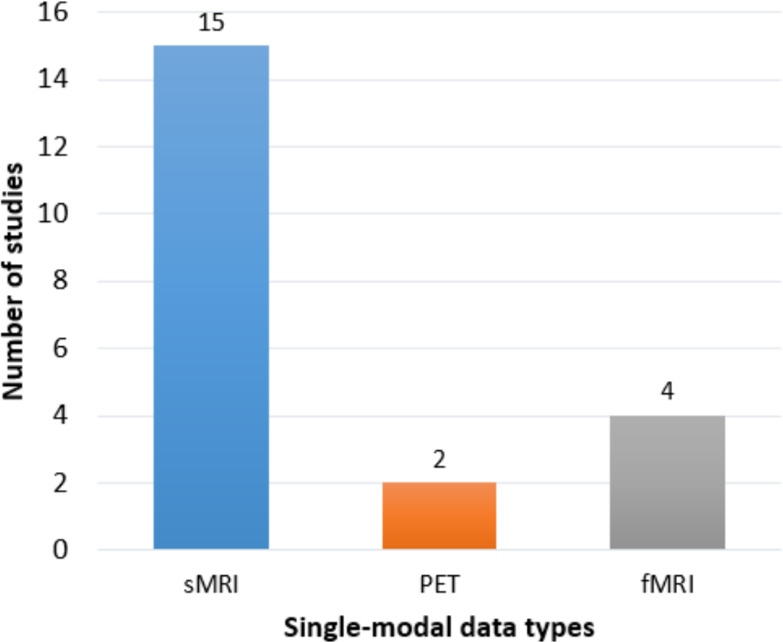
The bar chart displays an overview of medical data types used in single-modality studies using ViT

This subsection delves into ViT-based studies that utilized a single data modality for AD-related diagnostic and prognostic tasks. As categorized in Fig. 15, these investigations were further organized based on their primary objective: AD classification (Subsect. 7.1.1), MCI-to-AD conversion prediction (Subsect. 7.1.2), and studies addressing both objectives concurrently (Subsect. 7.1.3). An overview of the specific imaging modalities employed across these single-modality studies is illustrated in Fig. 17.

#### Single-modality AD classification

This subsection explores research studies that applied standalone ViT architectures to single modality neuroimaging data for AD classification. The subsequent review details various methodological approaches, including those utilizing sMRI, PET, and fMRI, and their reported efficacy in distinguishing different stages of AD or related cognitive decline.

*First: the studies that employ sMRI imaging for the diagnosis of AD:* Mora-Rubio et al. [161] evaluated several DL architectures for the classification of AD stages using 3D MRI. Their study compared SOTA 3D CNNs (EfficientNet and DenseNet), a custom Siamese 3D network, and ViT. The methodology involved image preprocessing using FreeSurfer and spatial data augmentation (rotation, flip, and zoom) on a curated dataset combining images from the Alzheimer’s disease Neuroimaging Initiative (ADNI) and Open Access Series of Imaging Studies (OASIS) databases. However, by merging these cohorts before creating train/test splits, the study’s design precludes a true external validation, a common methodological weakness that may limit the generalizability of the findings. The models were tested on various binary classification tasks (e.g., AD vs CN, LMCI vs CN, MCI vs CN, EMCI vs CN). The ViT achieved the best performance for the AD vs CN task with approximately 89% accuracy. Across the different classification tasks comparing various disease stages, the best-performing models yielded accuracies ranging from around 66% to 89%. These results, while notable, should be interpreted in the context of this lack of external testing and the modest cohort sizes for some of the finer-grained classifications.

Carcagn et al. [158] evaluated the performance of advanced CNNs and ViTs for AD diagnosis using brain MRI data. The study rigorously compared three modern CNN architectures (ResNet, DenseNet, and EfficientNet) and two transformer-based models (Masked Autoencoders - MAE and Data-efficient image Transformers - DeiT). Their methodology utilized a standard preprocessing pipeline including registration and slicing, and commendably implemented a strict subject-level data splitting protocol to prevent the data leakage issues that have compromised the validity of many prior studies. They systematically tested performance using varying numbers of 2D MRI slices (4, 8, and 16) per subject, selected based on entropy, drawn from the ADNI-2 and OASIS-1 datasets for AD vs. NC classification. The experiments revealed that transformer architectures, specifically DeiT, significantly outperformed all tested CNN models, achieving accuracies up to 77% on ADNI-2 and approximately 76% on OASIS-1. While the use of two distinct datasets is a strength, the models were validated only via internal cross-validation within each respective cohort. The study did not perform a cross-dataset external validation (e.g., training on ADNI and testing on OASIS), which remains a critical step for assessing true model generalizability across different clinical populations and imaging protocols. Transformers also demonstrated greater robustness to variations in the number of input slices.

**Fig. 18:**
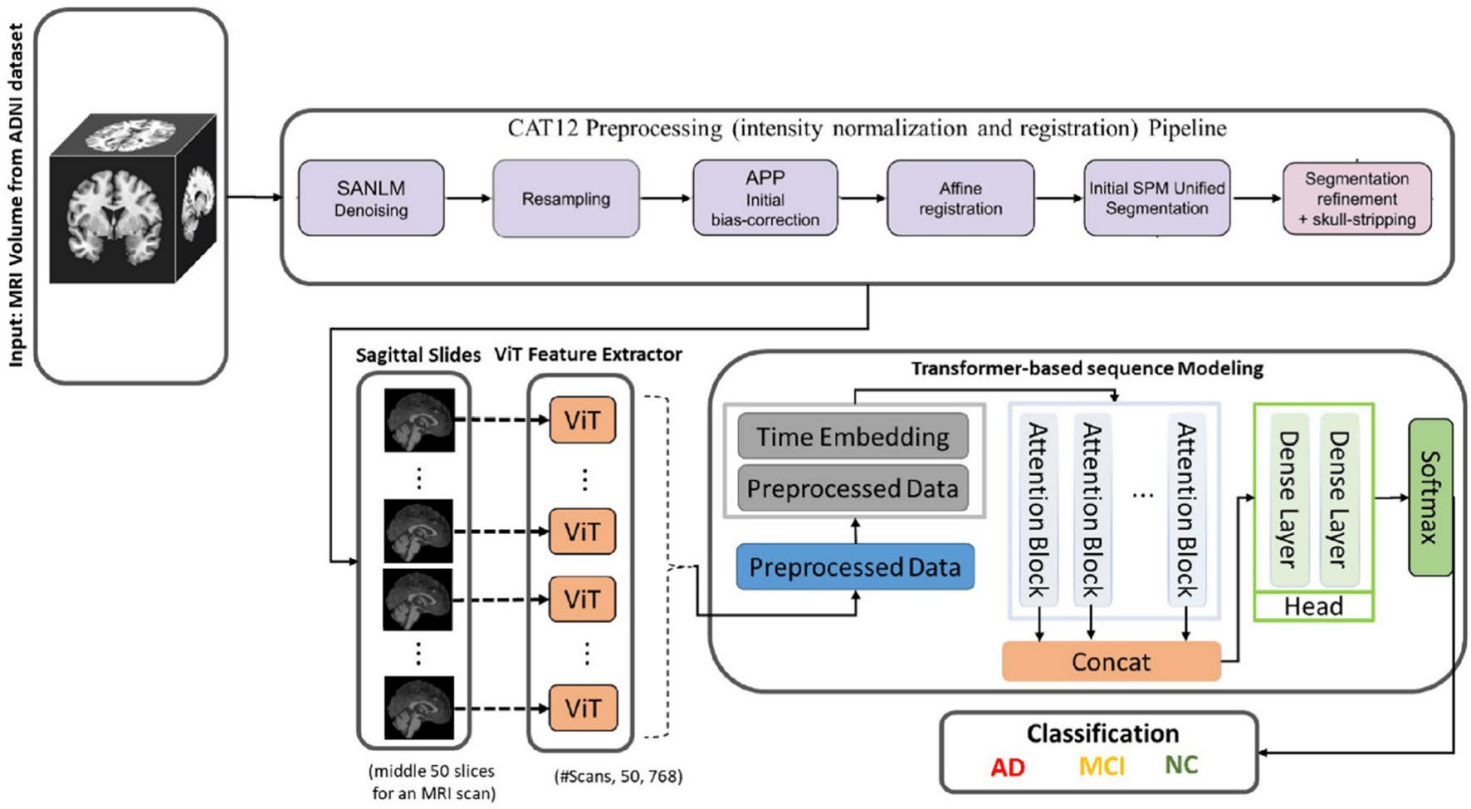
The two-stage pipeline of the ViT-TST model. A ViT extracts features from preprocessed 2D MRI slices, and a subsequent TST classifies the resulting feature sequence. Figure adapted from [134]

Alp et al.  [134] introduced a joint transformer architecture, ViT-TST, for AD classification using 3D sMRI data, focusing on capturing inter-slice dependencies. The methodology (Fig. 18) began with preprocessing T1-weighted ADNI MRI scans using CAT12 (intensity normalization, MNI registration, and skull stripping), followed by converting the 3D volumes into sequences of 2D slices. The ViT-TST model comprised two sequential transformer stages. First, a pre-trained ViT processed each 2D slice independently. The ViT extracted a high-level feature vector from its class token for each slice. Second, the sequence of these slice-derived feature vectors was treated as a time series and input to a Time Series Transformer (TST), which employed MHSA to model long-range dependencies across the ordered slice sequence. The final output from the TST was then passed to an MLP-based classification head for diagnostic prediction (NC, MCI, AD). For evaluation, the authors used a simple random split (60% train, 20% validation, 20% test) of the ADNI datasets. While this approach is common, it lacks the rigor of k-fold cross-validation and, more importantly, does not include any external validation on an independent cohort. This methodological choice raises concerns about potential overfitting and whether the exceptionally high reported accuracies would generalize to more diverse, real-world clinical data. Evaluated on ADNI Complete 1Yr 1.5T and Complete 3Yr 3T datasets, ViT-TST achieved high accuracies (e.g., 99.048% for binary NC vs. AD and 99.014% for multiclass on the 3T dataset), outperforming CNN-BiLSTM and ViT-BiLSTM baselines. This performance highlighted the efficacy of its dual-transformer approach in preserving long-range spatial relationships within MRI volumes for enhanced AD classification.

In the state of the art, Joy et al. [195] introduced ViTAD, a modified ViT model tailored for the multi-stage classification of AD using brain MRI scans. The study leveraged Google’s ViT architecture, enhancing it with fine-tuned hyperparameters and additional layers to improve performance specifically for the 5-class AD stage detection task (CN, EMCI, LMCI, MCI, AD). A comprehensive preprocessing pipeline was employed, including grayscale-to-RGB conversion, image cropping, and Laplacian sharpening for clarity, alongside extensive data augmentation. The ViTAD model was trained and evaluated on a dataset of 1,296 MRI images from the ADNI database. Methodologically, the study relied on a simple 85%/15% train/test split without employing more robust validation techniques like k-fold cross-validation or testing on an external dataset. This approach carries a significant risk of overfitting, especially given the small cohort size for a complex five-class problem. The results were highly impressive, achieving 99.98% accuracy, 100% precision, and a 1.00 F1-score on the test set after only 20 epochs. However, the near-perfect accuracy reported must be interpreted with considerable caution. Such performance, achieved without rigorous external or cross-validation, is often an indicator that the model may not generalize well to new, unseen data from different clinical settings, representing a potential instance of the ’accuracy paradox’ where high performance on a curated test set does not translate to real-world clinical utility.

*The previously discussed studies primarily focused on binary, ternary, quaternary, or, in some cases, five-class classification tasks using sMRI, targeting diagnostic categories such as AD, NC (HC, CN), MCI, LMCI, EMCI, sMCI, and pMCI. In contrast, the following group of studies introduced an alternative classification framework also using sMRI by categorizing the dataset into four distinct stages of cognitive decline: Non-Demented (ND), Very Mild Demented (VMD), Mild Demented (MD), and Moderate Demented (MoD). This shift in classification strategy aimed to provide a more nuanced understanding of disease progression in Alzheimer’s diagnostics.*

Almufareh et al. [196] investigated the use of a ViT approach, leveraging its inherent attention mechanisms, for the detection and multi-class classification of AD using MRI. Their technique involved standard preprocessing of MRI scans followed by classification using a ViT network architecture. The model was trained and evaluated on a publicly available Kaggle dataset (derived from OASIS-1) containing approximately 80,000 MRI images categorized into four AD stages (ND, VMD, MD, and MoD). Methodologically, the study’s reliance on a simple 80/20 train/validation split without k-fold cross-validation or external testing on a separate clinical cohort is a significant limitation. The presented ViT approach demonstrated high performance, achieving an accuracy of 99.06%, precision of 99.06%, recall of 99.14%, and an F1-score of 99.1%. However, such high accuracy should be interpreted with caution, as the less rigorous validation strategy and the use of a pre-processed Kaggle dataset, which may not fully reflect the challenges of raw clinical data, could lead to an overestimation of the model’s performance in a real-world setting.

**Fig. 19:**
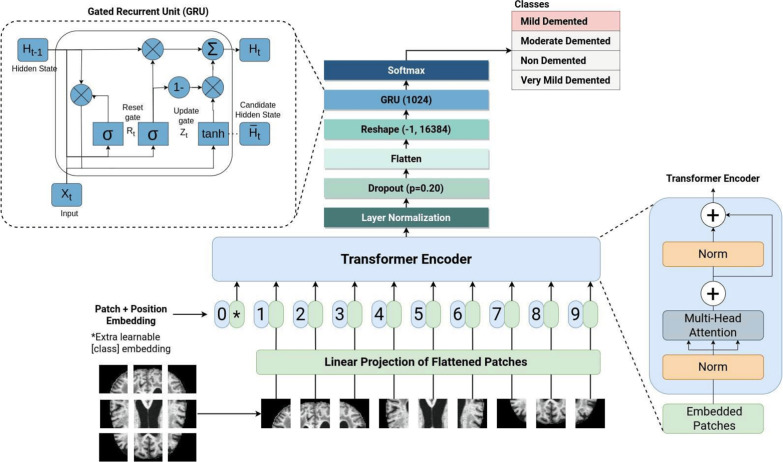
The hybrid ViT-GRU framework. The model synergistically combines ViT for spatial feature extraction with a GRU for sequential feature processing and final classification. Figure adapted from [197]

Mahim et al.  [197] introduced ViT-GRU, a hybrid DL architecture for AD detection and multi-stage classification using MRI data. The model synergized a ViT for spatial feature extraction from 2D MRI slices with a GRU to model sequential dependencies or learn compact representations from these features (Fig. 19). Input MRI scans underwent standard preprocessing, and the resulting patch embeddings from the ViT were processed by a GRU layer before final classification. A significant aspect of this work was the integration of XAI techniques, including LIME, SHAP, and ViT-derived attention maps, to enhance model interpretability by highlighting salient image regions and feature contributions. The model was evaluated on both a Kaggle dataset and the ADNI Baseline dataset using a robust 10-fold cross-validation methodology. While the use of 10-fold cross-validation demonstrates strong internal validity, the study’s evaluation was confined to separate assessments on each dataset. It did not include a cross-dataset external validation, which would be essential to test the model’s generalizability to different data distributions and acquisition protocols. ViT-GRU achieved high mean accuracies, notably 99.53% for the 4-class Kaggle task and 99.26% for the 3-class ADNI task. These near-perfect results, particularly on the pre-processed Kaggle dataset, should be viewed as a strong benchmark of performance under controlled conditions, though further validation on raw, unseen clinical cohorts is necessary to confirm their real-world applicability.

Shah et al. [198] proposed BiViT, a DL architecture for classifying AD stages and other cognitive disorders using 2D MRI. The model leveraged transformer capabilities, using self-attention to capture complex spatial patterns and long-range dependencies. BiViT introduced novel modules, namely Parallel Coupled Encoding Strategy (PCES), which simultaneously processed image patches and tokenized representations, and Mutual Latent Fusion (MLF) to effectively combine the learned features. The model was trained and evaluated on two datasets: a 4-class AD stage dataset derived from Kaggle and a 5-class cognitive disorder dataset derived from ADNI. The study’s validation was based on a simple train/test split for each dataset, without employing more robust methods like k-fold cross-validation or cross-dataset external validation. BiViT achieved high performance on the Kaggle AD dataset, reaching 96.38% accuracy (without augmentation). However, performance decreased significantly to around 45% accuracy on the more complex and imbalanced 5-class ADNI dataset. This stark performance drop between the two cohorts highlights a critical generalizability issue and suggests that the high accuracy on the Kaggle dataset may not be indicative of the model’s performance on more challenging, clinically relevant, and imbalanced data. It underscores the methodological weakness of relying on single, often pre-processed, datasets for validation.

Furthermore, Alshayeji [199] introduced a framework for AD detection and multi-stage classification using ViTs applied to MRI. The study utilized ViT’s self-attention mechanism to capture long-range spatial relationships within brain images, contrasting with the local focus of traditional CNNs. A pre-trained ViT model (vit-base-patch16-224-in21k) was fine-tuned via transfer learning for the task of classifying MRI scans into four stages: ND, VMD, MD, and MoD. The model was trained and evaluated on a publicly available Kaggle dataset containing 6400 MRI images across these four categories. The evaluation was conducted using a simple 85%/15% train/test partition of this single Kaggle cohort. This methodology, lacking both k-fold cross-validation and testing on an independent clinical dataset, presents a significant limitation to the study’s claims of generalizability. The proposed ViT-based approach achieved exceptionally high performance, reporting 99.83% accuracy, 99.69% sensitivity (recall), 99.88% specificity, and 99.54% precision. However, such near-perfect results, derived from a less-than-rigorous validation strategy on a single, pre-processed dataset, should be interpreted with caution, as they may not be reproducible in a more challenging, real-world clinical setting. The study also employed visualizations, including mean attention distances and attention heatmaps, to interpret the model’s learning process.

In another study in the same year, Hosny et al. [159] proposed an XAI framework for automated brain abnormality detection, including AD and tumors, using MRI. The core of the method was the EfficientViT, which was chosen for its efficiency and ability to capture global context compared to CNNs. A robust preprocessing pipeline featuring the AutoCanny algorithm for adaptive edge detection was employed to enhance image quality. EfficientViT’s architecture, with its memory-efficient sandwich layout and cascaded group attention, was trained and evaluated on multiple datasets (AD, Tumor1, Tumor2, and a merged set) using 5-fold cross-validation. While this multi-dataset approach and the use of 5-fold cross-validation represent a methodologically robust form of internal validation, the study stopped short of performing a true external validation, which would involve training on one dataset and testing on a completely separate, unseen one. The model achieved high accuracy, notably 99.24% on the 4-class AD dataset, outperforming several established CNN architectures. These impressive results demonstrate the model’s high performance within the tested cohorts, but its generalizability to clinical data from entirely different sources remains an open question. Explainability was a central theme, addressed using Gradient-based Shapley Additive Explanations (Grad-SHAP) to visualize model predictions and highlight diagnostically relevant features.

Pramanik et al. [200] developed the Fuzzy Granule-based Interpretable Cognitive ViT (FGI-CogViT) for multi-class AD detection using MRI scans. This model addressed limitations of standard ViTs, specifically uncertainty between AD stages, computational cost, and lack of interpretability. FGI-CogViT uniquely combined statistical features (derived from GLCM) and vision features. It employed fuzzy logic-based granulation on statistical features to identify and refine disease-prone regions ("crisp granules"), which handled stage ambiguity. The classification component (I-CogViT) then fused these crisp statistical features (processed via a residual network) with the vision features (processed via a traditional ViT component). The model was evaluated on a 4-class Kaggle MRI dataset (ND, VMD, MD, and MoD). While the architectural approach is novel, the study’s validation was restricted to this single Kaggle dataset without testing on independent clinical cohorts such as ADNI or OASIS. This reliance on a single, pre-processed data source is a significant limitation. The framework demonstrated high performance, achieving 98.83% accuracy and a 99.47% F1-score, outperforming several comparative models. However, these impressive results should be considered preliminary, as the model’s ability to generalize to more heterogeneous, raw clinical data from different scanners and patient populations remains unverified.

In 2025, Kurniasari et al. [201] investigated the application of the ViT model for multi-class AD diagnosis using MRI scan images. Their study focused on enhancing classification accuracy by employing data augmentation techniques during preprocessing, using the pre-trained "google/vit-base-patch16-224" model as a base. The ViT model was fine-tuned and evaluated on a Kaggle dataset of 8,000 MRI images categorized into four stages: ND, VMD, MD, and MoD. Methodologically, the study’s validation was confined to a simple train/validation/test split of this single Kaggle dataset. This approach lacks the rigor of more robust validation schemes like k-fold cross-validation and, crucially, does not include any external validation on a clinical dataset such as ADNI or OASIS. The approach achieved strong performance metrics on the test set: 98.19% accuracy, 96.34% sensitivity, 98.80% specificity, and a 96.37% F1-score. However, the high performance should be interpreted with caution, as it was achieved on a balanced, pre-processed dataset and has not been validated on independent, more heterogeneous clinical data, leaving the model’s real-world generalizability unconfirmed.

**Fig. 20:**
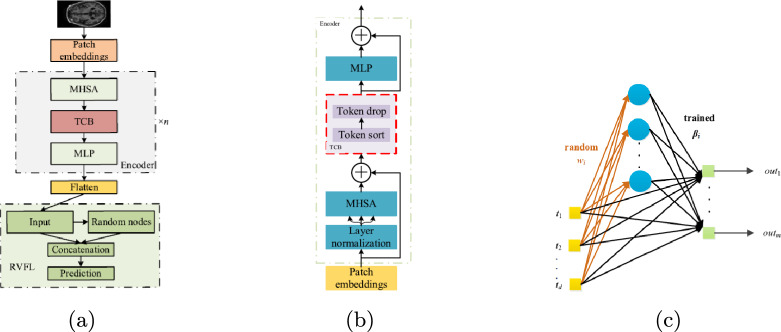
The Figures adapted from [202]: Key innovations of the RanCom-ViT model for efficient AD classification.** a** The overall framework, which modifies a standard ViT for greater efficiency and generalization. The two primary modifications are** b** the TCB, which increases inference speed by removing less important tokens, and** c** the RVFL-inspired head, which is designed to mitigate overfitting and improve robustness

Recently, Lu et al.  [202] developed RanCom-ViT, an efficient ViT-based method for AD classification using 2D MRI slices. The architecture (Fig. 20a) leveraged a pre-trained ViT backbone and introduced two key modifications for enhanced efficiency and generalization. Firstly, Token Compression Blocks (TCBs) were inserted after MHSA layers (Fig. 20b) to dynamically prune less informative patch tokens, thereby reducing computational load. Secondly, the standard ViT classification head was replaced with a Random Vector Functional-Link (RVFL)-inspired structure (Fig. 20c) to mitigate overfitting and improve generalization. Employing transfer learning, RanCom-ViT was fine-tuned on the OASIS-1 dataset (86,437 MRI slices, four dementia stages). The evaluation relied on a single 80/20 train/test split of the OASIS-1 dataset. While the dataset’s large size adds a degree of robustness, this validation strategy does not include k-fold cross-validation or, more critically, external validation on a different clinical cohort. The model achieved 99.54% accuracy, with ablation studies confirming the criticality of pre-training and the TCB’s role in significantly increasing inference speed. The exceptionally high accuracy, coupled with the improved efficiency, marks this as a promising architecture; however, its performance has only been demonstrated on a single data source. The absence of external validation means that the model’s ability to generalize to images from different scanners or patient populations remains an important, unverified question.

*Second: the studies that employ PET imaging for the diagnosis of AD:* In 2023, Shin et al. [162] proposed the application of a ViT for classifying AD stages using 18F-Florbetaben PET images. This study directly compared the performance of a pre-trained ViT against the established VGG19 CNN architecture. Preprocessing involved standard PET analysis steps, followed by the conversion of 3D volumes into 2D axial slices. Data augmentation through image rotation was also explored. Methodologically, the study’s evaluation was constrained by its reliance on a small, single-center hospital cohort and the use of a simple hold-out validation split, which may limit the broader generalizability of the findings. The models were evaluated for both binary (normal vs. abnormal) and ternary (HC vs. MCI vs. AD) classification tasks. ViT demonstrated slightly better performance than VGG19 in the binary task (80.0% vs. 73.3% accuracy). However, it underperformed in the more challenging ternary classification (56.7% vs. 66.7%), indicating that for more complex, multi-stage diagnostic tasks, the ViT architecture did not offer a clear advantage over established CNNs in this context. A key finding was that data augmentation negatively impacted test performance despite improving validation metrics, suggesting either overfitting or that the augmentation strategy was not well-suited for the PET data, a critical consideration for studies working with limited datasets.

*Third: the studies that employ fMRI imaging for the diagnosis of AD:* In 2022, Zhang et al. [203] introduced the Diffusion Kernel Attention Network (DKAN), a Transformer-based model adapted for brain disorder classification using rs-fMRI data. Addressing the limitations of standard Transformers–namely large parameter requirements for small sample sizes and the inability to model non-linear interactions–the model replaced the standard dot-product attention with a kernel attention mechanism. This innovation reduced parameters and was designed to capture non-linear functional connections. Furthermore, a diffusion process was integrated over the kernel attention to incorporate wider interactions among indirectly connected brain regions. While the architectural innovations are notable, the model’s evaluation was conducted on a relatively small cohort from the ADNI dataset using a simple 80/20 train/test split. For the task of classifying MCI from HC, the model achieved an accuracy of 80.0%. This performance, while promising, is based on a limited validation protocol without k-fold cross-validation or testing on an external cohort, making it difficult to assess the true generalizability of the proposed kernel and diffusion mechanisms in more diverse clinical scenarios.

In another study, Sarraf et al. [204] suggested OViTAD, an optimized ViT model for AD classification using rs-fMRI and sMRI. The model leveraged transformer-based self-attention mechanisms to efficiently model long-range dependencies, enabling precise classification of different AD stages. The architecture was trained and evaluated on the ADNI dataset for a three-class task (HC, MCI, and AD). Methodologically, the study’s validation relied on averaging results over three random 80/10/10 train-validation-test splits. While repeating the splits adds a measure of stability, this approach does not constitute a rigorous external validation on an independent cohort, and it is unclear if the extensive preprocessing pipeline was applied before or after data partitioning, which could introduce a risk of data leakage. The model achieved a high F1-score of 97.0% on rs-fMRI and 99.55% on sMRI, reportedly outperforming existing methods while utilizing 30% fewer parameters than a standard ViT. These high performance metrics underscore the potential of the optimized architecture, but their reliability is contingent on the methodological rigor of the data splitting and preprocessing steps. Without confirmation of a strict separation between training and testing data throughout the entire pipeline, the results should be considered with caution.

To jointly capture temporal dynamics and spatial inter-regional dependencies within rs-fMRI for AD diagnosis, He et al. [205] proposed a Spatiotemporal Graph Transformer Network (STGTN). The model addressed limitations of existing methods by jointly capturing both temporal dynamics within regional time series and spatial dependencies between brain regions. It used temporal transformer encoders for each brain region’s time series, followed by a spatial transformer encoder that employed a graph messaging mechanism incorporating functional connectivity (FC) as edge features. To combat the issue of limited rs-fMRI samples, an adversarial training strategy was implemented to augment the training data. The methodology was evaluated using a 5-fold cross-validation approach on cohorts drawn from the ADNI dataset. While this represents a robust internal validation, the study did not test the model on any external, independent datasets to assess its generalizability. For both AD vs. NC and eMCI vs. lMCI classification, the STGTN with adversarial training achieved high accuracies of 92.58% and 85.27%, respectively, significantly outperforming several baseline and state-of-the-art models. These strong results highlight the power of the spatiotemporal transformer architecture, but its performance outside of the ADNI cohort remains unverified, a critical next step for confirming its broader clinical utility.

**Fig. 21:**
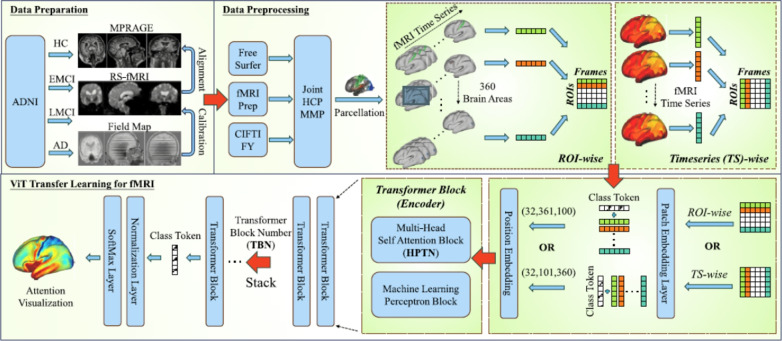
The operational pipeline of the VTFF model. The key stages are: (1) flattening of preprocessed 4D fMRI data into a 2D sequence; (2) tokenization via patch, positional, and class token embeddings; (3) processing through a multi-layer ViT encoder to capture spatio-temporal dependencies; and (4) classification based on the final output of the class token. Figure adapted from [206]

Recently, Wang  [206] introduced the ViT Transfer Learning for fMRI (VTFF) framework, adapting the ViT architecture for multi-class classification of cognitive decline stages (HC, EMCI, LMCI, AD) using rs-fMRI BOLD signals. The core innovation was the direct application of ViT’s attention mechanisms to spatio-temporal fMRI data, bypassing explicit functional connectivity matrix construction. The methodology (Fig. 21) began with standardized preprocessing of rs-fMRI data and parcellation into brain regions. A critical step was flattening the 4D fMRI data into a 2D sequence, with the study identifying a "Time-Series Wise" (TS-wise) strategy as superior. The VTFF model then followed a standard ViT encoder architecture to process the flattened sequence and perform classification. The model’s evaluation was based on a single 80/20 train/test split of a large cohort of 1,798 participants from the ADNI-2 dataset. While the cohort size is substantial, this validation approach does not include k-fold cross-validation or, more importantly, testing on an external, non-ADNI cohort. VTFF achieved 84.2% accuracy in the four-class task. Layer-wise attention visualization highlighted associations between cognitive impairment and specific cortical areas, aligning with connectomics research but via a more direct learning approach. This result demonstrates the promise of applying transformers directly to fMRI time-series; however, the lack of external validation means the model’s generalizability to data from different clinical sites or scanner protocols remains unconfirmed.

#### Single-modality MCI-to-AD conversion prediction

This subsection focuses on the application of ViT models, utilizing single-modality inputs such as sMRI or PET, for the specific task of predicting the conversion from MCI to AD. The reviewed studies explored the utility of these neuroimaging modalities in identifying early prognostic indicators within a ViT framework (see Table 4). In 2023, Hoang et al. [207] proposed the ViT model for predicting the progression from MCI to AD using mid-sagittal sMRI. The model leveraged ViT for feature extraction, capitalizing on its ability to capture global spatial dependencies across image patches. The model was trained and evaluated on the ADNI dataset, consisting of 598 MCI subjects. The study’s validation was based on a simple 90/10 train/test split of this single, modest-sized cohort. This methodology lacks the rigor of k-fold cross-validation and does not include external validation, which is a significant limitation when assessing the robustness of the findings. The model achieved 83.27% accuracy, with a specificity of 85.07% and a sensitivity of 81.48%. While these results are presented as state-of-the-art for this specific task, they should be interpreted with caution given the small cohort size and the absence of more rigorous validation, which leaves the model’s generalizability to other clinical datasets unconfirmed.

Khatri and Kwon [166] developed an explainable ViT framework incorporating self-supervised learning (SSL) to predict the progression of MCI to AD using 18F-FDG PET neuroimaging. The model leveraged the DINO SSL technique for pre-training a ViT backbone, enabling the extraction of meaningful features from unlabeled PET scans and reducing dependence on large annotated datasets. An Extreme Learning Machine (ELM) subsequently classified these extracted features to distinguish between stable MCI (sMCI) and progressive MCI (pMCI). The framework’s performance was assessed using a 5-fold cross-validation strategy on the ADNI dataset. While this represents a robust internal validation protocol, the study did not include an evaluation on an external, non-ADNI cohort to test for broader generalizability. The model achieved high classification performance with 92.31% accuracy, 90.21% specificity, and 95.50% sensitivity for pMCI vs. sMCI prediction. These results strongly suggest the efficacy of combining SSL with ViTs for this challenging prognostic task, although the absence of external validation means the model’s performance on data from different clinical sites or PET scanners remains unconfirmed.

#### Single-modality AD classification and MCI conversion prediction

Subsection 7.1.3 addresses the research literature that employs ViT-based models with single-modality sMRI data to tackle both AD classification and the prediction of MCI-to-AD conversion within a unified framework. This dual-objective approach was analyzed for its comprehensive diagnostic and prognostic capabilities using a singular data source.

**Fig. 22:**
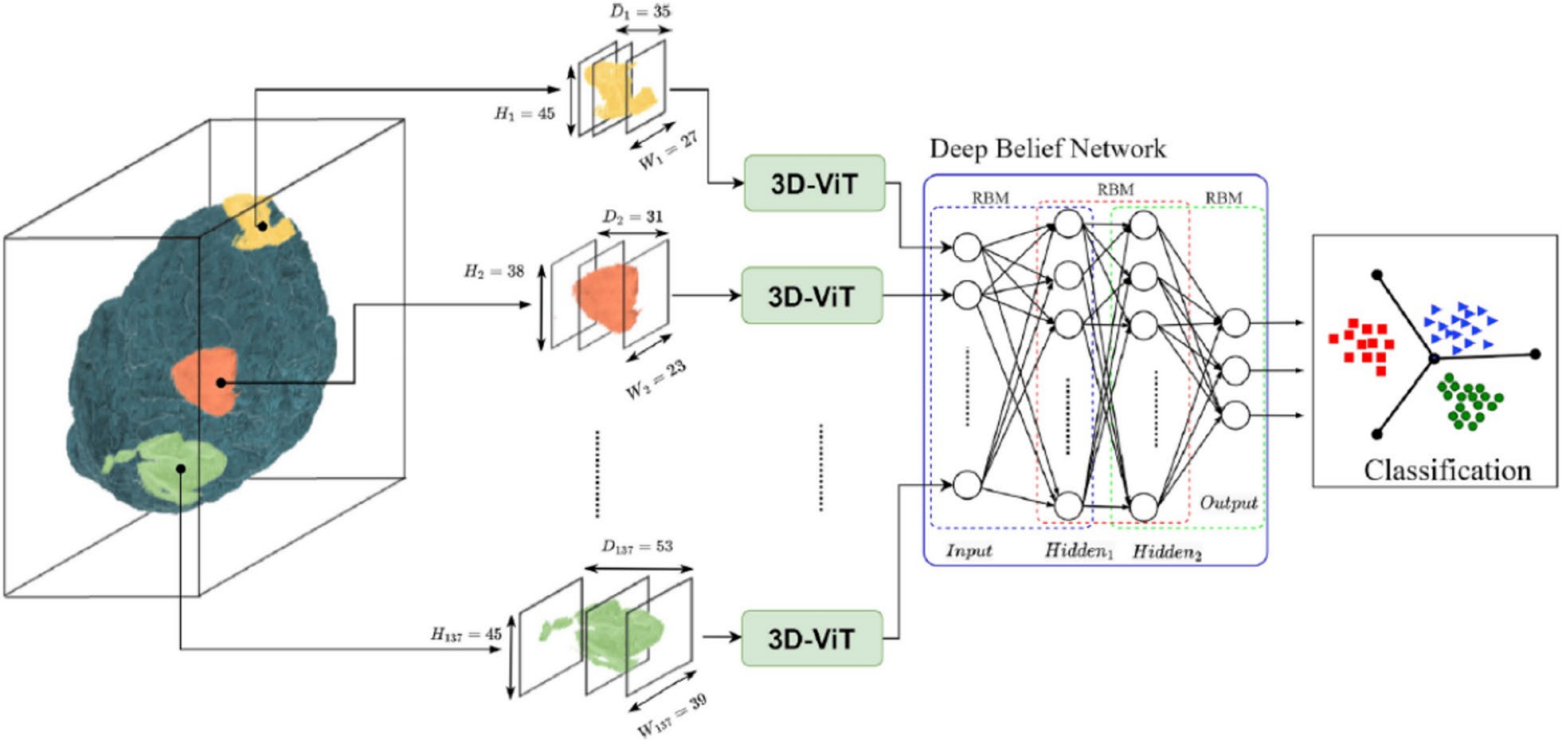
The operational pipeline of the Saoud and AlMarzouqi method. The key stages are: (1) Segmentation of a 3D sMRI scan into 138 anatomical ROIs; (2) Independent feature extraction from each ROI using a dedicated 3D-ViT; and (3) Integration of all ROI-level features by a DBN for final ensemble classification. Figure adapted from [163]

In 2024, Saoud and AlMarzouqi [163] proposed an explainable DL framework, ROI-3DViT-DBN, for early AD diagnosis and MCI conversion prediction (pMCI vs. sMCI) using baseline sMRI data from ADNI. Their approach (Fig. 22) involved segmenting preprocessed brain volumes into 138 anatomical ROIs. Each ROI was then independently processed by a dedicated 3D-ViT to extract region-specific features. To integrate these localized insights, the outputs from all 138 ROI-specific 3D-ViTs were fed into a Deep Belief Network (DBN), acting as an ensemble meta-learner. This ROI-centric design inherently supported explainability by allowing assessment of individual brain regions’ diagnostic contributions. Methodologically, the evaluation was performed on an 80/20 train/validation split of the ADNI dataset, with all reported results reflecting performance on the 20% validation set. The study did not employ a separate, untouched test set or an external cohort for final validation, which is a critical limitation for assessing the model’s true generalization performance. The ROI-3DViT-DBN system reportedly outperformed five comparison models, achieving high accuracy across various binary tasks (e.g., 90% for AD vs. CN; 94% for pMCI vs. sMCI). While the framework’s explainable design is a significant strength, the reported accuracies should be considered preliminary until they are verified on a true, held-out test set and validated on external, multi-center data.

### Applications of ViT with multimodality

Subsection 7.2 shifts the focus to investigations employing ViT architectures with multimodal data inputs, aiming to enhance diagnostic and prognostic accuracy by integrating information from diverse sources. The following subsections detail studies focused on AD classification (Subsect. 7.2.1) and both AD classification and MCI-to-AD conversion prediction (Subsect. 7.2.2), highlighting the specific data combinations and fusion strategies utilized within ViT frameworks.

#### Multimodal AD classification

This subsection details research that leverages multimodal data, such as combinations of sMRI and clinical or genetic information, in conjunction with ViT models for the classification of AD. The reviewed studies explored how the integration of these diverse data types within a ViT architecture contributed to improved diagnostic performance.

**Fig. 23:**
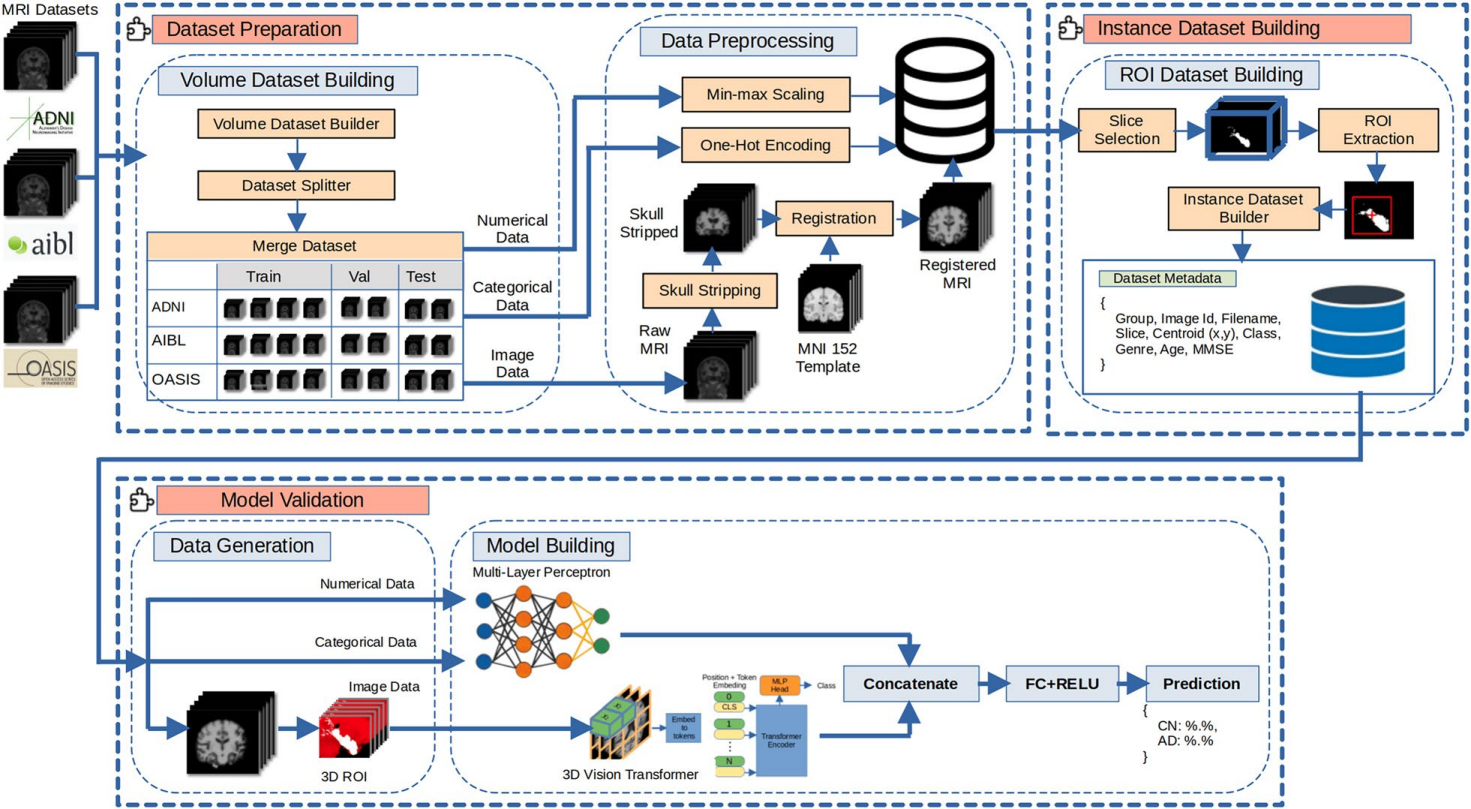
The dual-pathway architecture of the proposed MIMD-3DVT model. It concurrently processes 3D sMRI data with a ViT and tabular clinical data with an MLP, fusing the outputs for a unified multimodal AD diagnosis. Figure adapted from [191]

In 2024, Castro-Silva et al. [191] introduced the Multiple Inputs and Mixed Data 3D ViT (MIMD-3DVT) for AD classification, designed to integrate 3D sMRI data with non-imaging clinical information. MIMD-3DVT employed a dual-pathway architecture (Fig. 23) where clinical data were processed by an MLP, while 3D sMRI ROIs were processed by an adapted Video ViT (ViViT). The framework utilized an Intermediate (Single-Level) fusion approach (Fig. 14(b)), concatenating latent representations from both pathways before final classification. Methodologically, the study’s strength lies in its use of a combined multicenter dataset (ADNI, AIBL, OASIS) and a robust 7-fold cross-validation protocol, which enhances the potential for generalizability compared to single-cohort studies. Evaluated on this merged dataset, MIMD-3DVT achieved 97.14% accuracy for NC vs. AD classification when using multiple ROIs with mixed data, reportedly outperforming several contemporary methods. However, it is important to note two key limitations: the validation, while multi-centric, was performed on a merged data pool rather than a true external hold-out test from an unseen dataset. Furthermore, the study focused solely on the binary AD vs. CN classification, omitting the more diagnostically challenging and clinically critical task of identifying or staging MCI. Individual ROI analysis, such as with the entorhinal cortex, yielded accuracies up to 98.4%, highlighting its efficacy in 3D spatial processing and multimodal fusion.

#### Multimodal AD classification and MCI conversion prediction

Subsection 7.2.2 reviews the use of ViT architectures with multimodal data, typically integrating rs-fMRI information with SNP data, to perform both AD classification and MCI-to-AD conversion prediction. It focuses on how these modalities were fused within the ViT framework.

In 2024, Zou et al. [109] introduced BIGFormer, a sophisticated Graph Transformer architecture for both AD classification and MCI progression prediction. The framework was specifically designed to model the complex, multi-level interactions between genetic risk factors (SNPs) and brain functional phenotypes (rs-fMRI). At its core, the methodology constructed a subject-specific interaction graph where nodes represented either brain regions or risk genes. For data integration, the framework employed a Hybrid (Graph-based) fusion approach, transforming heterogeneous data into a unified graph structure. Key architectural innovations included a Perception with Local Structure Awareness (PLSA) module to encode graph topology and a Global Reliance Inference Component (GRIC) to model higher-order dependencies. The model underwent a robust evaluation using 10-fold nested cross-validation on an ADNI cohort of 708 subjects. BIGFormer yielded advanced classification results, achieving accuracies of 91.87% for HC vs. AD and a notable 85.71% for sMCI vs. pMCI classification. While the architectural novelty and strong internal validation are significant strengths, the clinical translation of such a complex graph-based model presents considerable challenges. Its computational complexity and the difficulty in intuitively interpreting the learned high-order interactions between genetic and neuroimaging features remain barriers to adoption. Furthermore, despite the robust cross-validation, the model’s performance has not yet been tested on a fully independent, external cohort, which is a necessary step to confirm the generalizability of its findings beyond the ADNI dataset.

### Analysis of ViT applications

A comprehensive analysis of the 22 reviewed studies on standalone ViT applications in AD research, as outlined in Fig. 15 and detailed in Table 4, provides critical insights into the current state and capabilities of this foundational transformer paradigm. This synthesis reveals a landscape where the promise of pure ViTs is actively being explored. The discussion covers the general operational pipeline (Subsect. 7.3.1), trends in data utilization (Subsect. 7.3.2), the predominant diagnostic focus (Subsect. 7.3.3), observations on multimodal integration (Subsect. 7.3.4), and noted technological variations (Subsect. 7.3.5).

#### General operational pipeline in ViT applications

A systematic examination of the research employing ViTs for AD classification and MCI prediction reveals a discernible, albeit adaptable, operational pipeline. A significant majority of these studies concentrate on single-modality data, with investigations leveraging ViTs for multimodal data integration being comparatively infrequent. For single-modality applications, which predominantly utilize 3D neuroimaging data such as sMRI, the workflow typically commences with a rigorous preprocessing cascade. Standard steps often include spatial normalization, skull stripping, bias field correction, and registration to a common stereotactic space (exemplified in Fig. 5 for sMRI). The specific preprocessing protocols are inherently modality-dependent; for instance, fMRI data necessitates distinct processing pathways, including motion correction and temporal filtering (e.g., [206], Fig. 21), compared to sMRI.

Following preprocessing, the volumetric data is transformed into a format amenable to ViT processing. Common strategies involve partitioning the 3D volume into a sequence of 2D slices ([134], Fig. 18), segmenting it into 3D patches, or extracting specific ROIs for targeted analysis ([163], Fig. 22). These prepared inputs are then processed through the ViT architecture, characterized by its initial patch (or token) embedding stage and subsequent sequential transformer encoder blocks that leverage self-attention mechanisms to model global contextual relationships (as detailed in Subsect. 4.4). Notably, several investigations report architectural modifications to the standard ViT. These adaptations, which may include novel attention mechanisms, structural optimizations, or altered layer configurations, are typically aimed at enhancing computational efficiency or improving diagnostic accuracy (e.g., [202], Fig. 20). Furthermore, the feature representations derived from the ViT are not always directly fed to a terminal classifier; some methodologies incorporate intermediate layers or specialized modules to further refine these features before classification, often via an MLP or a similar structure ([197], Fig. 19). The utilization of pre-trained ViT backbones, fine-tuned on AD-specific datasets, is also a recurrent theme.

In contrast, the application of standalone ViTs to multimodal datasets is less prevalent in the reviewed literature. In the few studies that do employ ViTs with multimodal data, a common paradigm involves utilizing the ViT for deep feature extraction from neuroimaging modalities. Concurrently, non-imaging data (e.g., clinical scores, demographic information, genetic markers) undergoes separate preprocessing (e.g., normalization, encoding) and feature extraction, often through dedicated MLPs or simpler neural network structures. The resultant modality-specific feature sets are subsequently integrated using various fusion strategies. Based on our review, these fusion strategies were identified as:

- *Intermediate (Single-level) fusion:* This approach was used by Castro-Silva et al. [191], where latent representations from a 3D ViT (processing sMRI) and an MLP (processing clinical data) were concatenated before final classification (Fig. 23).
- *Hybrid (Graph-based) fusion:* A more complex strategy was employed by Zou et al. [109], who constructed a unified interaction graph from fMRI and SNP data, which was then processed by a Graph Transformer.

**Fig. 24:**
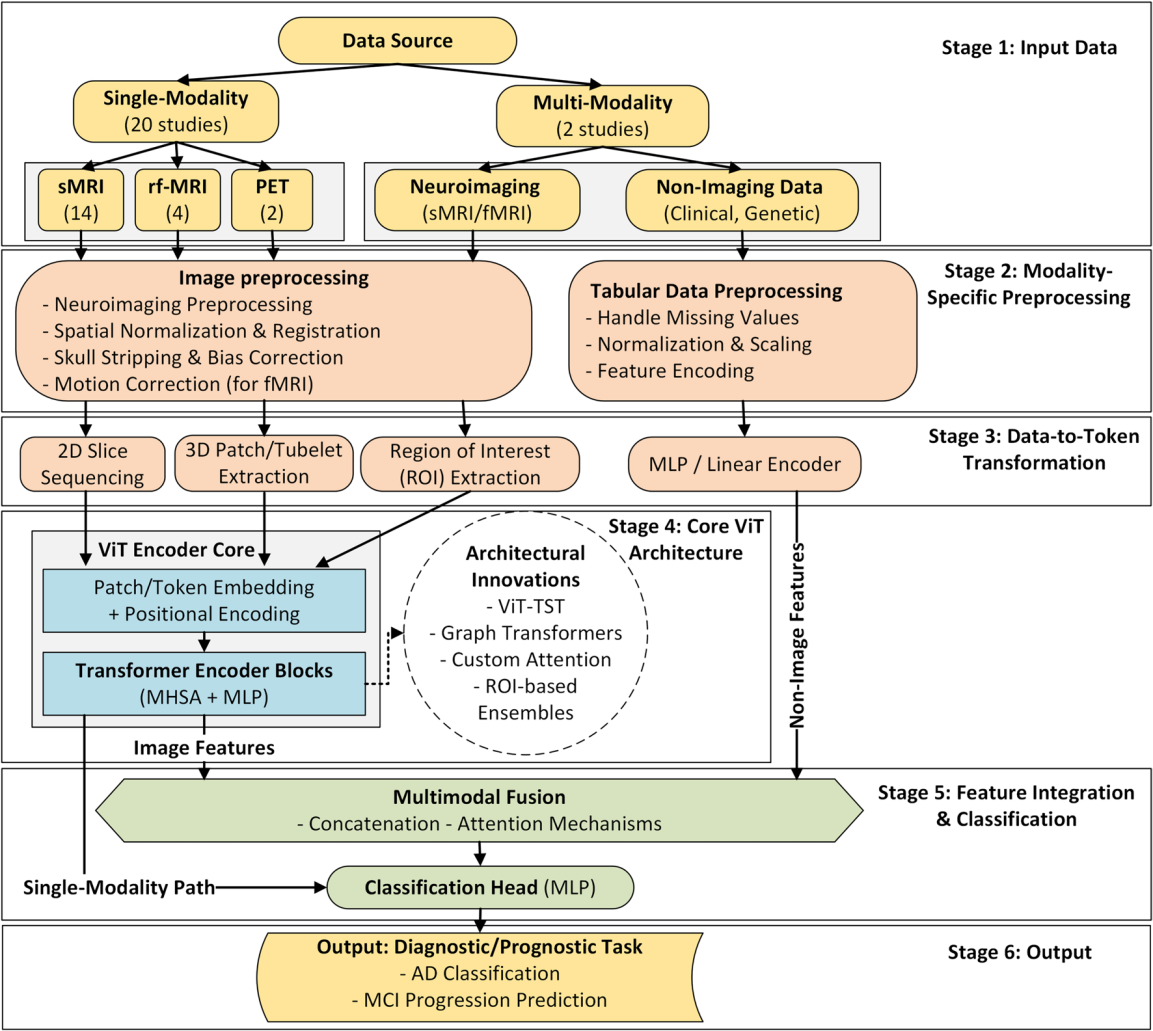
The general operational pipeline for standalone ViT applications in AD research, as synthesized from the reviewed literature

This general workflow—encompassing data acquisition, modality-specific preprocessing, ViT-based feature learning (often with bespoke modifications), and classification or fusion—forms the structural basis for the application of ViTs in AD research observed across the surveyed literature. A schematic of this generalized pipeline is illustrated in Fig. 24.

#### Observed trends in modality and dataset utilization

The analysis of the 22 reviewed ViT studies indicates an overwhelming reliance on sMRI as the primary input modality, constituting approximately 70% (14 out of 20) of single-modality applications (e.g., [134, 161]). In comparison, PET (e.g., [162, 166]) and rs-fMRI (e.g., [203, 204]) were explored in fewer single-modality studies, representing about 10% (2 out of 20) and 20% (4 out of 20) respectively. This distribution is also visualized in Fig. 17.

A concurrent strong dependence on the ADNI dataset was noted, featuring in roughly 73% (16 out of 22) of all examined ViT studies (e.g., [134, 191]). While other resources like OASIS [158, 161] and Kaggle datasets [196, 197] were also employed, the prevalence of ADNI for primary development potentially limits the generalizability of some findings.

#### Predominant diagnostic focus

The vast majority of the 22 ViT applications centered on AD classification tasks (e.g., [134, 162, 191]), encompassing binary classifications (AD vs. CN), multi-class staging (AD vs. MCI vs. CN), or finer categorizations such as the four stages (ND, VMD, MD, MoD) common in Kaggle dataset studies [196, 197]. High accuracies, often exceeding 95% or 99%, were reported in several instances [134, 195].

Significantly less research attention was directed towards predicting MCI-to-AD conversion. Only two studies focused solely on this using single-modality data (sMRI in [207]; PET in [166]), and one study by Saoud and AlMarzouqi [163] addressed both classification and MCI conversion with sMRI.

#### Observations on multimodal integration and generalization testing

Within the reviewed 22 standalone ViT studies, explicit multimodal data integration was infrequent, observed in only two studies (approximately 9%) [109, 191]. These typically combined sMRI or fMRI with clinical or genetic information. This low prevalence, compared to what is generally seen in CViT literature (Section 8), suggests that deep fusion in pure ViT frameworks is less common. Furthermore, rigorous evaluation of model generalizability via external validation was limited. While some studies used external datasets like OASIS [158, 161] or AIBL [191], most relied on internal cross-validation or hold-out sets from the primary dataset. Explicit reporting of robust external validation on truly independent cohorts was found in only a small fraction of the single-modality ViT studies.

#### Noted technological variations and architectural innovations

Beyond standard implementations, the reviewed studies demonstrated a notable trend of architectural innovation, adapting the foundational ViT principles to better address the specific challenges of neuroimaging and AD-related data. Consistent with the flexibility of the general operational pipeline, these variations underscore a dynamic area of research. For instance, to better capture the volumetric nature of 3D MRI, Alp et al. [134] developed the ViT-TST, which integrates a time-series transformer to model inter-slice dependencies. Targeting specific neuropathological regions, Saoud and AlMarzouqi [163] employed an ROI-based analysis with an ensemble of 3D-ViTs and a Deep Belief Network. In the domain of functional neuroimaging, adaptations were made to handle the unique structure of fMRI data, such as the use of diffusion kernel attention by Zhang et al. [203] to model non-linear brain region interactions. Graph-based approaches also emerged, with He et al. [205] proposing a spatiotemporal graph transformer (STGTN) for rs-fMRI, and Zou et al. [109] developing BIGFormer, a graph transformer for integrating rs-fMRI with multimodal genetic data. Furthermore, to address the challenge of limited labeled data, Khatri and Kwon [166] successfully applied self-supervised learning strategies to PET analysis. Finally, for handling mixed data types, Castro-Silva et al. [191] engineered a specialized 3D ViT (MIMD-3DVT) capable of processing both imaging and clinical information. Collectively, these technological variations highlight a significant research effort dedicated to tailoring ViT architectures for the nuanced demands of AD diagnostics.

In summary, the analysis of standalone ViT applications reveals several distinct patterns in the literature, including a primary focus on single-modality classification, a strong reliance on the ADNI dataset, and an initial exploration into multimodal data integration. The observed architectural characteristics of pure ViTs, particularly their emphasis on global self-attention for context modeling, provide an important framework for understanding the field’s progression. This natural architectural evolution towards hybrid models, which aim to synergize these global capabilities with the local feature extraction strengths of CNNs, is analyzed in the next section.

## Convolution vision transformers in Alzheimer’s disease domain

Following the exploration of standalone ViTs, this section focuses on hybrid architectures, specifically CViTs, and their application in AD research. The limitations of pure ViTs in capturing fine-grained local features, combined with their heavy data and computational requirements, created a clear need for architectures that could blend their global context capabilities with the proven efficiency and strong inductive biases of CNNs [136]. This necessity directly motivated the research community’s pivot towards hybrid CViT models. Building upon the principles outlined in Subsect. 4.4.2, CViTs integrate convolutional layers for local feature extraction with transformer blocks for global context modeling [208]. This section systematically analyzes 46 such studies, identified in Fig. 15 and detailed in Table 5, structuring the discussion by input data modality: single-modality applications (Subsect. 8.1) and multimodal frameworks (Subsect. 8.2). A comprehensive analysis and concluding remarks are presented in Subsect. 8.3.

### Applications of CViT with single-modality

**Fig. 25:**
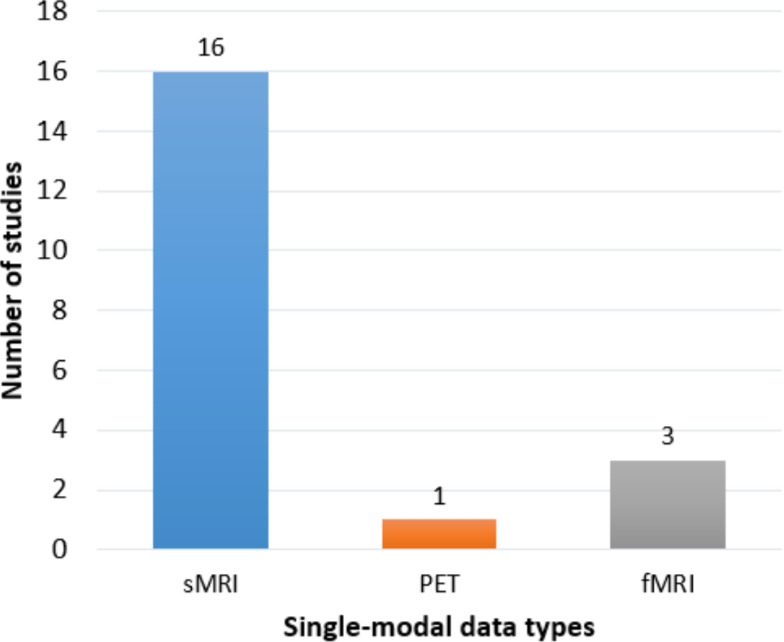
The bar chart displays an overview of medical data types used in single-modality studies using CViT

This subsection reviews 20 studies that employed CViT-based models with single-modality data for AD classification, prediction of MCI progression, or both. As categorized in Fig. 15, these investigations are grouped by their diagnostic aim: AD classification (Subsect. 8.1.1), MCI progression prediction (Subsect. 8.1.2), and studies addressing both objectives (Subsect. 8.1.3). Figure 25 illustrates the distribution of specific single-modality data types utilized in these CViT applications.

#### Single-modality AD classification

Subsection 8.1.1 specifically examines research that applied hybrid CViT architectures with single-modality neuroimaging data, predominantly sMRI, for AD classification. The reviewed studies investigated various CViT designs and their efficacy in distinguishing AD stages. Subsequent discussions also cover applications that used PET and fMRI.

*First: the studies that employed sMRI imaging for the diagnosis of AD:* In 2021, Kadri et al. [153] presented a hybrid DL architecture for AD classification using sMRI. The model’s design exemplified a Serial Integration strategy that also incorporated elements of Channel Attention, as detailed in Subsect. 4.4.2. In this sequential pipeline, conceptually illustrated in Fig. 13(b), a Wide Residual Squeeze-and-Excitation Network (WRSE-Net) first acted as a feature extraction front-end to process the input MRI and learn localized feature maps. The integrated Squeeze-and-Excitation modules performed channel attention, a mechanism shown in Fig. 13(g), to adaptively re-weight the most informative feature channels. These extracted features were subsequently fed into the CrossViT component, which leveraged its self-attention mechanism to model global context. A key component of their methodology was the use of a self-attention Progressive Generative Adversarial Network (ProGAN) for data augmentation, which addressed the common challenge of limited data availability. The model was evaluated using MRI data from the ADNI and OASIS databases for classifying CN, MCI, and AD stages. However, the study provides insufficient detail regarding its validation protocol; critical information such as data splitting ratios, the use of subject-level partitioning, and whether the cohorts were used for external validation is not specified. The proposed hybrid model achieved a notable accuracy of 99%. While this result appears impressive, the lack of methodological transparency makes it difficult to assess its validity and risk of data leakage or overfitting. Without a clear and reproducible validation strategy, the reported accuracy should be interpreted with significant caution.

Zhu et al. [160] introduced Brain Informer (BraInf), a DL architecture for AD classification using 3D sMRI. BraInf’s design followed a Hierarchical Integration pattern, as conceptually illustrated in Fig. 13(c) and detailed in Subsect. 4.4.2. It efficiently captured global contextual information by processing 3D sMRI patches through stacked layers featuring two key innovations: multi-head ProbSparse self-attention and a structural distilling operation. This interleaving of the convolution-based structural distilling operation with the attention mechanism within the model’s layers is characteristic of hierarchical designs. The model was validated using a 10-fold cross-validation scheme on the ADNI dataset. While this represents a robust internal validation, the study did not include an evaluation on any external, non-ADNI cohorts, which is a notable limitation for assessing broader generalizability. BraInf achieved high accuracies of 97.97% for AD vs. NC and 91.89% for MCI vs. NC classification, demonstrating its efficacy in learning discriminative global features from sMRI with computational efficiency. These strong results demonstrate the efficacy of its efficient architecture for learning discriminative features from sMRI; however, the lack of external validation means its performance on data from different clinical sites and scanner protocols remains to be confirmed.

In 2023, Huang et al. [209] introduced the Resizer Swin Transformer (RST), a DL model for AD classification using sMRI. The model’s architecture represented a Serial Integration approach, as depicted in Fig. 13(b) and explained in Subsect. 4.4.2. It first incorporated a CNN-based Resizer module for cross-channel learning and adaptive image scaling, which aimed to reduce the need for extensive preprocessing. The output was then fed into a Swin Transformer for multi-scale spatial feature extraction. The model was trained and evaluated on the ADNI and AIBL datasets. While the use of both cohorts allowed for a form of external validation, the evaluation was based on a single 70/10/20 train-validation-test split rather than a more robust k-fold cross-validation protocol, which may make the results sensitive to the specific data partition. The model achieved an exceptionally high 99.59% accuracy on ADNI and 94.01% on AIBL, demonstrating superior sensitivity (99.59%) and specificity (99.58%) compared to several existing methods. The near-perfect performance on the ADNI test set, in particular, should be interpreted with some caution, as such high metrics achieved without a cross-validation framework can sometimes be prone to optimistic bias.

**Fig. 26:**
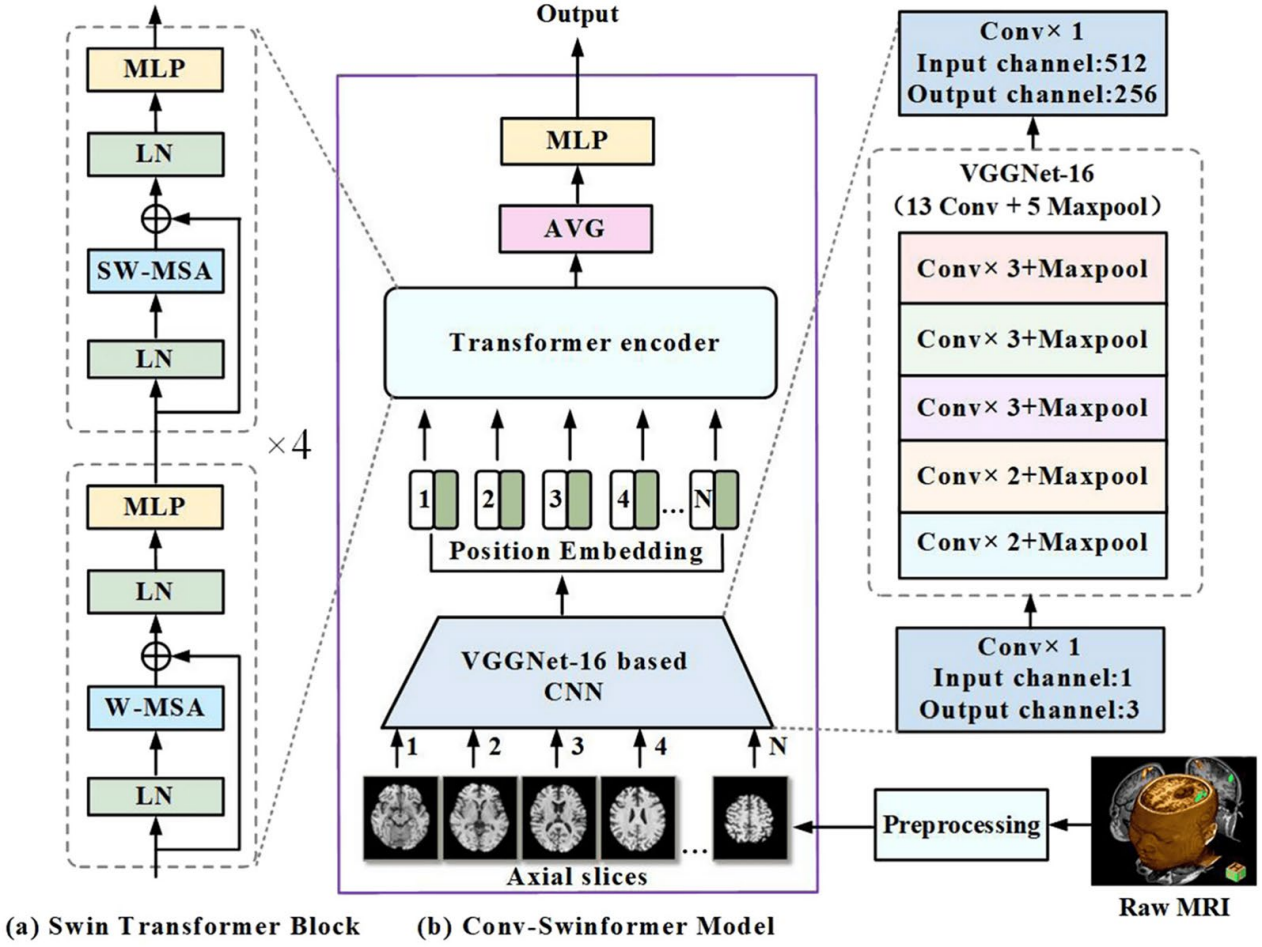
The Conv-Swinformer architectural pipeline.** a** The core Swin Transformer block, featuring shifted window self-attention.** b** The complete serial architecture, where a CNN acts as a feature extractor for 2D MRI slices, and a Swin Transformer processes the resulting feature sequence to model 3D spatial relationships for AD classification. Figure adapted from [81]

Hu et al.  [81] proposed Conv-Swinformer, a hybrid DL architecture that exemplifies a Serial Integration approach (Fig. 13(b)) for AD classification using sMRI data. In their two-stage pipeline (Fig. 26b), a VGGNet-16-based CNN module first acts as a feature extractor, processing preprocessed 3D sMRI volumes (e.g., via spatial normalization and skull stripping, as shown in Fig. 5) as sequences of 2D axial slices to generate feature tokens. Second, this sequence of tokens is fed into a Swin Transformer encoder (detailed in Fig. 26a) that models inter-slice dependencies using a Shifted-Window MHSA (SW-MHSA) mechanism. The model was primarily evaluated on the ADNI dataset, achieving 93.56% accuracy for AD vs. CN classification. Notably, the study also performed an external validation on the OASIS dataset, where it achieved 92.31% accuracy, demonstrating a degree of generalizability. However, the primary evaluation on the ADNI cohort was based on a single 70/15/15 train-validation-test split rather than a more robust k-fold cross-validation protocol, which may make the specific performance metrics sensitive to the particular data partition used.

To address computational demands while retaining spatial information, Xin et al. [31] proposed the Efficient Conv-Swin Net (ECSNet), a DL model for AD diagnosis using sMRI. The model utilized a Serial Integration architecture (Fig. 13b), combining a CNN for early-stage feature extraction with a Swin Transformer for hierarchical spatial feature learning. To balance accuracy and efficiency, a 2.5D-subject method was employed, encoding 3D structural information into 2D feature maps, and lightweight mechanisms were integrated to optimize performance. In a display of strong methodological rigor, the model was trained using 5-fold cross-validation on the ADNI dataset and then subjected to a true external validation on an entirely independent test set from the AIBL cohort. This robust evaluation protocol strengthens the credibility of their findings, achieving 92.8% balanced accuracy and 91.1% sensitivity on the external AIBL test set. The successful generalization from ADNI to AIBL is a significant result, demonstrating that the efficient architecture is not merely overfitted to a single dataset but has learned robust, transferable biomarkers of the disease.

**Fig. 27:**
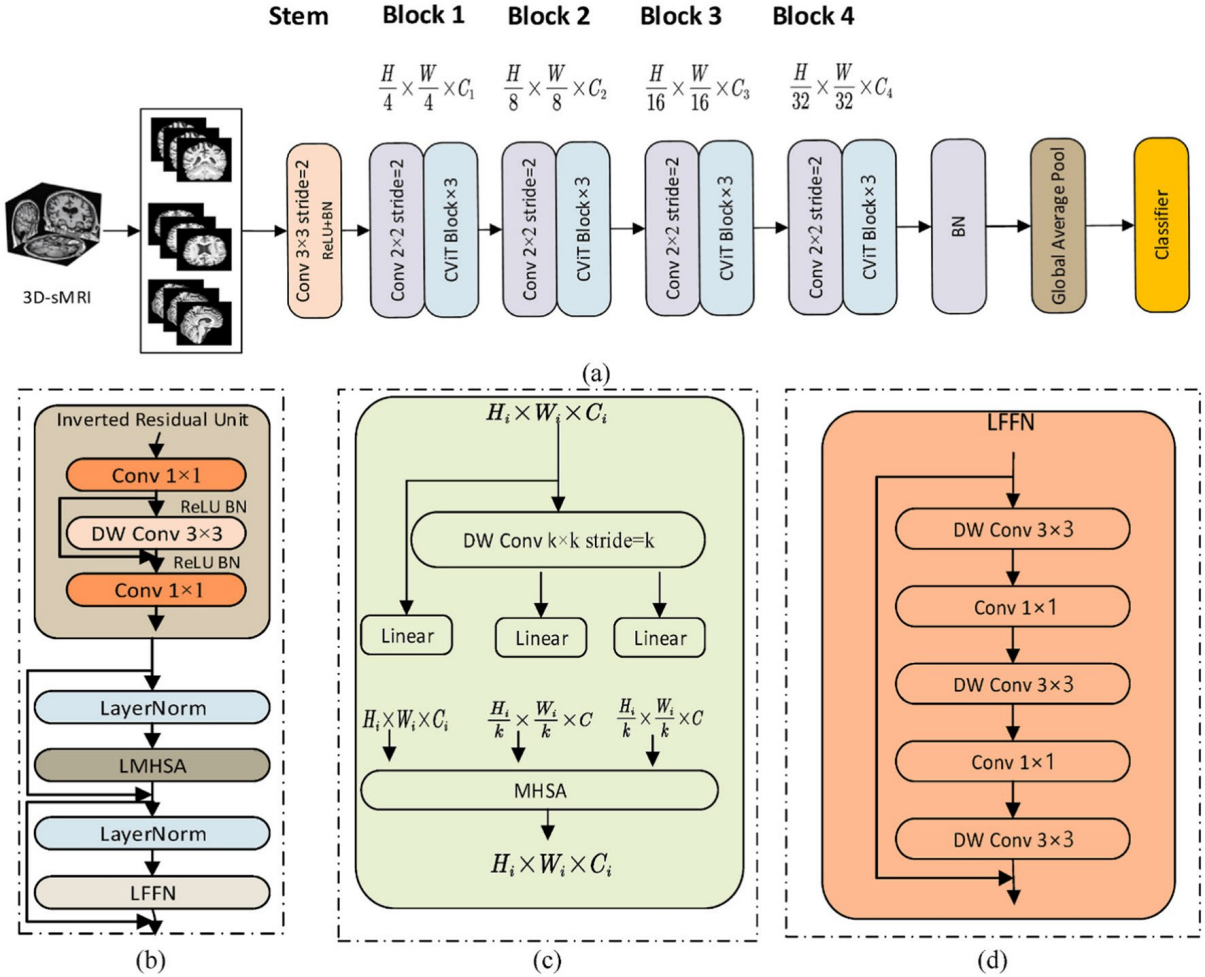
The framework of optimized CViT for AD,** a** Overall architecture** b** convolution transformer encoders with IRU, LMHSA and LFFN block,** c** LMHSA block with additional depth-wise convolution and** d** sandglass LFFN block with skip connection. Figure adapted from [30]

Khatri et al. [145] proposed a novel hybrid DL network for detecting AD and MCI using sMRI data. The architecture exemplified a Hierarchical Integration strategy (Fig. 13c), as it featured an adaptable, efficient receptive field achieved by alternating Mobile Convolution (MBConv) with block attention and Partial Convolution (PConv) with grid attention modules. This deep interleaving of specialized convolutional and attention blocks, similar to advanced models shown in Fig. 27, captured both local and global features effectively while reducing computational cost. An enhanced hierarchical inverted residual feed-forward network (IRFFN) further integrated features across layers. The model was trained and evaluated on the ADNI dataset using a 10-fold cross-validation protocol. While this method ensures robust internal validation, the study’s evaluation was limited to this single cohort, without testing on an independent external dataset. The model demonstrated high performance, achieving an average accuracy of 97.29% (AD vs. HC) and 94.79% (MCI vs. HC). These strong results highlight the potential of the novel hybrid attention-fusion architecture, but its generalizability to data from different clinical centers or scanners remains an important and unverified question.

Khatri and Kwon  [30] introduced an optimized lightweight hybrid convolution-attention model for AD and MCI detection using sMRI data from the ADNI dataset. Its design was a prime example of a Hierarchical Integration architecture (Fig. 13c), integrating efficient convolutional building blocks with enhanced multi-axis attention within a multi-stage, ViT-inspired framework (Fig. 27a). An initial convolutional *Stem* performed preliminary feature extraction, followed by four main *Blocks* (Fig. 27c) that alternated specialized convolution-attention modules with multi-axis attention mechanisms. These modules included MBConv (a MobileNetV2-based inverted residual block with Squeeze-and-Excitation modules) and PConv (Partial Convolution, Fig. 27b) for efficient receptive field processing. These were coupled with multi-axis attention mechanisms: Block Attention, which applies relative self-attention (RelAttn) to non-overlapping blocks after MBConv, and Grid Attention, which applies RelAttn to grid cells after PConv; the RelAttn incorporated a learnable relative position bias. Instead of a conventional MLP, an Inverted Residual Feed-Forward Network (IRFFN, Fig. 27d) was used post-attention. For training, the AdamW optimizer was augmented with Gradient Centralization (GC) for stabilized training. Evaluated on ADNI, the model achieved 95.37% accuracy for AD vs. HC, 92.15% for MCI vs. HC, and 94.31% for multi-class (AD/MCI/HC) classification, reportedly outperforming baseline CNNs and other ViT/CMT variants with notable sensitivity (97.14%) and specificity (94.11%). However, as the authors themselves note, a key limitation of the study is that its evaluation was predicated exclusively on the ADNI cohort. The absence of external validation on a diverse, multi-center dataset means that the model’s ability to generalize to different imaging protocols and patient populations remains unverified, a critical next step for validating such a novel architecture. *This pair of studies from Khatri et al. collectively demonstrated a focus on developing computationally efficient yet powerful Hierarchical Integration models tailored for sMRI analysis.*

Li et al. [144] developed the Landmark-based Multi-Instance Conv-Transformer (LD-MILCT) framework for brain disease classification, including AD, using T1-weighted sMRI. This model used a Serial Integration approach (Fig. 13b), where it first identified statistically significant brain landmarks and then used CNNs as a front-end to process local patches extracted around them. A ViT subsequently analyzed the relationships between these distributed patches to capture broader spatial correlations. The framework also employed a multi-instance learning head to ensure local patch information contributed effectively. Evaluated on the ADNI dataset for AD classification, the study employed a 10-fold cross-validation scheme on a moderately sized cohort. This approach, while providing a robust measure of internal validity, does not include an external validation on an independent dataset. The proposed LD-MILCT achieved 91.0% accuracy, demonstrating strong sensitivity (90.3%) and specificity (95.7%). While the novel landmark-based patch extraction method is a key contribution, the model’s performance should be interpreted with the understanding that its generalizability to data from different clinical sites or scanners has not yet been demonstrated.

**Fig. 28:**
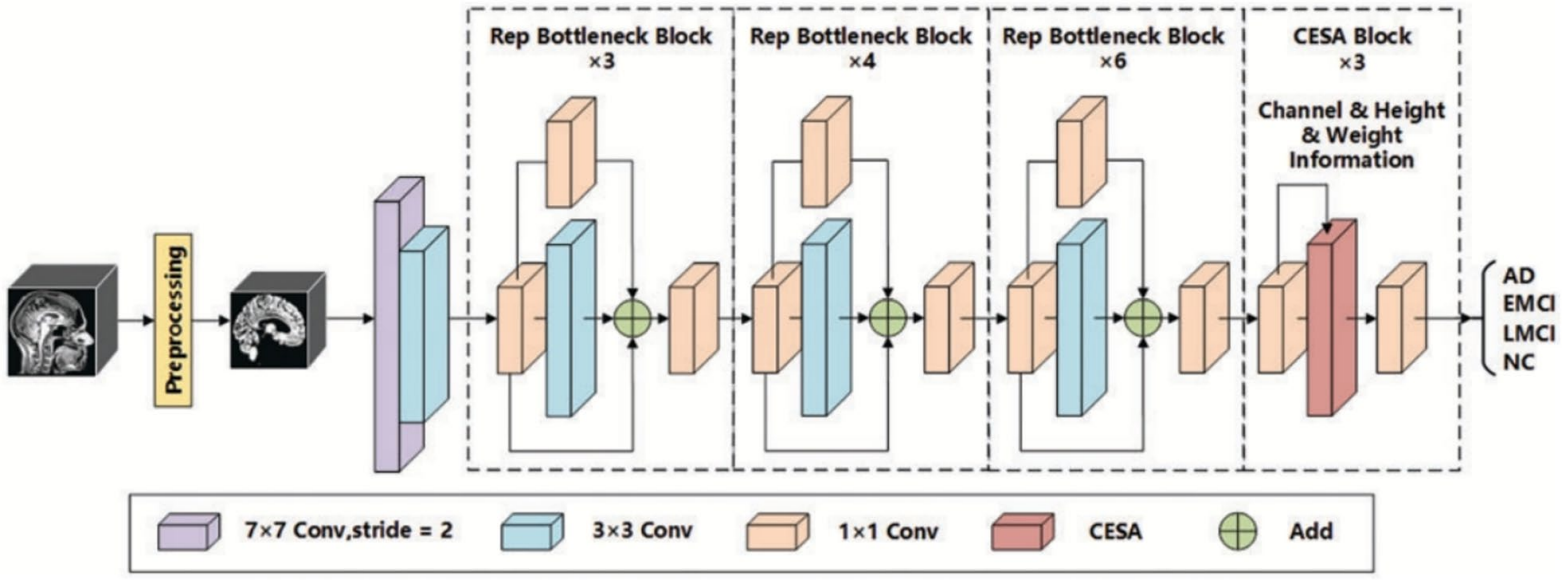
The AD-aided diagnostic pipeline of the RepBoTNet-CESA model. This framework modifies a Bottleneck Transformer to improve both computational efficiency during inference and the capacity to learn discriminative 3D lesion features from sMRI by introducing a specialized Cubic Embedding Self-Attention mechanism. Figure adapted from [157]

Zhang et al.  [157] proposed RepBoTNet-CESA, a DL network for the computer-aided diagnosis of AD using sMRI (Fig. 28). The architecture is a prime example of an MHSA Integration strategy (Fig. 13i), which itself is a specific implementation of the broader Serial Integration architectural pattern (Fig. 13b), where features from initial convolutional stages are passed to a final Transformer-based block. The model integrates two key innovations. First, it modifies a BoTNet-S1 baseline into a RepBoTNet by leveraging structural reparameterization. During training, standard 3x3 convolutions are replaced with multi-branch blocks to enhance feature learning. For efficient inference, these are fused into a single 3x3 convolutional layer by merging the batch normalization parameters ($\mu , \sigma , \gamma , \beta$) into new kernel weights ($W'$) and biases ($b'$):

39 $$\begin{aligned} W'&= W \frac{\gamma }{\sigma } \end{aligned}$$

40 $$\begin{aligned} b'&= \beta - \frac{\mu \gamma }{\sigma } \end{aligned}$$

Second, the model replaces the standard MHSA in deeper layers with a novel Cubic Embedding Self-Attention (CESA) mechanism. CESA was specifically designed to incorporate the often-disregarded channel dimension information present in sMRI, allowing the model to learn lesions from a three-dimensional, stereoscopic perspective. Evaluated on the ADNI dataset, the study’s validation was performed using a 5-fold cross-validation on a relatively small cohort of 105 subjects. While the architectural innovations are significant, the modest cohort size raises concerns about the potential for overfitting and limits the statistical power of the findings. RepBoTNet-CESA achieved high accuracy across various classification tasks, notably 96.58% for AD vs. NC, 92.75% for EMCI vs. NC, and 80.97% for the four-class AD vs. EMCI vs. LMCI vs. NC task. Given the small sample size and the absence of external validation, these high-performance metrics should be considered preliminary; further testing on larger, multi-center datasets is essential to confirm the robustness and generalizability of the RepBoTNet-CESA architecture.

Huang and Qiu  [210] introduced the Monte Carlo Ensemble ViT (MC-ViT), a novel DL framework for AD diagnosis using sMRI. This framework follows a Serial Integration pattern (Fig. 13b) and uniquely generates an ensemble prediction from a single ViT learner by leveraging Monte Carlo (MC) sampling. The model first utilizes a 3D patch CNN as a front-end to extract local spatial features and assess patch-level diagnostic relevance. Subsequently, MC sampling creates diverse feature subsets by first selecting patches based on this relevance (patch sampling) and then uniformly sampling features within those chosen patches (feature sampling). A modified ViT processes these varied, sampled feature sets to model global structural relationships, and the final diagnosis is determined by aggregating predictions across multiple MC samples (e.g., M=50 repetitions). The model was evaluated on large ADNI (7199 scans) and OASIS-3 (1992 scans) datasets with minimal preprocessing. The use of OASIS-3 as an external validation cohort is a significant methodological strength of this study, providing a crucial test of the model’s real-world generalizability. MC-ViT achieved a high Balanced Accuracy of 90.01% for NC vs. AD classification on ADNI. However, a notable performance drop was observed on the external OASIS-3 dataset, where the Balanced Accuracy decreased to approximately 80%. This discrepancy underscores the persistent challenge of domain shift between different clinical cohorts and highlights that even sophisticated ensemble techniques are not immune to the difficulties of generalization across varied patient demographics and imaging protocols.

*The following group of studies employed a different sMRI classification strategy, categorizing subjects into four distinct stages (ND, VMD, MD, and MoD) or (Early, Mild, High, and Normal).*

Poonia and Al-Alshaikh  [152] introduced an ensemble DL framework for AD diagnosis utilizing brain MRI data. The architecture’s integration strategy was twofold. It primarily represents a form of Late Fusion, as outlined in Subsect. 4.4.2 and conceptually shown in Fig. 13e, by integrating the final predictions from several prominent transfer learning (TL) models (InceptionV3, VGG19, ResNet50, DenseNet121) and a ViT. Each of these individual models, in turn, operated based on a Serial Integration design (Fig. 13b) to capitalize on their combined strengths. Emphasizing the need for transparency in smart healthcare, the study incorporated XAI using GradCAM for visualizing model attention. The model was evaluated on the ADNI dataset for classifying different AD stages (early, mild, high, and normal). However, the study’s validation methodology lacks transparency; critical details regarding the data splitting protocol (e.g., train/test ratios, subject-level partitioning) are not explicitly provided, which introduces a risk of methodological ambiguity and hinders reproducibility. Furthermore, the evaluation is confined to the ADNI cohort without any external validation. The ensemble TL-ViT architecture demonstrated substantial gains, achieving 96% accuracy, 94% precision, 90% recall, and a 92% F1-score. This performance significantly surpassed that of the individual TL models tested alone, which showed limited discrimination (approximately 58% accuracy and a 44% F1-score). While the performance improvement from ensembling is notable, the high accuracy should be interpreted with caution given the unspecified validation strategy and the absence of testing on an independent dataset to confirm generalizability.

In April 2024, Sait [141] introduced an integrated DL framework for AD diagnosis that followed a Parallel Integration architecture (Fig. 13a). The approach combined an enhanced LeViT (aViT) and an EfficientNet B7 model in concurrent streams to extract high-level features sensitive to AD-related patterns. The extracted information was then fused, and a Dartbooster XGBoost (DXB) classifier, optimized using Bayesian Optimization Hyperband (BOHB), was used for the final prediction. The system was trained on the OASIS dataset and tested for generalization on a separate Alzheimer’s dataset. The use of a distinct dataset for external validation is a significant methodological strength, directly addressing a common limitation in the field. The proposed method achieved a notably high generalization accuracy of 99.8% in classifying different AD stages (ND, VMD, MD, and MoD) with comparatively low computational demand. However, this near-perfect accuracy on an external dataset is an exceptional claim that warrants cautious interpretation. The validation was performed on a pre-processed Kaggle dataset, which may not fully represent the domain shift and variability (e.g., scanner noise, artifacts, different patient demographics) encountered in raw clinical data from a different institution. While the approach is promising, independent verification of these results on other, more challenging multi-center clinical cohorts is necessary to substantiate such high levels of generalizability.

In 2024, Sait and Nagaraj [187] introduced a feature-fusion technique for multi-stage AD classification that utilized a Parallel Integration design (Fig. 13a). Their approach combined a modified Compact Convolutional Transformer (CCT) with Linformer and a Twins Transformer (TT) with Performer in parallel branches to efficiently extract both local and global features while reducing the computational demands of standard transformers. An attention-based mechanism fused features from these streams, followed by Principal Component Analysis (PCA) for dimensionality reduction. An enhanced CatBoost model, optimized using Bayesian Optimization with Hyperband (BOHB) and quantization techniques for efficiency in resource-limited settings, performed the final classification, with SHAP values incorporated for model interpretability. The model was trained on the Kaggle Alzheimer’s dataset and demonstrated strong generalization on the OASIS dataset. The use of OASIS as a separate, external validation cohort is a key methodological strength, providing a direct test of the model’s ability to generalize. The study reported achieving 99.2% accuracy on the Kaggle test set and an exceptionally high 98.8% accuracy on the external OASIS cohort across four AD stages (ND, VMD, MD, and MoD). While the cross-dataset validation is commendable, the near-perfect generalization accuracy is an extraordinary result that warrants cautious interpretation. Such high performance is rarely seen when transferring models between datasets with different population demographics and acquisition protocols. Independent replication of these findings on other diverse, multi-center clinical datasets is essential to substantiate the robustness and real-world applicability of this complex feature-fusion framework. *These two studies from Sait et al. consistently applied Parallel Integration architectures to fuse features from different transformer backbones, showcasing strong performance on the Kaggle and OASIS datasets.*

Menon and Gunasundari  [211] developed a novel DL framework for multi-stage AD classification, representing a Parallel Integration approach (Fig. 13a). The methodology extracted features independently from pre-trained ViT and DenseNet-121 models to combine their complementary strengths. An ExtraTrees classifier was then used to select the most discriminative features from the combined set, and these reduced features were fed into a dense neural network for final 4-class classification (ND, VMD, MD, and MoD). The approach also incorporated data augmentation to handle dataset limitations and utilized various Class Activation Maps (CAMs) for visualizing model decisions and enhancing interpretability. The model was evaluated on a Kaggle MRI dataset expanded to 10,737 images via augmentation. However, the study’s validation methodology has several critical limitations. The evaluation was conducted exclusively on a single Kaggle dataset without external validation on an independent clinical cohort. Furthermore, the paper does not specify whether a subject-level data split was used, introducing a significant risk of data leakage that could lead to an overestimation of performance. The proposed fusion model achieved a superior accuracy of 99.0%, outperforming the individual ViT (93%) and DenseNet-121 (87%) models. This exceptionally high accuracy should be interpreted with considerable caution, as it was obtained on a heavily augmented, single-source dataset and may not be generalizable to real-world clinical data. The potential for data leakage and overfitting to the specific characteristics of the Kaggle dataset constrains the clinical relevance of these findings pending more rigorous validation.

**Fig. 29:**
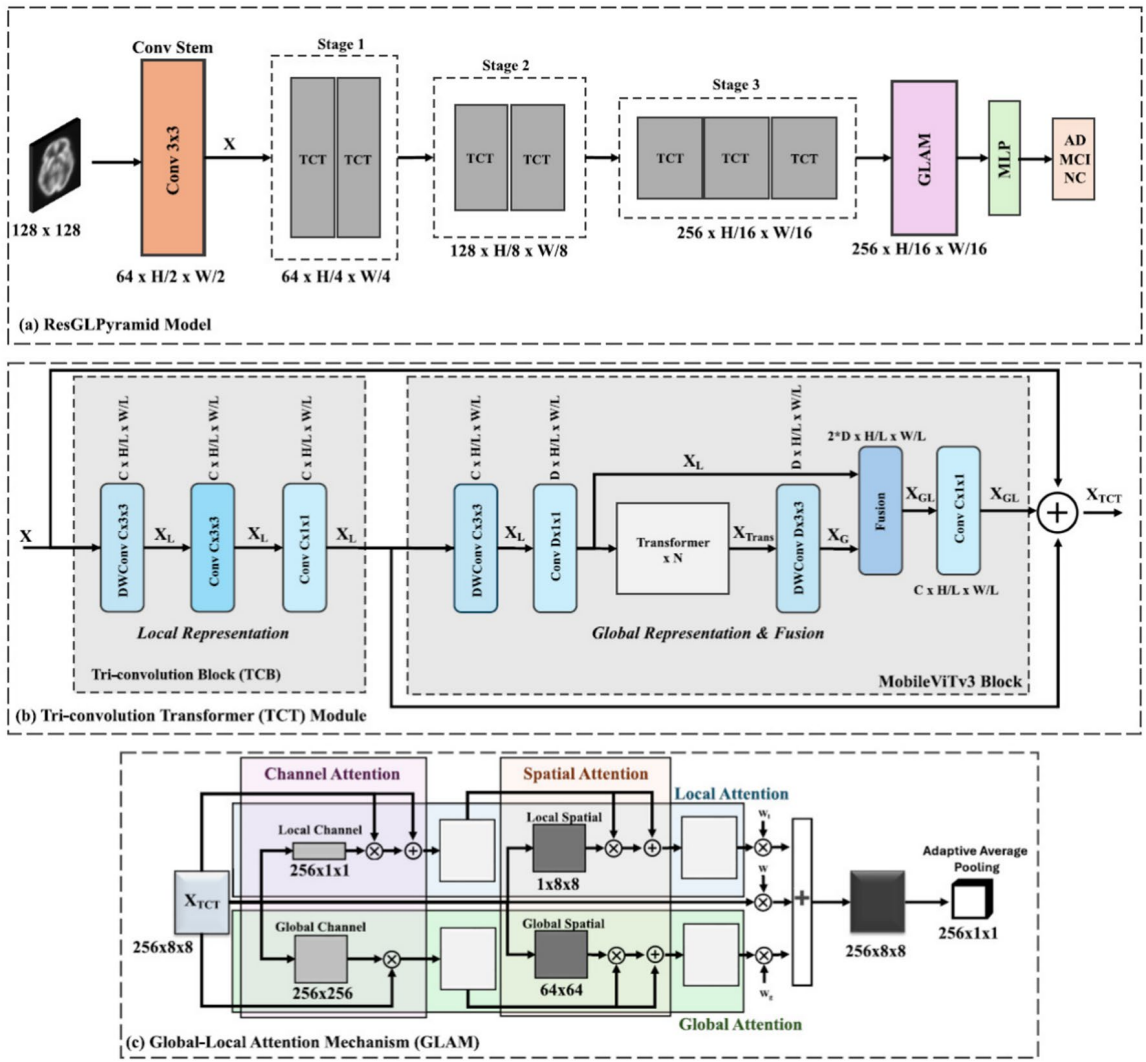
The ResGLPyramid model architecture. The framework integrates TCT modules for hybrid local–global feature learning and a GLAM for feature refinement. It is optimized for early AD diagnosis from PET scans using a softened cross-entropy loss. Figure adapted from [165]

*Second: the studies that employed 18F-FDG PET imaging for the diagnosis of AD:* In 2024, Rehman et al. [165] introduced ResGLPyramid (Fig. 29), a novel DL model for the early diagnosis of AD using ^18^F-FDG PET images. The model exemplified a Hierarchical Integration architecture (Fig. 13c) by deeply intertwining local and global feature extractors within its core Tri-Convolutional Transformer (TCT) modules. The MobileViTv3 block within these modules captured global dependencies using standard MHSA and a FFN:

41 $$\begin{aligned} X_{Trans}&= \text {MHSA}(\text {LN}(X_L(p)) + X_L(p)) \end{aligned}$$

42 $$\begin{aligned} X_{Trans}&= \text {FFN}(\text {LN}(X_{Trans}(p))) + X_{Trans}(p) \end{aligned}$$

The architecture was further enhanced with a Global–Local Attention Module (GLAM), which applied combined spatial and channel attention for final feature refinement. A key aspect of the model was its use of a softened cross-entropy (SCE) loss function, which combined the negative log-likelihood loss with a label smoothing term to improve generalization and mitigate overfitting on subtle metabolic changes:

43 $$\begin{aligned} \text {SCELoss} = (1 - \alpha ) \cdot \text {NLLLoss} + \alpha \cdot \text {SmoothLoss} \end{aligned}$$

The model was evaluated on the ADNI dataset via 10-fold cross-validation. While this protocol provides a robust measure of internal validity, the study’s findings are based exclusively on a single cohort. The absence of external validation on an independent, multi-center PET dataset is a significant limitation. ResGLPyramid achieved high performance in binary classification tasks, notably 92.75% accuracy for AD vs. MCI and 96.90% for AD vs. NC. Although these results demonstrate the potential of the architecture, its generalizability to PET data acquired with different scanners and protocols remains an open and critical question that must be addressed in future work.

*Third: the studies that employed fMRI imaging for the diagnosis of AD:* In 2023, Zuo et al. [212] introduced a novel Distribution-Regularized Adversarial Graph Autoencoder (DAGAE) featuring a transformer generator, designed to improve dementia diagnosis by augmenting brain functional network (BFN) datasets derived from fMRI. This model followed a Serial Integration pattern (Fig. 13b), where a graph encoder first processed the input BFNs, and a subsequent transformer-based generator mapped the latent representations to graph connections by capturing long-range dependencies. This process generated synthetic BFNs intended to preserve the essential topological characteristics and discriminative features found in real BFNs, thereby enriching limited datasets. The approach was validated on a small, balanced sub-cohort of the ADNI dataset, consisting of only 150 subjects (75 LMCI, 75 NC). The evaluation was performed using a 5-fold cross-validation protocol without testing on any external, independent dataset. The study found that training classifiers on a combination of original and generated data improved diagnostic performance across several binary classification tasks. However, the reliance on a very small, single-center cohort is a significant limitation. While the generative approach is innovative, the high classification accuracies achieved should be interpreted with caution, as the model’s ability to generalize to more diverse and larger patient populations remains unconfirmed. The performance gains may be specific to the characteristics of this limited dataset, and further validation is required to establish the robustness of the DAGAE framework.

Zhou et al.  [140] introduced LCGNet (Local sequential feature Coupling Global representation learning), a hybrid DL model for brain disease classification, whose architecture is best characterized as a Parallel Integration design, as described in Subsect. 4.4.2 and conceptually shown in Fig. 13a. The model first constructed dynamic functional connectivity networks (dFCNs) from rs-fMRI, where connectivity $F_t$ was computed via Pearson correlation:

44 $$\begin{aligned} F_t(i,j) = \text {corr}(x_i^t, x_j^t) \end{aligned}$$

**Fig. 30:**
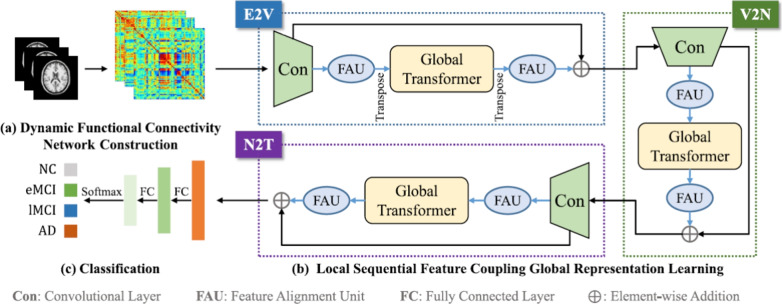
The LCGNet framework, a parallel CViT architecture for analyzing dynamic functional connectivity networks. It features a dual-branch backbone with a CNN stream for local feature extraction and a Transformer stream for global representation learning. A series of coupling components hierarchically fuse the information from both branches. Figure adapted from [140]

The LCGNet architecture (Fig. 30b) featured a dual-branch backbone with two concurrent streams: a CNN branch for extracting local sequential features and a global Transformer branch for capturing long-range temporal dependencies. The Transformer branch employed standard MHSA and Feed-Forward (FF) blocks:

45 $$\begin{aligned} Z= & \text {Softmax}\left( \frac{Q K^T}{\sqrt{D_k}}\right) V \end{aligned}$$

46 $$\begin{aligned} X_{out}= & \text {FF}(\text {LN}(X_{in} + Z)) + (X_{in} + Z) \end{aligned}$$

The fusion of these parallel streams was managed by three sequential coupling components that hierarchically merged the local and global topological information, utilizing a key Feature Alignment Unit (FAU) with $1 \times 1$ convolutions to ensure consistency between the branches. The model was evaluated on the ADNI dataset using a 5-fold cross-validation scheme. While this method ensures robust internal validation, the study’s evaluation of AD-related tasks was confined to this single cohort. LCGNet achieved 95.3% accuracy for AD vs. NC, 87.8% for eMCI vs. NC, and 66.7% for the four-class AD vs. LMCI vs. EMCI vs. NC classification. The absence of external validation on an independent rs-fMRI dataset is a key limitation, as the model’s ability to generalize across different scanners and patient populations has not been demonstrated. Therefore, the high reported accuracies should be considered in the context of this single-dataset evaluation.

In 2024, Zhang et al. [146] presented the Hierarchical Functional Brain Network (HFBN) model, a DL approach for early AD diagnosis (MCI vs. NC classification) leveraging rs-fMRI data. The model design represented a Hierarchical Integration strategy (Fig. 13c). It combined spatiotemporal graph convolutional feature extraction (a CNN-like operation on graphs) with a hierarchical node fusion module (HNFM) based on the transformer architecture, which interleaved spatiotemporal graph convolutions with transformer-based fusion. Key innovations included the adaptive construction of multi-scale functional brain networks using sparse attention (entmax) and soft node-merging layers to reflect brain hierarchy, and a sub-ROI generation method (using Farthest Point Sampling) to analyze functional interactions at finer scales than standard atlases permitted. A significant methodological strength of this study is its extensive validation across four distinct datasets (ADNI2, ADNI3, OASIS, and the private HUASHAN-MCI cohort), providing a robust assessment of the model’s generalizability. The HFBN model consistently outperformed state-of-the-art methods, achieving accuracies of 83.7% on ADNI2, 81.2% on ADNI3, 89.9% on OASIS, and 70.4% on the HUASHAN-MCI dataset for MCI vs. NC classification using 270 sub-ROIs derived from the AAL atlas. While the model’s superior performance across multiple cohorts is a notable achievement, the absolute accuracy scores highlight the persistent difficulty of early AD diagnosis from rs-fMRI. The architectural complexity, though powerful, also presents challenges for interpretability and clinical translation, underscoring that even with advanced hierarchical models, the problem remains far from solved.

#### Single-modality MCI to AD conversion prediction

This subsection focused on the application of CViT models, utilizing single-modality longitudinal sMRI, for the specific task of predicting the conversion from MCI to AD. The reviewed study explored how the hybrid nature of CViTs could capture spatio-temporal changes indicative of progression.

**Fig. 31:**
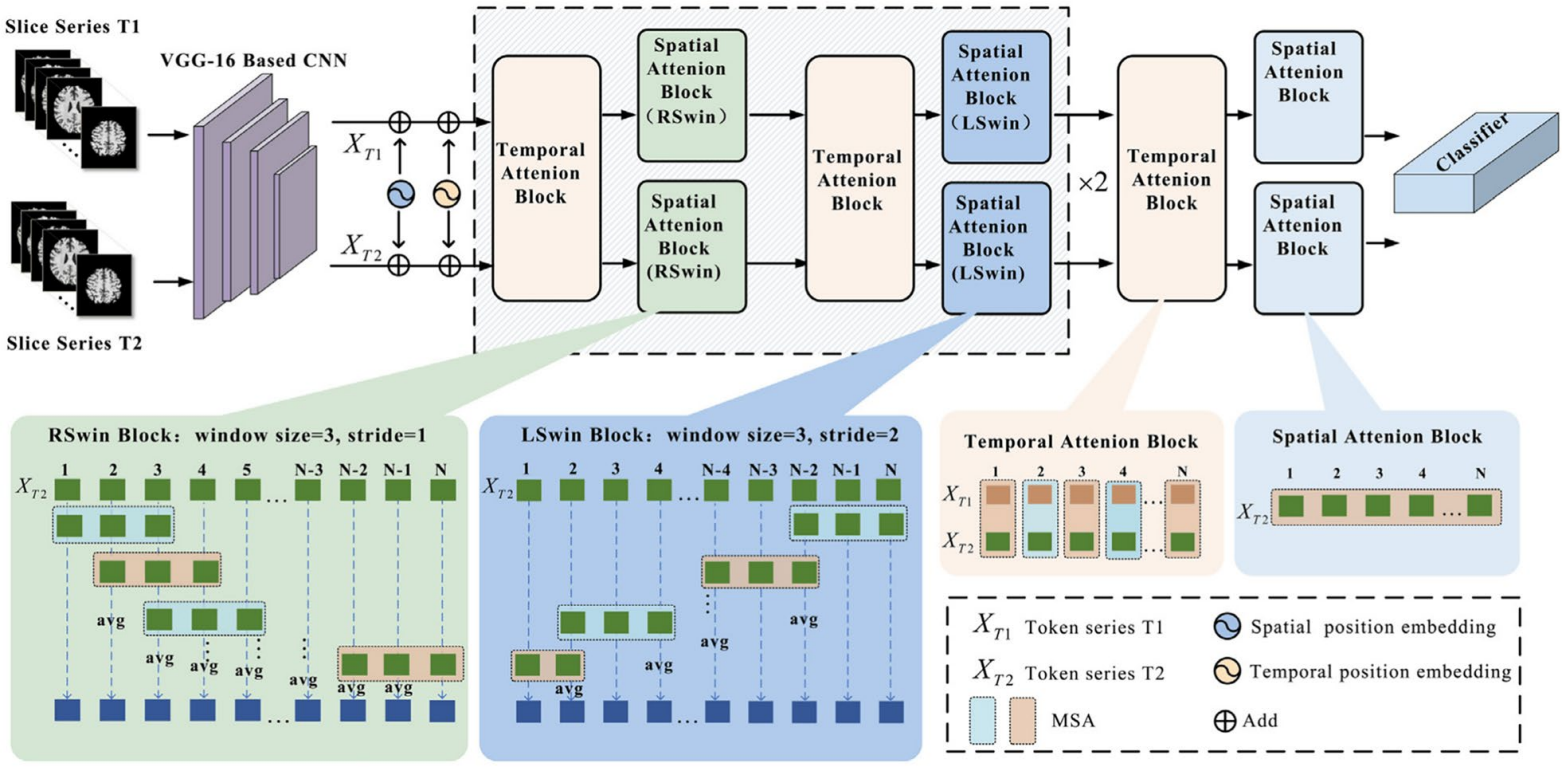
The VGG-TSwinformer architecture for longitudinal sMRI analysis. A VGG-16 CNN acts as a feature extractor on scans from two time points. A subsequent Transformer then uses a combination of spatial and temporal attention mechanisms to synergistically model both the spatial patterns within each scan and the temporal differences between them for MCI progression prediction. Figure adapted from [131]

In 2023, Hu et al. [131] proposed VGG-TSwinformer, a transformer-based model designed to predict MCI progression (sMCI vs. pMCI) by analyzing longitudinal sMRI data (Fig. 31). The architecture followed a Serial Integration design (Fig. 13b). Initially, a VGG-16-based CNN acted as a feature-extraction front-end, processing each 3D scan slice-by-slice to generate high-level feature token sequences for two time points ($X^{(l-1)}_{\text {T1}}$, $X^{(l-1)}_{\text {T2}}$), which were then augmented with positional encoding. These token sequences were fed into a Transformer architecture designed for spatio-temporal fusion. For its multimodal data combination (treating the two time points as modalities), it used a form of Intermediate Fusion (Temporal), as discussed in Subsect. 6.1.2. The Transformer was structured with standard layers:

47 $$\begin{aligned} t(x)&= \text {MHSA}(\text {LN}(x)) + x \end{aligned}$$

48 $$\begin{aligned} \text {Transformer}(x)&= \text {MLP}(\text {LN}(t(x))) + t(x) \end{aligned}$$

This architecture employed alternating spatial and temporal attention blocks. Spatial attention used sliding-window MHSA to model intra-timepoint relationships. The MHSA mechanism is defined as:

49 $$\begin{aligned} \text {head}_i&= \text {Attention}(QW_i^Q, KW_i^K, VW_i^V) \end{aligned}$$

50 $$\begin{aligned} \text {MHSA}(Q, K, V)&= \text {Concat}(\text {head}_1, ..., \text {head}_h)W^O \end{aligned}$$

Temporal attention was designed to capture longitudinal atrophy by performing cross-timepoint MHSA between spatially corresponding tokens from T1 and T2:

51 $$\begin{aligned} (X^l_{\text {T1},i}, X^l_{\text {T2},i}) = \text {MHSA}(X^{(l-1)}_{\text {T1},i}; X^{(l-1)}_{\text {T2},i}), \quad i = 1,...,N \end{aligned}$$

The final aggregated representation was passed to an MLP classifier. The model was evaluated on the ADNI dataset. However, the study’s validation was confined to this single cohort, with no external validation performed on an independent, multi-center dataset to assess the model’s generalizability. The VGG-TSwinformer achieved 77.2% accuracy, 79.97% sensitivity, 71.59% specificity, and an AUC of 0.8153 for this task, demonstrating improved diagnostic efficiency over cross-sectional methods. While the model’s ability to leverage longitudinal data is a significant contribution, these performance metrics should be considered in the context of a single-dataset evaluation; its robustness to variations in imaging protocols from other clinical sites remains an open question.

#### Single-modality AD classification and MCI conversion prediction

Subsection 8.1.3 addressed a study that employed a CViT-based model with single-modality sMRI data to tackle both AD classification and prediction of MCI-to-AD conversion within a unified framework. This approach leverages the strengths of CViTs for comprehensive diagnostic and prognostic assessment.

In 2023, Zhao et al. [32] introduced IDA-Net, an Inheritable Deformable Attention Network. The model’s architecture was a form of Hierarchical Integration (Fig. 13c). The architecture was organized into four sequential stages that formed a multi-scale feature pyramid, a hallmark of hierarchical design. Between each stage, the model utilized strided 3D convolutions to progressively downsample the spatial dimensions of the feature maps while increasing their channel depth. This process allowed the network to first capture fine-grained details at high resolution and subsequently learn more abstract, semantic features at coarser scales in the deeper layers. Operating within this hierarchical framework, the authors strategically embedded their novel 3D Deformable and Inheritable Self-Attention modules. This integration enabled the model to dynamically focus on and sample atrophic brain regions of varying sizes and locations, with the "inheritable" mechanism allowing this adaptive focus to be refined across the stages. The study’s methodology is strengthened by its validation on both the ADNI and AIBL datasets, with the latter serving as an independent external test cohort to assess model generalizability. This hierarchical approach proved effective, yielding 92.7% accuracy in AD classification and 83.5% accuracy in predicting MCI conversion on the ADNI test set. On the external AIBL dataset, the model demonstrated good, albeit slightly reduced, performance with 90.9% accuracy for AD classification. This modest performance drop underscores the persistent challenge of domain shift between clinical cohorts, but the model’s ability to maintain high accuracy on unseen data highlights the robustness of the proposed deformable attention mechanism.

### Applications of CViT with multimodality

Subsection 8.2 detailed 26 studies that utilized CViT architectures in conjunction with multimodal data inputs. This approach sought to harness the complementary information from diverse sources, such as neuroimaging, clinical, and genetic data, to enhance diagnostic and prognostic capabilities. The reviewed literature was organized based on the primary diagnostic objective: AD classification (Subsect. 8.2.1), MCI-to-AD progression prediction (Subsect. 8.2.2), and studies addressing both (Subsect. 8.2.3).

#### Multimodal AD classification

This subsection examined 19 studies that applied CViT models to multimodal data for AD classification. The investigations covered various combinations, including sMRI with clinical data, sMRI with genetic data, and more comprehensive fusions involving sMRI, PET, and CSF biomarkers, highlighting the versatility of CViTs in integrating heterogeneous information.

Dai et al.  [102] proposed DE-JANet, a unified architecture for multimodal AD classification integrating sMRI with clinical data (age and MMSE score). To address the limitations of standard ViTs in capturing fine-grained spatial details, this hybrid design represented a Serial Integration architecture, a structure depicted in Fig. 13b and discussed in Subsect. 4.4.2. In the first stage, it employed a 3D CNN encoder to extract local spatial features from sMRI, while a separate linear encoder with sinusoidal mapping captured magnitude information from age and MMSE. For data integration, the framework utilized an Intermediate (Single-Level) fusion strategy (Fig. 14b), a method discussed in Subsect. 6.1.2. This was achieved in the second stage within a ViT-inspired Joint Attention Module that concatenated tokens from both sMRI and clinical data and processed them jointly in a Transformer layer, modeling their interactions to generate a unified global representation. A key methodological strength of this study is its rigorous validation protocol; the framework was trained on the ADNI-1 dataset and then tested for generalization on the independent ADNI-2 cohort. This approach provides a robust assessment of the model’s performance on unseen data from a different study phase, which may involve variations in imaging protocols and patient populations. The framework achieved high mean accuracies of 97.22% for AD vs. NC and 95.38% for MCI vs. NC classification. The model’s ability to maintain high accuracy on an external dataset, particularly for the challenging MCI vs. NC task, underscores the effectiveness of its dual-encoder and joint-attention fusion mechanism in learning generalizable, cross-cohort biomarkers.

**Fig. 32:**
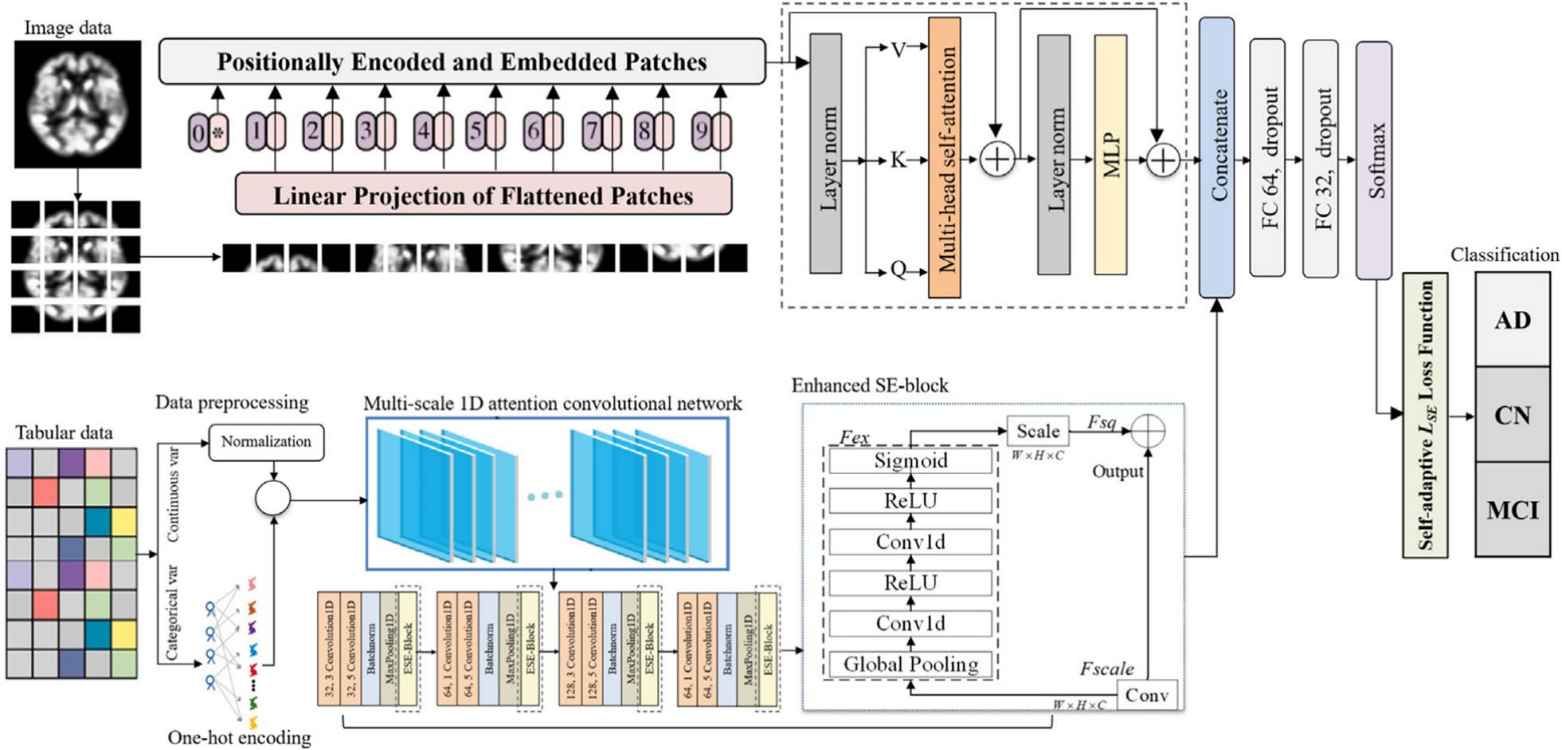
The architecture of the MMDF model, a parallel framework for multimodal AD diagnosis. The model simultaneously processes sMRI scans with a ViT and clinical data with a specialized 1D CNN featuring multi-scale attention. The high-level representations from these two independent streams are then integrated to make a final classification. Figure adapted from [142]

Furthermore, in 2025, Chen et al. [142] proposed the Multimodal Mixing CNN and Transformer (MMDF) for AD classification using sMRI and clinical data (Fig. 32). The model’s design followed a Parallel Integration architecture, as described in Fig. 13a, involving two concurrent processing streams. First, an MRI_ViT module learned global spatial features from MRI scans by processing image patches through a standard Transformer encoder, where features $z_l$ at layer *l* were derived from the previous layer $z_{l-1}$:

52 $$\begin{aligned} z'_l&= \text {MHSA}(\text {LN}(z_{l-1})) + z_{l-1} \end{aligned}$$

53 $$\begin{aligned} z_l&= \text {MLP}(\text {LN}(z'_l)) + z'_l \end{aligned}$$

Concurrently, a parallel multi-scale attention-embedded one-dimensional CNN (MA-1DCNN) extracted informative patterns from clinical records. A key feature of this sub-model was its Enhanced Squeeze-and-Excitation (ESE) block for adaptive feature recalibration, which reweighted feature maps $u_k$ using a learned scaling factor $s_k$:

$$ \widetilde{X}_k = s_k \cdot u_k $$

To combine the outputs of these branches, the framework utilized an Intermediate (Single-Level) fusion strategy, as described in Fig. 14b. To mitigate class imbalance and noise interference, the framework was trained with a self-adaptive hybrid loss function that dynamically combined Symmetric Cross Entropy (SCE) and an enhanced Focal Loss (EFL):

$$ \mathcal {L}^{(t)} = w_1^{(t)} \mathcal {L}_{SCE}^{(t)} + w_2^{(t)} \mathcal {L}_{EFL}^{(t)} $$

The model was evaluated on the ADNI dataset, using a fixed 70%-20%-10% split for training, validation, and testing. A key limitation of this validation approach is its confinement to a single data source, as no external validation was performed on an independent cohort. The MMDF achieved 97.65% accuracy, 96.40% recall, and a 96.69% F1-score. The authors noted that the combination of hierarchical convolutional features from the MA-1DCNN and global attention mechanisms from the ViT enabled superior AD detection compared to unimodal architectures. While these results are strong, the absence of testing on a non-ADNI dataset means the model’s generalizability to different clinical environments and imaging protocols remains an unverified but critical question for future work.

*The following study used genetic data with sMRI and clinical data for AD classification.* Liu et al. (2024) [192] proposed the Sparse Transformer Association Analysis (STAA) framework, a unified DL model for brain imaging genetics association analysis, specifically applied to AD diagnosis and brain age regression using sMRI and genetic (SNP) data. The model’s architecture was a form of Serial Integration, as described in Subsect. 4.4.2. In its initial stage, it employed a convolutional autoencoder and a novel sparse transformer (s-transformer) decision network, guided by an L1 sparsity penalty, to extract and select salient phenotypic features from MRI scans at the voxel level. For multimodal integration, the framework utilized a Hybrid (Generative) fusion approach, as detailed in Subsect. 6.1.4. A key component was a cross-modal generative network, featuring another s-transformer, which modeled the association between identified genetic variants and imaging features. This enabled the generation of neuroimages conditioned on genetic data and voxel-level association analysis. The STAA framework was evaluated on simulated and real ADNI datasets. While the use of simulated data effectively validates the model’s theoretical components, the clinical validation was confined to a moderately sized cohort from the ADNI database, with no external validation on an independent clinical dataset. The model achieved higher performance in AD diagnosis (AUC 0.920 for MRI, 0.711 for SNP) and brain age prediction (MAE 3.15 for MRI, 1.51 for SNP) compared to established methods. It also successfully identified known AD-related MRI regions and SNPs, such as APOE4. These promising results highlight the potential of the sparse transformer for biomarker discovery, but the findings’ generalizability is limited by the single-cohort evaluation. Further validation on larger, multi-ethnic datasets is necessary to confirm the robustness of the identified imaging-genetic associations.

*In the following studies, 11 studies use the sMRI with PET for AD classification.*

Building on their previous work with single-modality data [153], Kadri et al.  [178] introduced another hybrid DL framework for early AD detection, this time utilizing both MRI and PET neuroimaging data. Their proposed multimodal approach followed a Serial Integration architecture, as explained in Subsect. 4.4.2, integrating an EfficientNetV2 for robust local feature extraction with a ViT for modeling long-range spatial dependencies. For data integration, it employed an Input-Level fusion strategy, as described in Fig. 14a and Subsect. 6.1.1, by first fusing the imaging modalities before feeding them into the main network. A distinctive aspect of their methodology was the enhancement of the training process through a novel data augmentation technique based on Self-Attention Generative Adversarial Networks (SAGAN) to improve model generalization. The combined model was validated using the ADNI and OASIS datasets, reporting a high classification accuracy rate of 96.0%. However, the study lacks critical details regarding its validation methodology. Key information–such as the specific use of the ADNI and OASIS cohorts (e.g., for training versus external testing), data splitting ratios, and whether subject-level partitioning was enforced to prevent data leakage–is not provided. This methodological opacity makes it difficult to independently assess the risk of overfitting or the true generalizability of the model. Therefore, while the reported accuracy is high, it should be interpreted with caution pending a more transparent and reproducible validation framework.

Tang et al.  [156] proposed the CsAGP, a dual-transformer model for AD classification using multimodal neuroimaging data. The model architecture combined a Serial Integration pattern with Cross-Attention, with these techniques detailed in Subsect. 4.4.2. For its data fusion, it implemented an Intermediate (Attention-Based) fusion strategy, a method depicted in Fig. 14d and explained in Subsect. 6.1.2. It featured a cross-attention fusion module (CAFM) to enhance feature interactions between sMRI and PET scans, leveraging self-attention to capture global and modality-specific representations. A graph pooling-based Reshape-Pooling-Reshape (RPR) framework was also employed to reduce token redundancy, improving computational efficiency while preserving critical diagnostic information. CsAGP was trained and evaluated on the ADNI dataset. However, the validation was conducted using a simple percentage-based data split within this single cohort, without testing on an independent external dataset. The paper also does not explicitly confirm that the data split was performed at the subject level, which is a critical detail for ruling out potential data leakage. The model achieved exceptionally high accuracies, including 99.04% for AD vs. CN, 97.43% for AD vs. MCI, and 98.72% for the multi-class AD vs. CN vs. MCI task. While these results highlight the power of the novel cross-attention and graph-pooling architecture, the lack of external validation and ambiguity in the data partitioning strategy mean the reported performance may be an overestimation of the model’s true generalizability. Further validation on independent, multi-center cohorts with a strictly enforced subject-level split is necessary to confirm these findings.

*The following four studies by Odusami et al. consistently explored variations of an Input-Level (Signal-Level) fusion methodology, where sMRI and PET scans were first merged into a single image before being processed by a serial CViT architecture, primarily a Mobile ViT.*

First, in 2023, Odusami et al. [148] introduced a pixel-level fusion approach for early AD detection from multimodal neuroimaging data. The model architecture followed a Serial Integration design, as described in Fig. 13b. For data fusion, it used an Input-Level fusion strategy (Fig. 14a, Subsect. 6.1.1), integrating a Discrete Wavelet Transform (DWT) to fuse MRI and PET scans at the pixel level before feature extraction. A VGG16 model was then used for feature extraction from these fused images, and a pre-trained ViT was fine-tuned for the final classification. This approach leveraged the ViT’s self-attention mechanisms to enhance the combined structural and functional feature representation, aiming to capture complementary information that single-modal models might overlook. The model was trained and evaluated on the ADNI dataset for AD vs. EMCI and AD vs. LMCI classification tasks. However, the study’s validation suffers from significant methodological limitations that temper its conclusions. The final evaluation was conducted on an extremely small test set of only 16 samples per task, making the reported percentage-based accuracies statistically fragile and highly sensitive to cohort selection. The study reported accuracies of 81.25% on MRI test data and 93.75% on PET test data. This unconventional reporting–evaluating a fused model on single-modality test data–adds a layer of ambiguity. Given the very small cohort and unclear validation protocol, the results should be considered preliminary and do not provide sufficient evidence for the model’s generalizability or robustness.

Odusami et al.  [149] introduced an optimized convolutional fusion method designed to enhance multimodal neuroimaging analysis for AD diagnosis. The proposed technique represented a Serial Integration architecture, as described in Subsect. 4.4.2, and its data integration followed an Input-Level fusion approach (Subsect. 6.1.1). The method leveraged optimized transposed convolution, incorporating varying kernel sizes and instance normalization, applied to features initially processed with Laplace sharpening and derived from a VGG19 backbone. A Maximum Fusion strategy was subsequently used to combine the feature maps from MRI and PET. The quality of the resulting fused images was evaluated on the ADNI, OASIS, and AANLIB datasets using metrics such as PSNR and SSIM, demonstrating superior performance compared to traditional methods like DWT and LPG. The diagnostic utility of these fused images was then assessed by using Mobile ViTs (MViT) for downstream classification. While the use of three distinct datasets is a methodological strength, the validation was conducted on very small cohorts, with only 50 participants per class drawn from each database. The study does not specify the train/test splitting ratio for the classification task, making it difficult to assess the results critically. The model achieved high accuracy (e.g., 99.00% for AD vs. CN on the AANLIB dataset). However, this near-perfect accuracy should be interpreted with significant caution, as it was likely achieved on a test set comprising a very small number of images. Such results on small cohorts are statistically fragile and may not be representative of the model’s true performance or its ability to generalize to larger, more diverse patient populations.

**Fig. 33:**
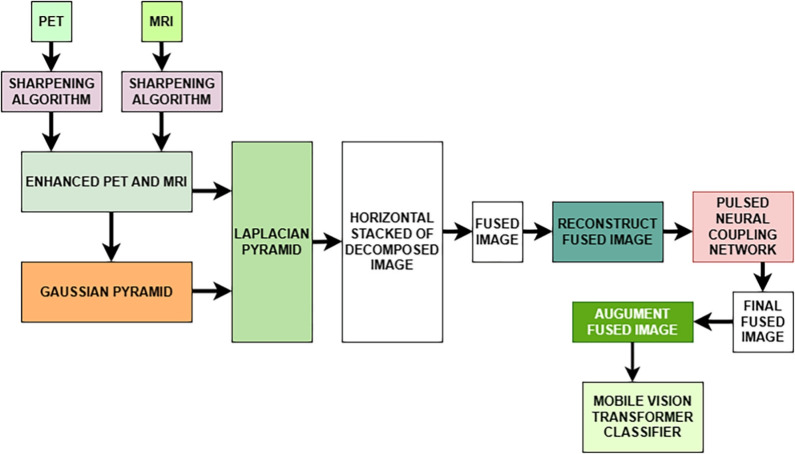
The multimodal AD diagnostic pipeline. The framework employs a sophisticated Input-Level fusion technique, combining GLP decomposition with a SPCNN to merge sMRI and PET scans. The resulting fused and reconstructed image is then classified using an optimized MViT. Figure adapted from [151]

In 2024, Odusami et al. [151] introduced a multimodal DL framework for AD stage recognition (Fig. 33). Their approach followed a Serial Integration architecture, a design described in Subsect. 4.4.2. For data integration, it used an Input-Level (Spatial) fusion method, as explained in Subsect. 6.1.1. The process began by enhancing sMRI and FDG-PET images with a Laplacian kernel:

54 $$\begin{aligned} \text {Image}'(n,m) = \text {Image}(n,m) - [H_L(n,m) * \text {Image}(n,m)] \end{aligned}$$

where $H_L$ is the Laplacian kernel. These enhanced images were then decomposed using Gaussian/Laplacian Pyramids (GLP). The corresponding Laplacian pyramid levels were fused via a Simplified Pulse Coupled Neural Network (SPCNN), which iteratively updates pixel values based on neighboring activity:

55 $$ \begin{aligned} a(k,l) = & m(k,l) \\ \quad & + \beta \cdot [b(k - 1,l) + b(k + 1,l) + b(k,l - 1) + b(k,l + 1)] \\ \end{aligned} $$

56 $$\begin{aligned} b_{\text {new}}(k,l)&= b_{\text {old}}(k,l) \cdot \exp (a) + S(k,l) \cdot [1 - \exp (a)] \end{aligned}$$

The reconstructed fused images were subsequently used to train a Mobile ViT (MViT). The MViT’s architecture, which incorporated multiscale feature hierarchies and specialized attention mechanisms, was optimized using a Pareto-Optimal Quantum Dynamic Optimization (QDO) algorithm to balance performance and complexity. The model, trained on fused images, was then unconventionally evaluated on small, independent test sets of unfused single-modality data from ADNI and OASIS. This method achieved high accuracy for binary classifications on the ADNI MRI test data, such as 94.73% for (AD vs. CN), 92.98% for (AD vs. MCI), and 89.36% for (CN vs. MCI). However, the number of subjects contributing to these test sets is not clearly specified, and evaluating a fusion-trained model on single-modality data raises questions about the validation strategy. The statistical robustness of these high-performance figures is therefore difficult to ascertain, and the model’s generalizability remains unconfirmed without a more conventional external validation on a larger, independent cohort using the same data modality as the training process.

Also, in 2024, Odusami et al. [150] presented a multimodal fusion approach using image colorization and a Mobile ViT (MViTv3) for AD stage classification. The method implemented a Serial Integration architecture, as described in (Fig. 13b, Subsect. 4.4.2). For data integration, it used an Input-Level fusion strategy (Fig. 14a, Subsect. 6.1.1), first fusing sMRI and FDG-PET images using an optimized transposed convolution technique. Subsequently, a Pareto-optimized cosine color map was applied to the fused grayscale images to enhance visual features and interpretability. An MViTv3 model, incorporating the Swish activation function, was then used for binary classification across different AD stages (AD, CN, EMCI, LMCI). The model was trained and evaluated using fused and colorized images derived from the ADNI, OASIS, and AANLIB datasets. Methodologically, the study relied on massive data augmentation to expand its small initial cohorts before partitioning into training and testing sets. While this is a common technique, it introduces a risk of the model overfitting to the specific augmentation strategy rather than learning generalizable biological features. The study reported exceptionally high accuracies on ADNI data, such as 99.25% for AD vs. CN and 98.76% for EMCI vs. LMCI. These near-perfect results, however, should be interpreted with significant caution. The validation was performed on a small hold-out set derived from the augmented data pool, and the study lacks a true external validation on an independent, non-augmented cohort. The statistical robustness of such high accuracies on small test sets is questionable, and the model’s ability to generalize to real-world clinical data remains unconfirmed.

Khan et al.  [182] introduced Dual-3DM^3^-AD, a multimodal framework for the early and accurate multi-class AD diagnosis using MRI and PET scans. The model’s architecture was a form of Hierarchical Integration, a design illustrated in Fig. 13c and described in Subsect. 4.4.2. It integrated a Mixed Transformer with a Furthered U-Net for advanced semantic segmentation, enabling precise feature extraction from preprocessed neuroimaging data. For data integration, it used an Intermediate (Hierarchical) fusion approach, a strategy shown in Fig. 14c and explained in Subsect. 6.1.2. A multi-scale feature extraction module captured relevant patterns, and the Densely Connected Feature Aggregator Module (DCFAM) fused information from both MRI and PET modalities. A multi-head attention mechanism was then applied to reduce feature dimensionality before classification using a softmax layer. The model was trained and evaluated on the ADNI dataset. However, the validation was conducted on a small cohort of 300 subjects using a 10-fold cross-validation protocol, with no mention of a final, held-out test set or any form of external validation on an independent dataset. The study reported high binary classification accuracies of 98.3% (AD vs. CN), 98.0% (CN vs. MCI), and 98.6% (AD vs. MCI). While the sophisticated architecture is a notable contribution, these high-performance figures should be interpreted with caution. The combination of a small cohort and the lack of a separate test set introduces a significant risk of overfitting and may not be indicative of the model’s true performance on unseen clinical data.

**Fig. 34:**
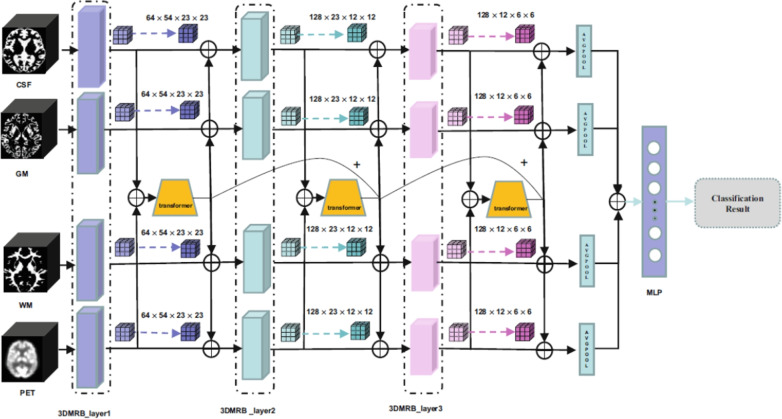
The architecture of the MMTFN model. The framework utilizes 3DMRB for initial feature extraction from each neuroimaging modality. A subsequent Transformer module then fuses these multi-scale features by modeling their inter-dependencies for a final, unified AD diagnosis. Figure adapted from [147]

In 2024, Miao et al. [147] introduced the MMTFN, a DL framework (Fig. 34) for AD classification using sMRI-derived (GM, WM, CSF) and PET neuroimages. The MMTFN framework represented a Hierarchical Integration architecture, as illustrated in Fig. 13c. It initially processed each modality through a series of 3D Multi-scale Residual Blocks (3DMRB), which employed parallel convolutions with varied expansion rates, whose outputs ($X'_i$) were summed to capture diverse feature patterns:

$$ X_{out} = \sum _{i=1}^{4} X'_i $$

Subsequently, a Transformer-based fusion module integrated these uni-modal features. This represented an Intermediate (Hierarchical) fusion strategy, as depicted in Fig. 14c. The module leveraged self-attention to capture global, long-range dependencies and construct a comprehensive joint representation:

$$ Att = \text {softmax}\left( \frac{S_Q S_K^T}{\sqrt{D_k}}\right) S_V $$

The final output was passed to an MLP for classification. The framework was evaluated on the ADNI dataset (720 subjects) using a 5-fold cross-validation protocol. While this method provides robust internal validation, the evaluation was confined to this single cohort, with no testing on an independent external dataset to assess the model’s performance on data from different clinical sites or scanners. This framework achieved a high accuracy of 94.61% in distinguishing AD from NC cases. These strong results underscore the potential of the multi-scale fusion architecture, but the lack of external validation means that the model’s generalizability remains unconfirmed. Further testing on diverse, multi-center cohorts is a necessary next step to verify its clinical applicability.

Furthermore, Tang et al.  [181] introduced an advanced multimodal diagnostic framework. The architecture followed a Serial Integration pattern, a design described in Fig. 13b and Subsect. 4.4.2. It synergistically integrated 3DCNNs for feature extraction from sMRI and PET scans, while an enhanced transformer progressively learned global correlations among the extracted features. For data integration, it used an Intermediate (Hierarchical) fusion strategy, a method shown in Fig. 14c and explained in Subsect. 6.1.2. This fusion of multimodal imaging data enabled a more comprehensive representation of AD-related brain abnormalities. The model was trained and evaluated on the ADNI dataset using a 10-fold cross-validation protocol on a cohort of 210 subjects. While this internal validation is robust, the study did not employ an independent, external dataset to assess the model’s generalizability to different clinical populations or imaging protocols. The model achieved an exceptionally high accuracy of 98.1% in distinguishing AD patients from CN individuals. Comparative analysis demonstrated that this multimodal approach significantly outperformed single-modality models, with sMRI-only and PET-only models achieving 91.91% and 87.14% accuracy, respectively. However, the near-perfect accuracy on a single, moderately-sized cohort, combined with the lack of external validation, suggests that the reported performance may be an overestimation of the model’s real-world utility and that further testing on diverse, multi-center data is required to confirm these promising results.

Another related approach, Qiu et al.  [193] introduced MDL-Net, a 3D DL network designed for early AD diagnosis by leveraging multimodal neuroimaging data, specifically sMRI (processing GM and WM separately) and PET scans. The architecture was a form of Hierarchical Integration, a design illustrated in Fig. 13c and described in Subsect. 4.4.2. It incorporated a Multi-fusion Joint Learning (MJL) module that combined global-aware learning (GAL) using a self-adaptive transformer, local-aware learning (LAL) with convolutions, and outer latent-space learning (LSL) via outer products to effectively fuse features from different perspectives. For data integration, the framework employed a Hybrid (Systemic) fusion approach, a method detailed in Subsect. 6.1.4, which also integrated a Disease-induced Region-aware Learning (DRL) component to incorporate brain ROI information, iteratively learning weights to identify AD-relevant regions and enhance interpretability. The model was evaluated on the ADNI and AIBL datasets. The primary performance was assessed via a robust 10-fold cross-validation within the ADNI cohort, while the AIBL dataset was used for separate cross-dataset validation to test generalizability. The model demonstrated strong performance on ADNI, achieving accuracies of 96.37% (AD vs. CN), 73.61% (MCI vs. CN), and 85.29% (AD vs. MCI), surpassing several comparison methods. While the use of a second cohort for cross-dataset testing is a significant methodological strength, the headline performance metrics are derived from the internal validation on ADNI. The slight performance degradation observed on the external AIBL cohort, though still strong, suggests that the complex architecture may be partially overfitted to the ADNI data characteristics, highlighting the ongoing challenge of achieving full generalizability across diverse clinical datasets.

**Fig. 35:**
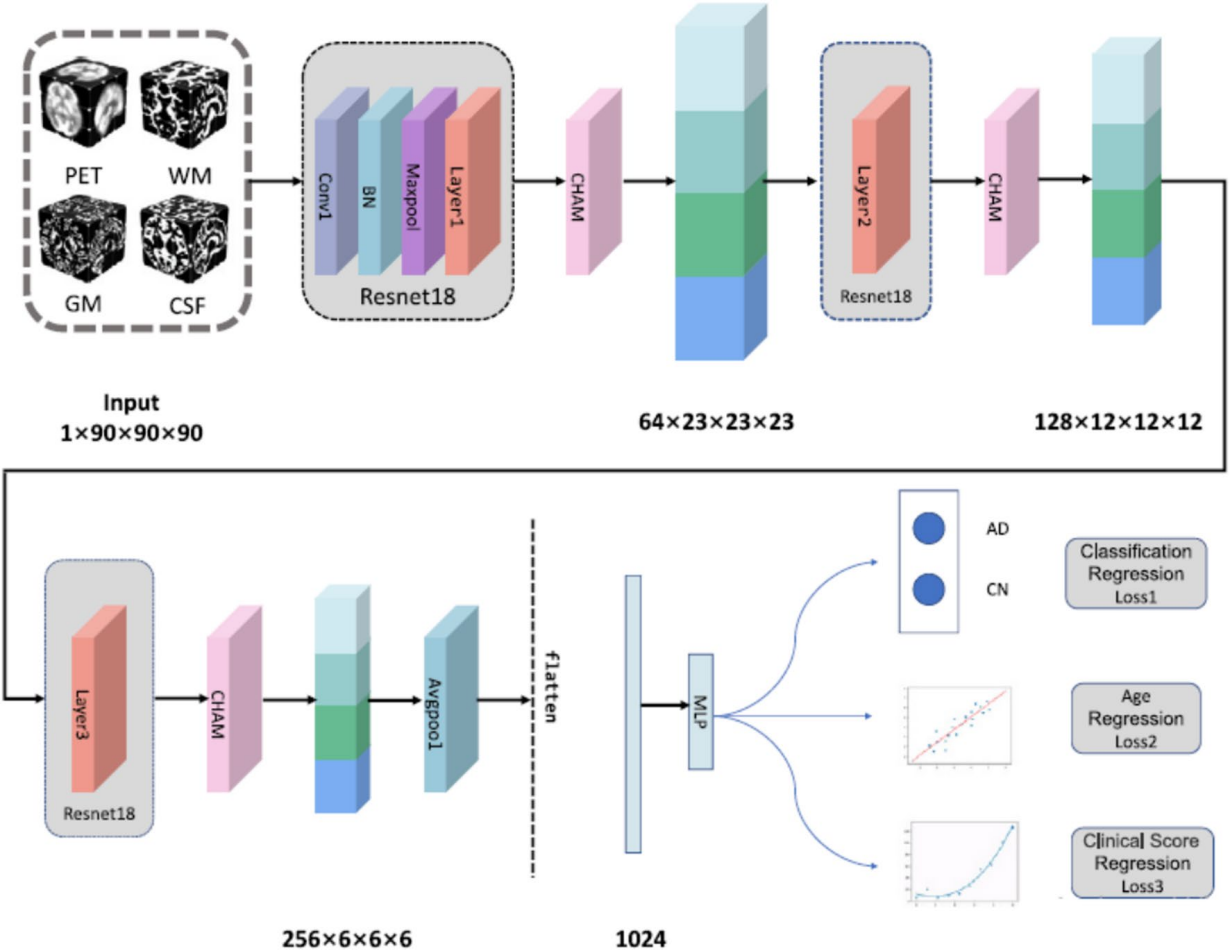
The architecture of the HAMMF model for multimodal AD diagnosis. The model introduces a CHAM and a Transformer to effectively fuse features from sMRI and PET. A key aspect of the framework is its multi-task learning head, which simultaneously performs AD classification while also regressing for age and MMSE score to improve feature learning and generalization. Figure adapted from [154]

Liu et al.  [154] introduced the HAMMF model, a DL framework (Fig. 35) processing sMRI and PET volumes. The HAMMF design was a Serial Integration architecture enhanced with Combined Channel and Spatial Attention, with these techniques described in Fig. 13b, h and detailed in Subsect. 4.4.2. It first employed a modality-specific feature extraction stage using a ResNet18 backbone to independently process sMRI-derived (Gray Matter (GM), White Matter (WM)), CSF, and PET volumes. For data integration, it used a Hybrid (Systemic) fusion approach, a method detailed in Subsect. 6.1.4. A novel Contextual Hierarchical Attention Module (CHAM) fused these multimodal features using channel and spatial attention, followed by a Transformer module that modeled inter-modal dependencies with scaled dot-product attention:

$$ \text {Att} = \text {softmax} \left( \frac{S_Q S_K^T}{\sqrt{D_k}} \right) S_V $$

A multi-task learning head then utilized the fused representation to concurrently perform AD/NC classification (primary task) and regress subject age and MMSE scores (auxiliary tasks). The overall model was trained by minimizing a weighted combination of these losses:

$$ L_{\text {total}} = L_c + \lambda _1 L_{r1} + \lambda _2 L_{r2} $$

The model was evaluated on the ADNI dataset (720 subjects) using a 5-fold cross-validation protocol. While this ensures a robust internal validation, the study’s evaluation was confined to this single cohort, with no testing on an independent external dataset to assess the model’s performance on data from different clinical environments. HAMMF achieved 93.15% accuracy for AD vs. NC classification, with ablation studies confirming the efficacy of both the CHAM and the multi-task learning paradigm. These strong results highlight the potential of combining multi-task learning with hierarchical attention, but the model’s generalizability remains unconfirmed. Further validation on diverse, multi-center cohorts is a critical next step to verify its clinical applicability.

**Fig. 36:**
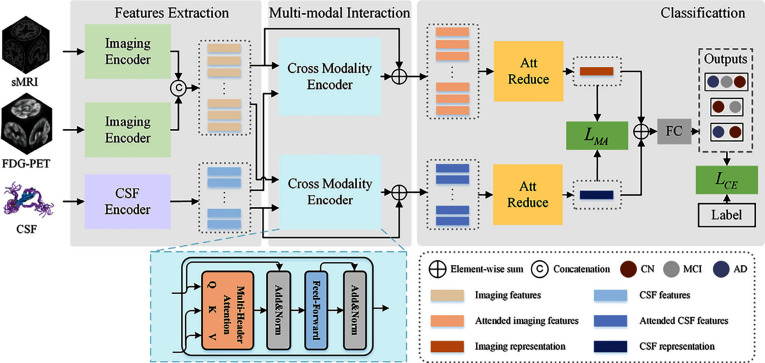
The architecture of the MCAD model for fusing imaging and biomarker data. After processing inputs with modality-specific encoders, the model uses a central MI Module. This module is built upon a cross-attention mechanism, where features from one modality (e.g., imaging) attend to features from another (e.g., CSF biomarkers) to create a deeply integrated and synergistic representation for AD classification. Figure adapted from [155]

*This study uses CSF biomarkers with sMRI and PET for AD classification:* Zhang et al.  [155] proposed the multimodal Cross-Attention Network (MCAD) for AD classification using sMRI, PET, and CSF biomarkers (Fig. 36). The framework implemented a Serial Integration architecture enhanced with Cross-Attention, with these strategies described in Subsect. 4.4.2 and Subsect. 4.4.2. In this pipeline, it first employed distinct feature encoders: an Imaging Encoder used Cascaded Dilated Convolution (CDC) modules to extract multi-scale features, while a CSF Encoder processed biomarkers via sinusoidal encoding. For data integration, it then used an Intermediate (Attention-Based) fusion strategy, a method depicted in Fig. 14d and explained in Subsect. 6.1.2. This was implemented in a multimodal Interaction (MI) Module, where each modality’s representation queried the other in symmetrical branches to produce enhanced, mutually aware representations, such as the non-imaging-aware imaging representation ($I^*$):

57 $$\begin{aligned} \hat{I}_{\text {intermediate}}&= \text {LN}(I + \text {MCA}(I,C)) \end{aligned}$$

58 $$\begin{aligned} I^*&= \text {LN}(\hat{I}_{\text {intermediate}} + \text {FFN}(\hat{I}_{\text {intermediate}})) \end{aligned}$$

The model was trained with a composite loss function combining cross-entropy with a modality alignment loss ($L_{MA}$) to reduce inter-modal feature discrepancy using the Kullback–Leibler (KL) divergence:

59 $$\begin{aligned} L_{\text {MA}} = D_{\text {KL}}(\hat{I}||\hat{C}) + D_{\text {KL}}(\hat{C}||\hat{I}) \end{aligned}$$

After an attention reduction step, a classification module concatenated the final features for prediction. The model was evaluated on the ADNI dataset using a 5-fold cross-validation protocol on a cohort of 467 subjects. While this internal validation is robust, the study did not employ an independent, external dataset to assess the model’s generalizability. MCAD achieved accuracies of 91.07% (AD vs. CN), 71.26% (MCI vs. CN), and 64.03% for the challenging multi-class (AD/MCI/CN) task. The strong performance in the AD vs. CN task highlights the potential of the cross-attention fusion mechanism; however, the more modest accuracy on MCI classification, combined with the single-cohort evaluation, suggests that further validation on larger, multi-center datasets is necessary to confirm the clinical applicability and robustness of this advanced fusion strategy.

Building on their previous work [153, 178], Kadri et al.  [169] proposed two novel hybrid DL architectures for AD diagnosis using multimodal neuroimaging data (MRI, PET, and CT). The first method, a Parallel Integration architecture (Fig. 13a), integrated a Swin Transformer with an enhanced EfficientNet, incorporating multi-head attention and a Depthwise Over-Parameterized Convolutional (DO-Conv) layer to capture both global dependencies and refined local features. The second method, a Serial Integration architecture (Fig. 13b), modified the CoAtNet architecture by replacing the standard Squeeze-and-Excitation (SE) block with an Efficient Channel Attention (ECA-Net) module and adding improved fused MBConv blocks to enhance efficiency. For data fusion, both models employed an Intermediate (Single-Level) fusion approach, a method detailed in Fig. 14b and Subsect. 6.1.2. The models were evaluated on the OASIS and ADNI datasets. However, the paper suffers from a significant lack of methodological transparency, as it does not provide details on the data splitting strategy, cohort sizes, or the specific validation protocol used. The modified CoAtNet achieved the highest unimodal accuracy: 97.33% on OASIS for four-class classification and 98.87% on ADNI for ternary (AD vs. CN vs. MCI) classification using MRI. Multimodal fusion of MRI and PET with the modified CoAtNet yielded the best overall performance, reaching 99.42% accuracy on ADNI for the same ternary classification task. While these near-perfect accuracies are striking, the absence of a clear and reproducible validation framework makes it difficult to assess their validity. Without information on crucial steps like subject-level partitioning to prevent data leakage, these results should be interpreted with significant caution as they may not be representative of the models’ true generalizability.

Furthermore, in 2025, Kadri et al. [188] unveiled a transformer-based hybrid model and an adaptive MLP-Mixer for AD detection using four imaging modalities (MRI, PET, DTI, and sfMRI). The primary transformer model followed a Serial Integration architecture (Fig. 13b), merging adaptive sparse self-attention with multi-head dilated self-attention to efficiently capture both nearby and far-reaching brain region connections. The adaptive MLP-Mixer was designed to boost spatial and cross-channel feature analysis while avoiding the complexity of traditional attention methods. For data integration, the study employed a Hybrid (Multi-Level System) fusion approach, as described in Subsect. 6.1.4. This involved testing various strategies. Methodologically, while the paper explores a comprehensive set of fusion techniques across multiple modalities from the ADNI and OASIS datasets, it suffers from a critical lack of transparency regarding its experimental setup. The study does not provide essential details on cohort size, data splitting, or the specific validation protocols used to derive its performance metrics. It reports that mid-level fusion of MRI and DTI achieved 96% accuracy, late fusion of MRI and sfMRI reached 98.56%, and cross-modal fusion of MRI and PET scored an impressive 99.98%. While these near-perfect accuracies are presented as a major finding, the complete absence of a reproducible validation framework makes it impossible to independently assess their validity or the risk of methodological flaws like data leakage or overfitting. Consequently, these results must be viewed as preliminary and should be interpreted with extreme caution until a more detailed and transparent evaluation is published. *The progression of work from Kadri et al., from a single-modality model in 2021 to a four-modality framework in 2025, highlights a clear research trajectory towards embracing increasing multimodal complexity for enhanced diagnostic accuracy.*

*The following two studies used rs-fMRI and DTI for AD stages classification.* *First*, in 2023, Zuo et al. [177] introduced the Brain Structure-Function Fusing-Representation Learning (BSFL) model for AD classification using rs-fMRI and DTI data. BSFL’s architecture was a Serial Integration type enhanced with Cross-Attention, with these techniques detailed in Subsect. 4.4.2 and Subsect. 4.4.2. The model first employed a Knowledge-Aware Transformer (KAT) to extract structural features ($X_s$) from DTI by leveraging brain atlas information, while functional features ($X_f$) were derived from preprocessed rs-fMRI. For data integration, it used a Hybrid (Generative) fusion approach, a method described in Subsect. 6.1.4. The core of BSFL involved two Decomposed Variational Graph Autoencoders (VAEs) that disentangled $X_s$ and $X_f$ into uniform (shared) and unique (modality-specific) latent representations. A representation-fusing generator then adaptively merged these decomposed representations to create a unified brain network ($A_p$). The framework was trained adversarially using a dual discriminator system and a hybrid loss function incorporating a novel uniform-unique contrastive loss ($L_{UC}$) to enhance feature complementarity. The model was evaluated on the ADNI dataset using a 10-fold cross-validation protocol on a cohort of 322 subjects. While this provides a robust internal validation, the study’s evaluation was confined to this single cohort, with no testing on an independent external dataset to assess the model’s performance on data from different clinical environments. BSFL, when paired with a GCN classifier, achieved high performance, including up to 94.30% accuracy and 98.12% AUC for LMCI vs. NC classification, and 91.14% accuracy for LMCI vs. EMCI. These strong results underscore the potential of the decomposed-VAE fusion approach for MCI analysis; however, the lack of external validation means that the model’s generalizability remains unconfirmed. Further testing on diverse, multi-center cohorts is a critical next step to verify the clinical applicability of this advanced generative framework.

**Fig. 37:**
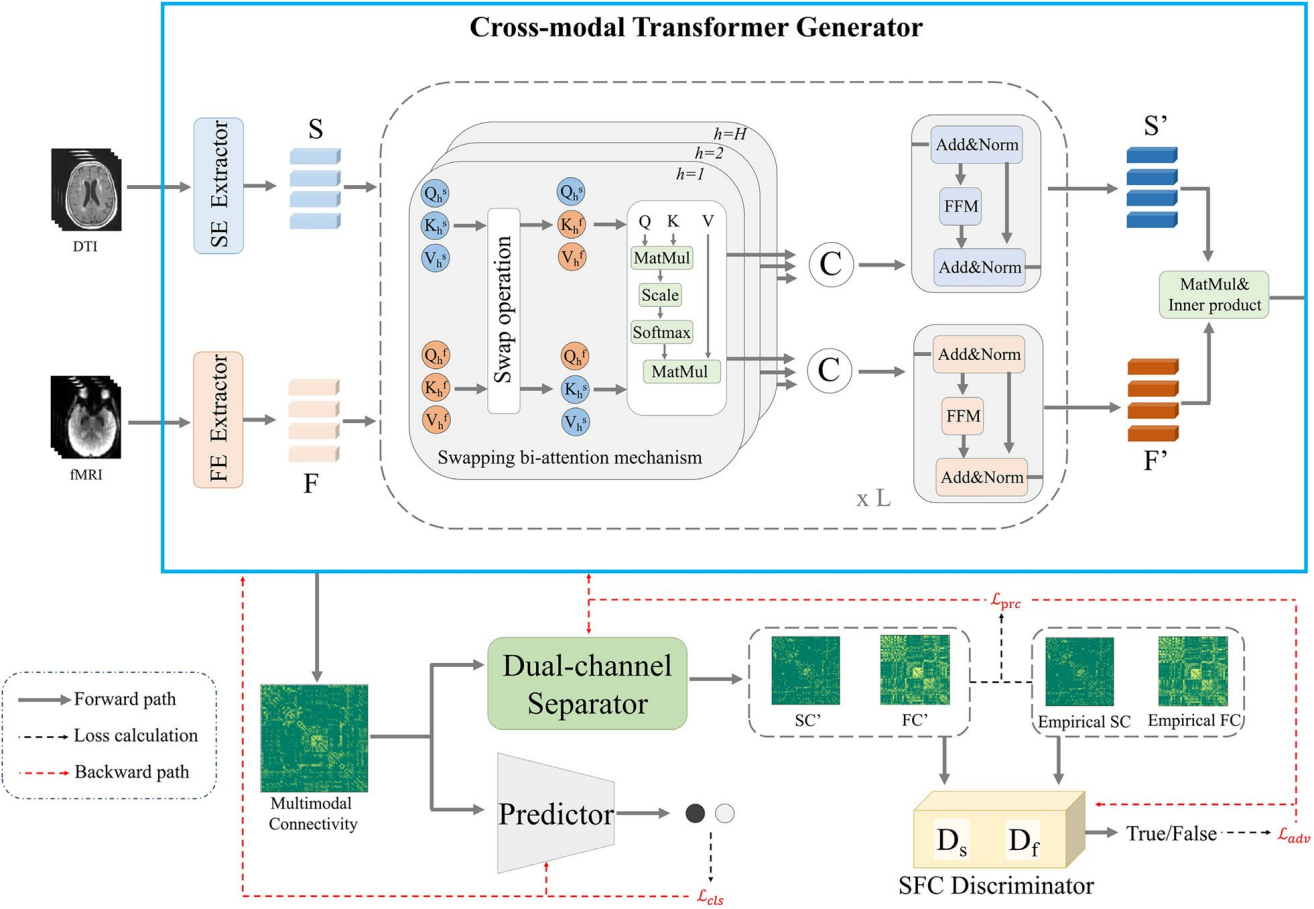
The architecture of the CT-GAN framework for fusing DTI and fMRI data. The model uses a generative adversarial approach where a Cross-Modal Transformer Generator, featuring a SBM, learns to create a unified multimodal connectivity matrix. This generated matrix, which represents the synergistic relationship between brain structure and function, is then used for AD classification. Figure adapted from [194]

*Second*, Zuo et al.  [194] introduced the Cross-Modal Transformer Generative Adversarial Network (CT-GAN) (Fig. 37). The framework’s architecture was a Serial Integration type with Cross-Attention, with these strategies described in Subsect. 4.4.2 and Subsect. 4.4.2. For data integration, it used a Hybrid (Generative) fusion strategy, as detailed in Subsect. 6.1.4. It featured a Cross-Modal Transformer Generator (*G*) that extracted ROI-based features from DTI and fMRI. Its core Swapping Bi-Attention Mechanism (SBM) then facilitated cross-modal fusion, where queries from one modality attended to the keys and values of the other:

60 $$\begin{aligned} S_{\text {Att}}^h&= \text {softmax}\left( \frac{Q_S^h (K_F^h)^T}{\sqrt{d_k}}\right) V_F^h \end{aligned}$$

61 $$\begin{aligned} F_{\text {Att}}^h&= \text {softmax}\left( \frac{Q_F^h (K_S^h)^T}{\sqrt{d_k}}\right) V_S^h \end{aligned}$$

This produced updated features ($S', F'$) used to compute a unified multimodal connectivity (MC) matrix ($MC = S'{F'}^T + F'{S'}^T$). A Dual-Channel Separator (DS) and a Structural-Functional Consistency (SFC) Discriminator were used to ensure the generated connectivity was realistic, while a Predictor module classified AD stages from the MC. The framework was trained with adversarial, classification, and pairwise connectivity reconstruction losses. The model was evaluated on the ADNI dataset (268 subjects) using a 5-fold cross-validation protocol. While this ensures a robust internal validation, the study’s evaluation was confined to this single cohort, with no testing on an independent external dataset to assess the model’s generalizability. CT-GAN achieved high accuracies for MCI progression, including 90.24% (NC vs. EMCI), 93.39% (EMCI vs. LMCI), and 95.19% (LMCI vs. AD). These strong results underscore the potential of the generative fusion approach for fine-grained MCI staging; however, the lack of external validation means that the model’s generalizability remains unconfirmed. Further testing on diverse, multi-center cohorts is a critical next step to verify the clinical applicability of this advanced generative framework. *These two studies from Zuo et al. showcased a sophisticated approach, moving beyond standard imaging to fuse DTI and rs-fMRI data within advanced Hybrid (Generative) frameworks, leveraging transformers to model complex brain structure-function relationships.*

#### Multimodal MCI to AD conversion prediction

Subsection 8.2.2 focused on a study that employed a CViT framework with multimodal inputs (sMRI and neurocognitive metadata) for the specific purpose of predicting MCI-to-AD conversion. This approach investigated the potential of combined data sources within a hybrid architecture to improve prognostic accuracy.

**Fig. 38:**
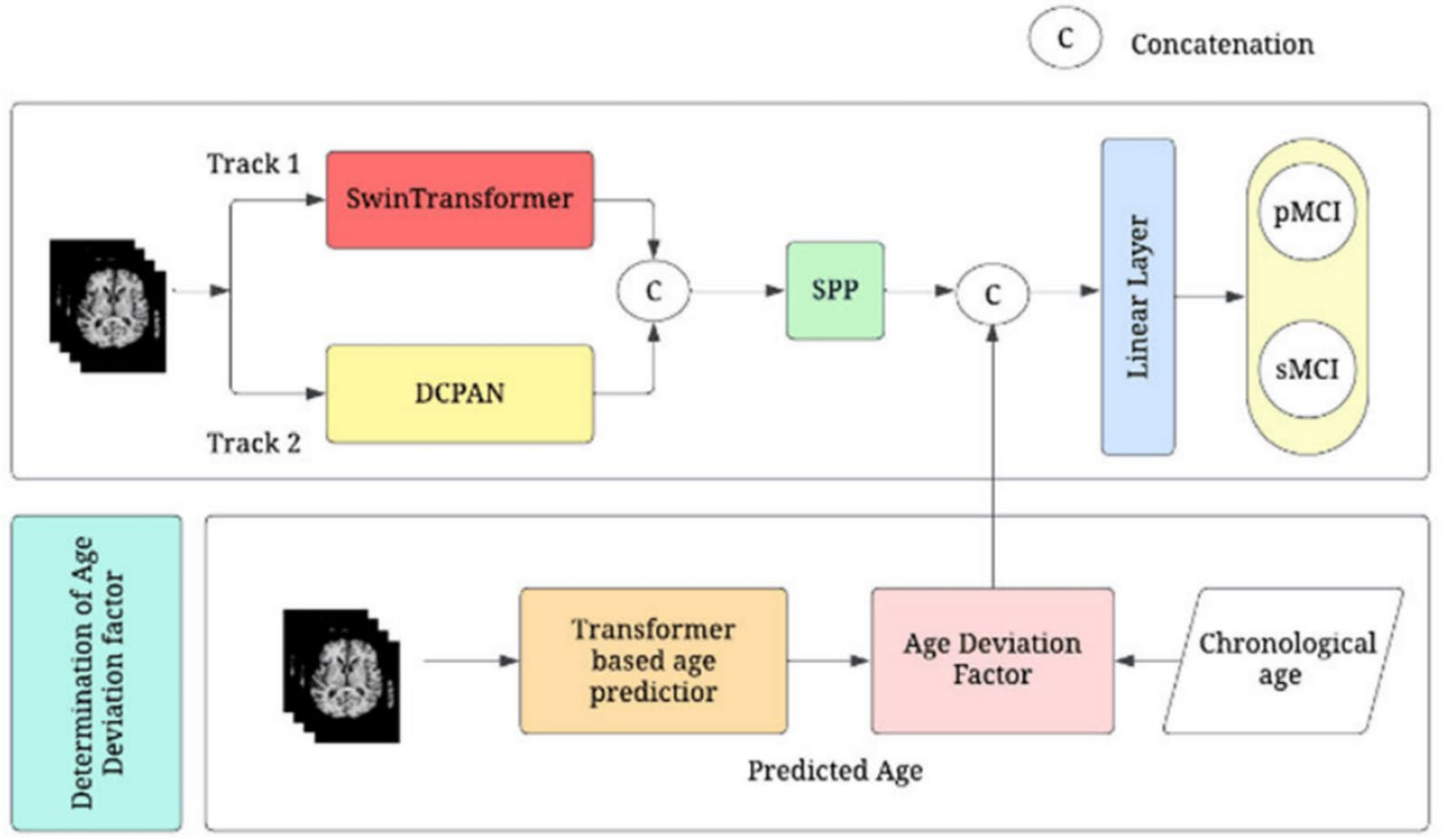
The parallel architecture of the multimodal framework for MCI conversion prediction. It features three concurrent processing streams: a Swin Transformer for sMRI analysis, a novel DCPAN, and an ADF. The framework fuses the outputs of these components to leverage complementary information for a more robust classification. Figure adapted from [183]

Illakiya et al.  [183] created a combined DL model to classify pMCI vs. sMCI using sMRI and age-related data (Fig. 38). The model implemented a Parallel Integration architecture, a design shown in Fig. 13a, with three distinct components processed concurrently. The first track employed a Swin Transformer on sMRI data to capture global context using its hierarchical structure with shifted window MHSA:

62 $$\begin{aligned} \hat{Z}^l&= \text {WMSA}(\text {LN}(Z^{l-1})) + Z^{l-1} \end{aligned}$$

63 $$\begin{aligned} Z^l&= \text {MLP}(\text {LN}(\hat{Z}^l)) + \hat{Z}^l \end{aligned}$$

The second track utilized a Dimension Centric Proximity Aware Attention Network (DCPAN), which performed ternary partitioning and then applied tri-branch neighborhood attention, where the attention weight $A_{ki}$ for an input was influenced by its *k*-nearest neighbors:

64 $$\begin{aligned} A_{ki} = \sum _{j=1}^{k} \left( Q_i \cdot K_{\phi _j(i)}^T + R(i, \phi _j(i)) \right) \end{aligned}$$

The third component was an Age Deviation Factor (ADF), calculated as the difference between chronological and predicted biological brain age. For data integration, the model used an Intermediate (Single-Level) fusion approach, as depicted in Fig. 14b. Features from the Swin Transformer and DCPAN tracks were fused, with a Spatial Pyramid Pooling (SPP) layer used for multi-scale representation, and the ADF was then incorporated as an additional feature. This combined representation was fed to a final classifier. The model was evaluated on the ADNI dataset. The validation was performed on a single 60:20:20 data split, and the study did not include an external validation on an independent cohort, which are important limitations to consider when interpreting the results. The complete model achieved 79.8% accuracy and a 78.4% F1-score, with ablation studies confirming the incremental benefit of each component. These results demonstrate the promise of the hybrid architecture for the challenging pMCI vs. sMCI task within the ADNI dataset, but the model’s generalizability to other clinical populations remains to be established through more extensive validation.

Luo and He  [143] introduced the Dual Attention Fusion Network (DAFN) for discriminating between progressive and stable MCI (pMCI vs. sMCI) using sMRI and neurocognitive metadata. The DAFN architecture was a Serial Integration type enhanced with Spatial Attention, with these techniques detailed in Fig. 13f and described in Subsect. 4.4.2. It first leveraged a 3D inflated ShuffleNet V2 to extract features from sMRI. For data integration, it used an Intermediate (Single-Level) fusion strategy, a method depicted in Fig. 14b and discussed in Subsect. 6.1.2. This was achieved via dual attention mechanisms: neurocognitive metadata (e.g., demographic data, cognitive scores, and brain volumetric measures) informed a spatial attention module applied to the image features. The metadata was also transformed into a class (CLS) token. This CLS token was then prepended to the sequence of image features and processed by a 3D ViT encoder, which used self-attention to integrate the image and metadata features. The model was evaluated on a combined ADNI1 and ADNI2 cohort using a 5-fold cross-validation protocol. While this provides robust internal validation, the study’s evaluation was confined to this single ADNI-based cohort, with no testing on an independent external dataset to assess the model’s generalizability. DAFN achieved an accuracy of 81.34%, with 85% sensitivity and 78% specificity for classifying pMCI vs. sMCI. These strong results highlight the potential of the dual-attention fusion mechanism for MCI prediction; however, the lack of external validation means that the model’s ability to generalize to different clinical populations remains an unverified but critical question for future work.

#### Multimodal AD classification and MCI conversion prediction

This subsection reviewed six studies that utilized CViT-based models with multimodal data to concurrently address both AD classification and MCI-to-AD conversion prediction. These investigations typically integrated sMRI with clinical, demographic, PET, or genetic data, aiming for a holistic diagnostic and prognostic assessment through sophisticated fusion strategies within the CViT architecture.

**Fig. 39:**
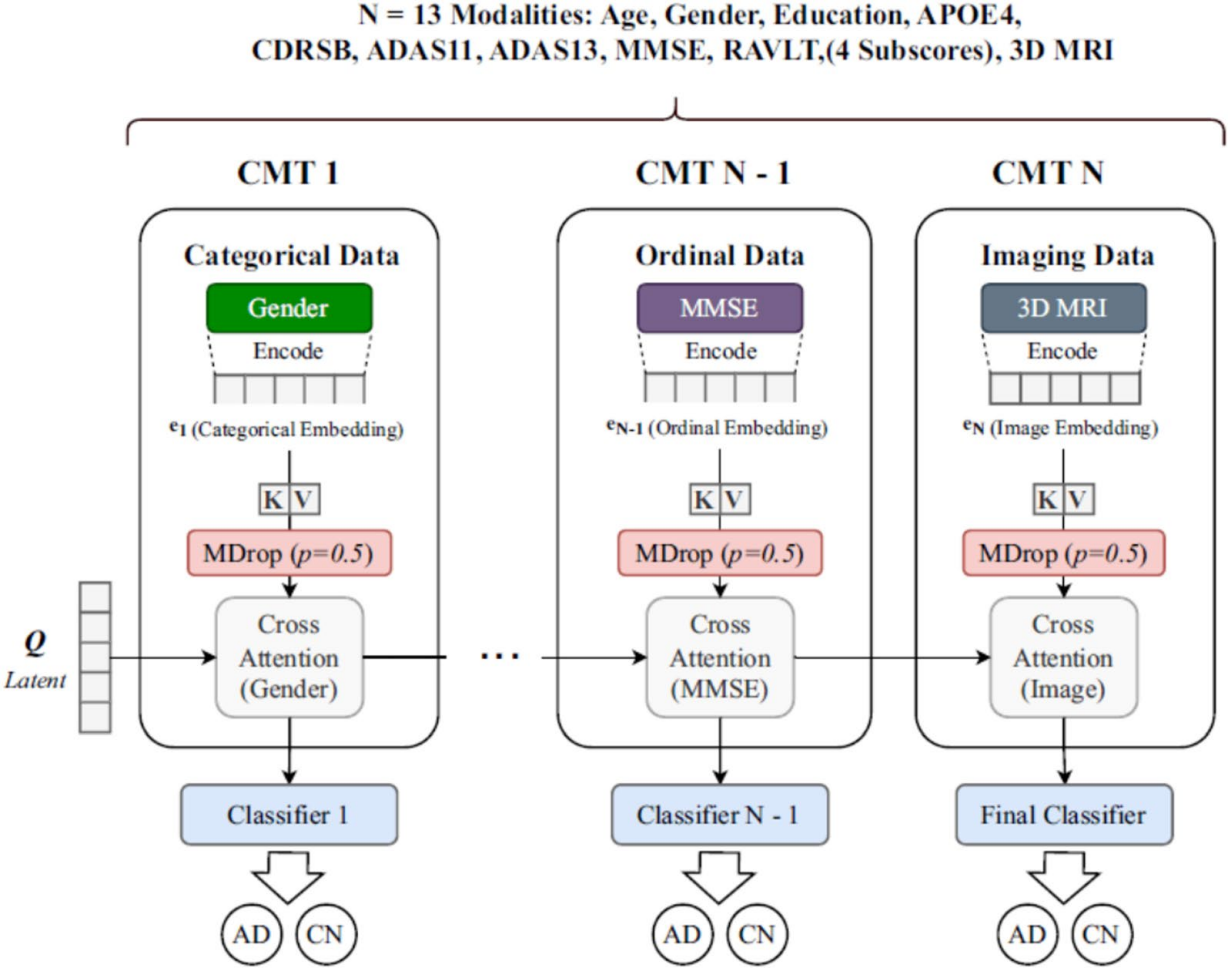
The 3MT network architecture, a cascaded transformer designed for robust multimodal fusion, even with missing data. The framework uses a series of CMTs to sequentially integrate information from sMRI and clinical data into a latent query vector. This iterative refinement process, combined with Modality Dropout during training, allows the model to make accurate predictions when one or more data sources are unavailable. Figure adapted from [50]

Liu et al.  [50] proposed the multimodal Mixing Transformer (3MT), a DL framework for AD classification and MCI conversion prediction using sMRI and clinical data, specifically designed to be robust to missing modalities (Fig. 39). This framework represented a Serial Integration architecture, a design described in Subsect. 4.4.2. For data integration, it used a Hybrid (Multi-Level System) fusion strategy, a method explained in Subsect. 6.1.4. The framework began with an optimizable latent query vector. Input modalities were first encoded, with sMRI processed by a hybrid CNN-Transformer encoder to yield Key (K) and Value (V) vector representations. Subsequently, the latent query was iteratively refined by passing through a sequence of Cascaded Modality Transformers (CMTs), where each CMT integrated a specific modality using both MHSA on the query and Multi-Head Cross-Attention (MHCA) between the query and the modality’s K/V representations. To enhance robustness, the model incorporated Modality Dropout (MDrop) during training to simulate missing data and used auxiliary classifiers after each CMT to provide intermediate supervision. The model was evaluated on the ADNI dataset for primary training and on the AIBL dataset for external validation. For the AD vs. CN task, 3MT achieved a high accuracy of 99.4% on ADNI and demonstrated strong generalizability on AIBL. For the more challenging task of MCI conversion prediction, however, validation was limited to a cross-dataset protocol within ADNI (training on ADNI-1 and testing on ADNI-2). While this approach tests for robustness across different scanner protocols, it does not represent a true external validation on a non-ADNI cohort. The model demonstrated competitive performance, with AUCs ranging from 83.97% to 89.89% in this cross-dataset MCI prediction task. The results strongly support the 3MT’s innovative capacity for handling missing data, but its generalizability for MCI prediction on truly independent, external cohorts remains an important area for future verification.

In another study, Gao et al.  [184] developed a hybrid DL model for AD diagnosis and MCI conversion prediction using sMRI and age. The architecture was a Serial Integration type, as described in Fig. 13b and Subsect. 4.4.2. The model featured a multi-scale attention convolution component to capture local brain variations, a pyramid non-local block to model long-range spatial correlations, and a novel aging transformer subnetwork. For data integration, it used an Intermediate (Single-Level) fusion approach (Fig. 14b) that incorporated Knowledge-Based principles, with these methods detailed in Subsect. 6.1.2 and Subsect. 6.1.4. This was achieved by the aging transformer, which embedded age information directly into image features and modeled dependencies between subjects of different ages to leverage age-related patterns. Commendably, the study employed a rigorous external validation protocol by training the model on the ADNI-1 dataset and evaluating it on the entirely separate ADNI-2 dataset. This cross-cohort approach directly tests the model’s ability to generalize across different scanner protocols and patient populations. The model achieved 90.5% accuracy and 93.9% AUC for AD vs. NC classification, and 73.6% accuracy and 73.7% AUC for pMCI vs. sMCI classification. The strong performance in this challenging cross-dataset setting underscores the robustness of the proposed aging transformer and multi-scale attention mechanisms. While validation on a non-ADNI cohort would be a valuable next step, these results provide significant evidence of the model’s generalizability beyond a single training distribution.

**Fig. 40:**
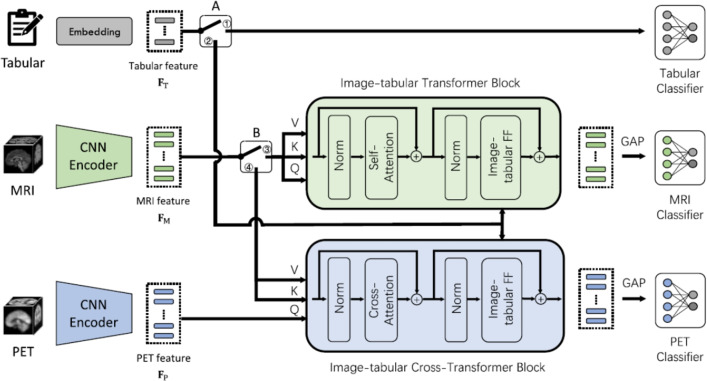
The architecture of the modality-flexible framework, which uses a sequential, three-branch pipeline to progressively integrate tabular, MRI, and PET data. This tripartite structure is designed to follow clinical data acquisition phases, allowing the model to make accurate predictions even when certain imaging modalities like PET are unavailable. Fusion is achieved via a novel image-tabular transformer. Figure adapted from [62]

*Addressing the phased nature of clinical data acquisition*, Zhang et al.  [62] proposed a modality-flexible framework (Fig. 40). The architecture implemented a Hierarchical Integration approach, a design detailed in Fig. 13c, by constructing a multi-stage pipeline across three sequentially trained branches. First, a Tabular Branch processed embedded tabular features ($F_T$) derived from categorical, continuous, or missing data:

65 $$\begin{aligned} F_{T,n} = f(x_n) = {\left\{ \begin{array}{ll} s_n^c, & \text {if } x_n \text { is categorical} \\ \text {MLP}(x_n), & \text {if } x_n \text { is continuous} \\ m_n, & \text {if } x_n \text { is missing} \end{array}\right. } \end{aligned}$$

For data integration, it used an Intermediate (Hierarchical) fusion strategy, a method depicted in Fig. 14c. The MRI and PET branches sequentially integrated imaging and tabular data using a novel adaptive feed-forward (FF) layer within an image-tabular transformer. This layer dynamically fused image features ($F_I$) and tabular features ($F_T$) using learned scaling ($\alpha$) and shift ($\beta$) coefficients:

66 $$\begin{aligned} \alpha&= \text {Sigmoid}(\text {FC}_{\alpha }(F_I, F_T)) \end{aligned}$$

67 $$\begin{aligned} \beta&= \text {FC}_{\beta }(F_I, F_T) \end{aligned}$$

The final fused features ($F_{IT}$) were then computed as:

68 $$\begin{aligned} F_{IT,i} = \alpha _i F_{I,i} + (1 - \alpha _i)F_{T,i} + \beta _i \end{aligned}$$

The PET branch first fused CNN-extracted PET ($F_P$) and MRI ($F_M$) features via a cross-transformer before integrating this fused representation with $F_T$ using the same adaptive FF layer. The framework’s efficacy was extensively evaluated on a large, multi-cohort dataset ($n=6495$; ADNI, OASIS-3, NACC, C-PAS). This rigorous validation on a large-scale, heterogeneous dataset represents a significant methodological strength and a best-practice example for the field. Internal validation using all available modalities yielded overall accuracies of 77.9% for cognitive assessment (COG), 76.1% for MCI conversion prediction (MCIC), and 89.9% for dementia assessment (ADD). These performance metrics, while more modest than those often reported in single-cohort studies, likely represent a more realistic and sober benchmark for real-world clinical performance. The study’s results empirically underscore the challenge of domain shift, as performance on the external C-PAS cohort was notably lower, highlighting the critical importance of multi-center validation for developing truly generalizable diagnostic models.

**Fig. 41:**
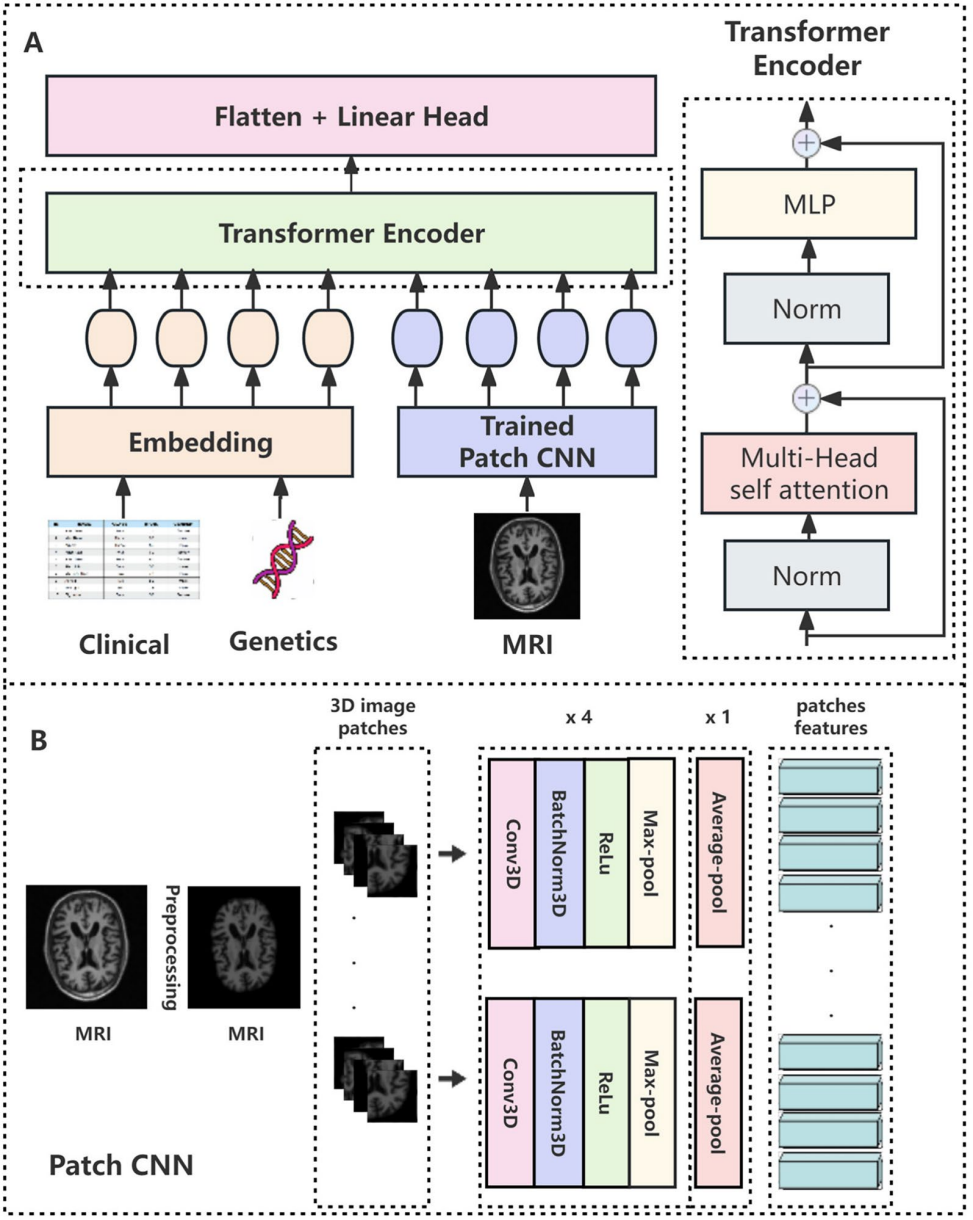
The architecture of the AD-Transformer, a unified model for fusing imaging, clinical, and genetic data. It operates by converting all input modalities into a common token-based representation: a Patch-CNN tokenizes the sMRI scan, while other encoders handle the tabular data. These diverse tokens are then combined into a single sequence and fed to a Transformer Encoder, which learns a holistic representation for AD classification. Figure adapted from [49]

*For both AD classification and MCI prediction using sMRI, clinical, and genetic data*, Yu et al. (2024) [49] introduced the AD-Transformer, a unified framework (Fig. 41A). This approach implemented a Serial Integration architecture, as described in Fig. 13b and Subsect. 4.4.2. The framework first tokenized inputs: a Patch-CNN module (Fig. 41B) processed 3D sMRI into image tokens capturing local spatial features, while a linear projection layer converted preprocessed clinical and genetic information into non-image tokens. For data integration, it used an Intermediate (Single-Level) fusion approach, a method illustrated in Fig. 14b and explained in Subsection ??. The diverse tokens from imaging, clinical, and genetic data were concatenated with positional encodings and fed into a central Transformer Encoder block. This block employed MHSA to learn holistic multimodal representations by capturing complex interdependencies both within and across modalities. The aggregated output was then passed to an MLP with Softmax for the final prediction. The model was evaluated on a large cohort from the ADNI dataset (1651 subjects). While the use of 5-fold cross-validation provides robust internal validation, the study’s findings are confined to this single, relatively homogeneous cohort, as no external validation was performed. The authors themselves note this as a limitation, citing the difficulty in finding external datasets with comparable multimodal data. AD-Transformer achieved average AUCs of 0.993 for AD vs. CN classification and 0.845 for MCI conversion prediction. These high performance metrics underscore the power of the unified fusion architecture within the ADNI dataset, but the model’s generalizability to more diverse clinical populations and data sources remains an open and critical question for future research.

*The following study applied both AD classification and MCI prediction using sMRI and PET.* In 2023, Gao et al. [172] proposed a two-part DL system for AD classification and MCI prediction using multimodal neuroimaging (T1w-sMRI, PET, T2w-sMRI), specifically designed to handle missing scans. The architecture represented a Serial Integration approach, as described in Fig. 13b and Subsect. 4.4.2. For data integration, it used a Hybrid (Generative) fusion strategy, as detailed in Subsect. 6.1.4. The initial stage utilized a Multi-Level Guided Generative Adversarial Network (MLG-GAN) to generate absent imaging data. This MLG-GAN leveraged guidance across voxel, feature (using an auto-regression component), and task levels to produce diagnostically meaningful synthetic images. The second stage employed a Multimodal Transformer (Mul-T) for disease classification using the now complete imaging sets. Mul-T featured CNN pathways for extracting both localized and global image characteristics, followed by cross-modal transformer modules that modeled and integrated information across the different imaging types. The framework was trained on ADNI-1 and tested on the independent ADNI-2 dataset. This cross-cohort evaluation is a notable methodological strength, setting a higher standard for validation than single-cohort studies. The model achieved 94.4% accuracy (97.6% AUC) for AD vs. CN and 77.8% accuracy (82.8% AUC) for pMCI vs. sMCI classification. These promising results should, however, be contextualized by the fact that both cohorts originate from the same overarching ADNI protocol, which may not fully capture the domain shift of truly independent clinical sites. Additionally, the reliance on a generative model trained on a limited dataset introduces complexities regarding the biological plausibility of the synthetic data and its influence on the final classification performance.

### Analysis of CViT applications

A comprehensive synthesis of the 46 reviewed studies on CViT applications in AD research, as outlined in Fig. 15 and detailed in Table 5, reveals a field characterized by rapid innovation and a clear trajectory towards architectural and multimodal sophistication. This analysis demonstrates an evident evolution from foundational hybrid models to highly specialized frameworks designed to tackle the nuanced challenges of AD diagnosis. The discussion deconstructs the common operational workflows (Subsect. 8.3.1), trends in data utilization (Subsect. 8.3.2), patterns in dataset dependency and validation rigor (Subsect. 8.3.3), the distribution of diagnostic objectives (Subsect. 8.3.4), and dominant architectural and fusion strategies (Subsect. 8.3.5).

#### General operational pipeline in CViT applications

An examination of the contemporary literature employing CViTs for AD and MCI assessment reveals a structured yet highly adaptable operational pipeline. The term CViT, as delineated in Subsect. 4.4.2, encapsulates a diverse spectrum of hybrid architectures that synergistically combine the local feature-learning prowess of CNNs with the global context-modeling capabilities of transformers [208]. This inherent architectural flexibility has enabled researchers to tailor solutions for a wide range of data types and complexities. This is particularly evident in studies integrating challenging modalities like fMRI and DTI, which have moved beyond rudimentary CViT designs towards highly sophisticated frameworks, such as the BSFL model by Zuo et al. [177] and the CT-GAN [194] (Fig. 37).

The typical workflow begins with rigorous, modality-specific preprocessing. For sMRI, this involves a standard cascade of spatial normalization, skull stripping, and registration (exemplified in Fig. 5). For other modalities, distinct pipelines are necessary; for instance, fMRI data requires motion correction and temporal filtering to construct dynamic functional connectivity networks (dFCNs) as seen in LCGNet by Zhou et al. [140] (Fig. 30a), while DTI processing often involves FA map generation as seen in the work of Zuo et al. [177].

The critical divergence from pure ViT models occurs at the feature extraction and integration stages, which are defined by the specific Architectural Integration Pattern employed:

- *Serial integration:* This is the most prevalent pattern, where a CNN acts as a dedicated feature extraction front-end. Examples include using a VGGNet-16 to tokenize 2D slices for a subsequent Swin Transformer [81] (Fig. 26b) or employing a 3D CNN encoder to generate feature tokens that are then fused with clinical data in a transformer block [49, 102].
- *Parallel integration:* In this design, CNN and Transformer branches operate concurrently to extract features, which are then fused. This approach is exemplified by LCGNet [140], which processes rs-fMRI with parallel CNN and Transformer streams, and by Sait et al., who combined different transformer backbones [141, 187].
- *Hierarchical integration:* This represents the most synergistic approach, where convolutional and attention mechanisms are deeply interleaved within the network’s core blocks. This is demonstrated in the advanced, computationally efficient models by Khatri et al. [30, 145] (Fig. 27), which alternate specialized convolution and attention modules, as well as in frameworks like MDL-Net [193] and MMTFN [147].

CViTs demonstrate exceptional strength in multimodal data integration. The integration of modality-specific features is realized through a variety of fusion strategies:

- *Intermediate (Feature-level) fusion:* This is the dominant strategy. After initial feature extraction, feature sets are combined. This is often achieved with sophisticated, attention-based methods, including **Attention-Based** cross-modal fusion (as in MCAD [155], Fig. 36, and CsAGP [156]) and **Hierarchical** fusion that combines features at multiple network depths (as in MMTFN [147], Fig. 34, and the framework by Zhang et al. [62]).
- *Input-level (Signal-level) fusion:* Less common but notably explored, this involves merging raw imaging data before it enters the primary feature extractor. The series of studies by Odusami et al. [148–151] are prime examples, utilizing techniques like DWT and SPCNN for pixel-level fusion, as conceptually shown in their framework (Fig. 33).
- *Hybrid and knowledge-based fusion:* The most advanced frameworks often employ hybrid strategies. This includes generative models that learn a fused representation, as seen in the DTI-fMRI fusion work by Zuo et al. [177, 194] and Gao et al. [172], or complex systemic models that iteratively integrate modalities [50] (Fig. 39) or incorporate multi-task learning [154] (Fig. 35).

This general CViT workflow—encompassing data acquisition, modality-specific preprocessing, a choice of architectural integration pattern, and an advanced multimodal fusion strategy—underpins the innovative applications in AD research. The high degree of variation in these choices reflects a dynamic field striving to optimize the balance between local detail, global context, and computational efficiency.

#### Modality utilization: sMRI prominent in single-modality, strong shift to multimodal frameworks

The reviewed literature applying the aforementioned CViT operational pipelines demonstrated distinct preferences in data utilization. Of the 46 CViT-based studies, 20 focused on single-modality data. Within this group, sMRI was the preponderant choice, utilized in 16 studies (approximately 80% of single-modality CViT applications), including works by Kadri et al. [153] and Huang et al. [209]. In contrast, dedicated CViT research on single-modality FDG-PET (e.g., Rehman et al. [165]) or rs-fMRI (e.g., Zuo et al. [212]) was less frequent. This distribution, visualized in Fig. 25, suggested an area for future exploration with other imaging types.

A significant finding was that the majority of CViT research (26 out of 46 studies, ~57%) employed multimodal approaches, indicating a stronger tendency towards integrating diverse data sources compared to standalone ViT studies. The observed combinations were diverse, ranging from the common pairing of sMRI with clinical data (e.g., Dai et al. [102]; Liu et al. [50]; Gao et al. [184]) to the integration of sMRI with PET scans, as seen in the work of Kadri et al. [178] and Tang et al. [156]. More complex frameworks fused sMRI with clinical and genetic data (e.g., Yu et al. [49]; Zhang et al. [62]), while the most comprehensive approaches sought to integrate three or more modalities, such as the DTI and rs-fMRI fusion by Zuo et al. [177, 194] or the combination of MRI, PET, DTI, and sfMRI by Kadri et al. [188]. This preference underscored the perceived suitability of CViTs for managing the architectural complexities inherent in fusing heterogeneous information.

#### Dataset dependency: ADNI remains central, with increased efforts in generalization testing

The application of these CViT pipelines predominantly relied on the ADNI dataset, which was featured in approximately 33 of the 46 studies (~72%), spanning both single-modality [30, 31] and multimodal investigations [50, 102]. However, a positive trend towards broader validation was evident in the CViT literature, with a significant number of studies incorporating external or multiple datasets to test for generalization. For instance, several researchers performed external validation by making dual use of the ADNI and AIBL datasets, as seen in the work of Huang et al. [209] and Xin et al. [31]. Similarly, others employed both ADNI and OASIS to test their models’ robustness, as demonstrated by Hu et al. [81] and Kadri et al. [153]. Multimodal investigations, in particular, showed a commitment to more rigorous validation across multiple cohorts. This was exemplified by Gao et al. [172], who used ADNI-1, ADNI-2, and OASIS-3, and by the extensive multi-cohort analysis from Zhang et al. [62] across ADNI, OASIS-3, NACC, and C-PAS. Furthermore, the series of fusion studies by Odusami et al. [149–151] consistently validated their methods on the ADNI, OASIS, and AANLIB datasets. This increased effort in generalization testing was more pronounced compared to the trends observed in standalone ViT studies.

#### Diagnostic focus and technical sophistication

The primary diagnostic objective for the reviewed CViT applications was AD classification (e.g., AD vs. CN, multiclass staging), addressed by 18 single-modality [31, 153] and 19 multimodal studies [49, 102]. Prediction of MCI progression was the sole focus in fewer instances, such as Hu et al. [131] (single-modality sMRI) and Luo et al. [143] (multimodal sMRI + neurocognitive data). A notable characteristic of the CViT literature was the relatively higher number of studies (approximately 13% of all CViT studies) tackling both AD classification and MCI conversion prediction within a unified framework. This included one single-modality sMRI study by Zhao et al. [32] and six multimodal investigations [49, 50, 62, 172, 184].

#### Architectural strategies and fusion techniques

The reviewed studies consistently operationalized the hybrid CViT principles detailed in the general pipeline. The **Serial Integration** pattern, involving a CNN front-end, was frequently observed in models like Swin Transformer-based frameworks [31, 81, 131, 209] and CrossViT [153].

The 26 multimodal CViT investigations, in particular, showcased the application of diverse and sophisticated fusion methods. **Intermediate (Feature-level) Fusion** was the most widespread strategy, often enhanced by attention mechanisms as demonstrated in DE-JANet [102] and MCAD [155]. **Input-Level (Signal-level) Fusion** was notably implemented by Odusami et al. through various pixel-level techniques [148–151]. **Knowledge-Based Fusion** principles were evident when non-imaging data, such as age or genetics, modulated the fusion process, as seen in the work of Gao et al. [184] and Yu et al. [49]. Finally, complex **Hybrid Fusion** strategies, such as the VAE-based generative frameworks by Zuo et al. [177], confirmed the field’s push towards more powerful and holistic integration schemes.

In summary, the analysis of the reviewed literature reveals that CViTs have become powerful and versatile architectures in AD research, excelling in multimodal data integration and complex diagnostic tasks. The field demonstrates a clear preference for CViT models in multimodal settings, a growing trend towards more rigorous validation across diverse datasets, and an increasing application to combined diagnostic and prognostic tasks. The synergistic combination of CNN-based local feature extraction and Transformer-based global attention modeling has proven highly suitable for the multifaceted nature of AD. A broader discussion of the overall limitations and future directions for the field is presented in Section 12.

**Table 4:** Summary of studies using ViTs for AD classification and MCI conversion prediction

Author	Year	Diagnostic task	Modality	Modality type	Multimodal data fusion type	Dataset	Technique	Classification	Internal validation	External validation	Performance				
											Accuracy	Sensitivity	Specificity	Precision	AUC
Mora-Rubio et al. [161]	2023	AD Classification	Single	sMRI	–	Combined ADNI & OASIS	ViT	AD vs. CN (ViT)	60%–20%–20% (Train-Validation-Test) (T-V-T)	–	89.02%	89.02%	74.01%	–	–
Carcagnĺ et al. [158]	2023	AD Classification	Single	sMRI	–	ADNI2, OASIS1	CNN (ResNet, DenseNet, EfficientNet) & Transformer-based (MAE, DeiT)	AD vs. NC (DeiT)	5-fold CV	–	77.0% (ADNI2) 74.4% (OASIS1)	–	–	–	79.00% (ADNI2) 74.00% (OASIS1)
Alp et al. [134]	2024	AD Classification	Single	sMRI	–	ADNI1: Complete 1Yr 1.5T ; ADNI1: Complete 3Yr 3T	(ViT + Time Series Transformer)	AD vs. CN	60%–20%–20% (T-V-T)	–	95.169% (ADNI1: 1Yr) 99.048% (ADNI1: 3Yr)	95.50% (ADNI1: 1Yr) 99.50% (ADNI1: 3Yr)	–	–	–
Hoang et al. [207]	2023	MCI Prediction	Single	sMRI	–	ADNI	ViT	MCIC vs. MCINC	90%–10% (T-T)	–	83.27%	85.07%	81.48%	–	–
Joy et al. [195]	2025	AD Classification	Single	sMRI	–	ADNI	ViTAD	CN vs. EMCI vs. LMCI vs. MCI vs. AD	85%–15% (T-T)	–	99.98%	100%	–	100%	–
Mahim et al. [197]	2024	AD Classification	Single	sMRI	–	Kaggle, ADNI	ViT-GRU	ND vs. VMD vs. MD vs. MoD	10-fold CV	–	99.53% (Dataset 1)	99.53% (Dataset 1)	99.76% (Dataset 1)	99.54% (Dataset 1)	–
								AD vs. MCI vs. CN			99.26% (ADNI)	99.26% (ADNI)	99.45% (ADNI)	99.27% (ADNI)	–
Almufareh et al. [196]	2023	AD Classification	Single	sMRI	–	OASIS1	ViT	ND vs. VMD vs. MD vs. MoD	80%–20% (T-V)	–	99.06%	99.14%	–	99.06%	–
Shah et al. [198]	2024	AD Classification	Single	sMRI	–	Kaggle, ADNI (Kaggle)	BiViT	ND vs. VMD vs. MD vs. MoD	(without data augmentation)	–	96.0%	98.0%	–	88.0%	99.0%
								CN vs. EMCI vs. LMCI vs. MCI vs. AD	(with data augmentation)		45.0%	69.0%	–	33.0%	73.0%
Alshayeji [199]	2024	AD Classification	Single	sMRI	–	Kaggle	Pre-trained (ViT)	ND vs. VMD vs. MD vs. MoD	85%–15% (T-T)	–	99.83%	99.69%	99.88%	99.54%	–
Hosny et al. [159]	2024	AD Classification	Single	sMRI	–	Kaggle	EfficientViT	ND vs. VMD vs. MD vs. MoD	5-fold CV	–	99.24%	99.38%	–	99.02%	–
Pramanik et al. [200]	2024	AD Classification	Single	sMRI	–	Kaggle	FGI-CogViT	ND vs. VMD vs. MD vs. MoD	80%–20% (T-T)	–	98.83%	99.48%	–	99.52%	–
Kurniasari et al. [201]	2025	AD Classification	Single	sMRI	–	Kaggle	ViT	ND vs. VMD vs. MD vs. MoD	81%–9%–10% (T-V-T)	–	98.19%	96.34%	98.80%	–	–
Lu et al. [202]	2025	AD Classification	Single	sMRI	–	OASIS1	RanCom-ViT	ND vs. VMD vs. MD vs. MoD	80%–20% (T-T)	–	99.54%	98.82%	99.63%	99.55%	–
Shin et al. [162]	2023	AD Classification	Single	18F-Florbetaben PET	–	Dong-A University Hospital cohort	ViT	HC vs. (AD + MCI) (Original Data)	60%–20%–20% (T-V-T)	–	80.00%	60.00%	–	75.00%	–
Khatri and Kwon [166]	2023	MCI Prediction	Single	18F-FDG PET	–	ADNI1/2/GO	ViT-DINO	MCI-c vs MCI-s	5-fold CV	–	92.31%	90.21%	95.50%	93.10%	96.00%
Zhang et al. [203]	2022	AD Classification	Single	rs-fMRI	–	ADNI	KD-Transformer	EMCI vs. HC	80%–20% (T-V)	–	80.0%	–	–	–	–
Sarraf et al. [204]	2023	AD Classification	Single	rs-fMRI, sMRI	–	ADNI	OViTAD	AD vs. HC vs. MCI	80%–10%–10% (T-V-T)	–	87.00% (sMRI) 97.00% (fMRI)	87.00% (sMRI) 97.00% (fMRI)	–	81.00% (sMRI) 97.00% (fMRI)	–
He et al. [205]	2024	AD Classification	Single	rs-fMRI	–	ADNI-1/2/3	STGTN	AD vs. NC	5-fold CV with AE augmentation	–	92.58% (ADNI-1/2) 92.30% (ADNI3)	96.88% (ADNI-1/2) 95.81% (ADNI3)	94.03% (ADNI-1/2) 94.00% (ADNI3)	–	98.67% (ADNI-1/2) 98.45% (ADNI3)
Wang [206]	2025	AD Classification	Single	rs-fMRI (BOLD)	–	ADNI2	VTFF	HC vs. EMCI vs. LMCI vs. AD	80%–20% (T-T)	–	84.2%	81.0%	94.0%	85.0%	88.0%
Saoud and AlMarzouqi [163]	2024	AD Classification & MCI Prediction	Single	sMRI	–	ADNI	3D-ViTs with DBN	AD vs. CN	80%–20% (T-V)	–	90.00%	–	–	–	91.00%
								pMCI vs. sMCI			94.00%	–	–	–	95.00%
Castro-Silva et al. [191]	2024	AD Classification	Multi	sMRI, Clinical data	Intermediate (Single-Level)	ADNI, AIBL, OASIS	MIMD-3DVT	AD vs. CN	7-fold CV	–	97.14%	–	–	–	98.4%
Zou et al. [109]	2024	AD Classification & MCI Prediction	Multi	fMRI, SNP (genetic data)	Hybrid (Graph-based)	ADNI	BIGFormer	HC vs. AD	10-fold CV	–	91.87%	89.74%	92.86%	–	94.14%
								sMCI vs. pMCI			85.71%	84.62%	86.67%	–	88.21%

**Table 5:** Summary of studies using CViTs for AD classification and MCI conversion prediction

Author	Year	Diagnostic task	Modality	Modality type	Multimodal data fusion type	Dataset	Technique	Architectural integration type	Classification	Internal validation	External validation	Performance				
												Accuracy	Sensitivity	Specificity	Precision	AUC
Kadri et al. [153]	2021	AD Classification	Single	sMRI	–	ADNI, OASIS	(CrossViT + (WRSE-Net) + ProGAN)	Serial & (Channel Attention)	AD vs. MCI vs. CN	–	–	99.0%	–	–	–	–
Zhu et al. [160]	2022	AD Classification	Single	sMRI	–	ADNI	BraInf	Hierarchical	AD vs. NC	10-fold CV	–	97.97%	97.74%	98.17%	98.16%	–
Huang et al. [209]	2023	AD Classification	Single	sMRI	–	ADNI, AIBL	RST	Serial	AD vs. NC	70%–10%–20% (T-V-T)	AIBL	99.59% (ADNI) 94.01% (AIBL)	99.59% (ADNI)	99.58% (ADNI)	99.83% (ADNI)	–
Hu et al. [81]	2023	AD Classification	Single	sMRI	–	ADNI, OASIS	Conv-Swinformer	Serial	AD vs. CN	70%–15%–15% (T-V-T)	OASIS	93.56% (ADNI) 92.31% (OASIS)	93.81% (ADNI)	93.31% (ADNI)	–	97.49% (ADNI) 94.17% (OASIS)
Hu et al. [131]	2023	MCI Prediction	Single	sMRI	Intermediate (Temporal)	ADNI	VGG-TSwinformer	Serial	pMCI vs. sMCI	65%–20%–15% (T-V-T)	–	77.20%	79.97%	71.59%	–	81.53%
Xin et al. [31]	2023	AD Classification	Single	sMRI	–	ADNI, AIBL	ECSnet	Serial	AD vs. CN	5-fold CV	AIBL	93.9% (ADNI) 93.9% (AIBL)	92.5% (ADNI) 91.1% (AIBL)	94.7% (ADNI) 94.4% (AIBL)	–	96.4% (ADNI) 96.3% (AIBL)
Zhao et al. [32]	2023	AD Classification & MCI Prediction	Single	sMRI	–	ADNI, AIBL	IDA-Net	Hierarchical	AD vs. NC	60%–20%–20% (T-V-T)	AIBL	92.7% (ADNI) 90.9% (AIBL)	91.9% (ADNI) 90.3% (AIBL)	94.6% (ADNI) 93.5% (AIBL)	–	97.2% (ADNI) 96.1% (AIBL)
									pMCI vs. sMCI			83.5% (ADNI) 81.2% (AIBL)	80.2% (ADNI) 79.00% (AIBL)	85.5% (ADNI) 83.5% (AIBL)	–	87.7% (ADNI) 85.4.9% (AIBL)
Khatri et al. [145]	2024	AD Classification	Single	sMRI	–	ADNI	(MBConv + PConv + IRFFN)	Hierarchical	AD vs. HC	10-fold CV	–	97.29%	95.96%	96.15%	97.14%	
Khatri and Kwon [30]	2024	AD Classification	Single	sMRI	–	ADNI	Optimized CViT	Hierarchical	AD vs. HC	90%–10% (T-T)	–	95.37%	91.09%	100%	–	–
Li et al. [144]	2024	AD Classification	Single	sMRI	–	ADNI	LD-MILCT	Serial	AD vs CN (TD)	10-fold CV	–	91.0%	90.3%	95.7%	–	–
Zhang et al. [157]	2024	AD Classification	Single	sMRI	–	ADNI	RepBoTNet-CESA	Serial & MHSA	AD vs. NC	5-fold CV	–	96.58%	96.23%	–	97.26%	–
Huang and Qiu [210]	2024	AD Classification	Single	sMRI	–	ADNI, OASIS3	MC-ViT	Serial	AD vs. NC	ADNI: 37.5%–12.5%–50% (T-V-T)	OASIS3	–	90.07% (ADNI) 77.80% (OASIS3)	90.22% (ADNI) 82.76% (OASIS3)	–	95.57% (ADNI) 86.32% (OASIS3)
Poonia and Al-Alshaikh [152]	2024	AD Classification	Single	sMRI	–	ADNI	(InceptionV3, VGG19, ResNet50, DenseNet121) + ViT	Serial & (Late fusion)	Early vs. Mild vs. High vs. Normal	10-fold CV; 70%–30% (T-T)	–	96% (InceptionV3+ ViT)	90% (InceptionV3+ ViT)	–	94% (InceptionV3+ ViT)	–
Sait [141]	2024	AD Classification	Single	sMRI	–	OASIS, Kaggle	LeViT + EfficientNet B7	Parallel	ND vs. VMD vs. MD vs. MoD	OASIS: 70%–15%–15% (T-V-T)	Kaggle	99.8%	99.4%	99.8%	–	0.99
Sait and Nagaraj [187]	2024	AD Classification	Single	sMRI	–	Kaggle, OASIS (Kaggle)	(CCT-Linformer + TT-Performer)	Parallel	ND vs. VMD vs. MD vs. MoD	Kaggle: 70%–15%–15% (T-V-T)	OASIS (20% T)	99.2% (Kaggle) 98.8% (OASIS)	98.9% (Kaggle) 98.1% (OASIS)	98.6% (Kaggle) 97.5% (OASIS)	98.9% (Kaggle) 97.9% (OASIS)	–
Menon and Gunasundari [211]	2024	AD Classification	Single	sMRI	–	Kaggle	(DenseNet-121 + ViT)	Parallel	ND vs. VMD vs. MD vs. MoD	–	–	93.0% (ViT) 99.0% (Fusion)	93.0% (ViT) 99.0% (Fusion)	–	93.0% (ViT) 99.0% (Fusion)	–
Rehman et al. [165]	2024	AD Classification	Single	18F-FDG PET	–	ADNI	ResGLPyramid	Hierarchical	AD vs. NC	10-fold CV	–	97.00%	96.10%	97.50%	–	96.90%
Zuo et al. [212]	2023	AD Classification	Single	fMRI	–	ADNI	DAGAE + GCN classifier	Serial	LMCI vs. NC (GCN)	5-fold CV	–	85.33%	84.00%	86.67%	–	86.42%
Zhou et al. [140]	2024	AD Classification	Single	rs-fMRI	–	ADNI	LCGNet	Parallel	AD vs. NC	5-fold CV		95.3%	93.3%	96.7%	–	96.5%
Zhang et al. [146]	2024	AD Classification	Single	rs-fMRI	–	ADNI2, ADNI3, OASIS, HUASHAN-MCI	HFBN (ST-GCFE (GCN) +HNFM (Transformer))	Hierarchical	MCI vs. NC	5-fold CV	–	83.7% (ADNI2) 89.9% (OASIS)	91.3% (ADNI2) 76.7% (OASIS)	70.1% (ADNI2) 96.7% (OASIS)	80.7% (ADNI2) 81.2% (OASIS)	
Dai et al. [102]	2023	AD Classification	Multi	sMRI, Clinical Data	Intermediate (Single-Level)	ADNI1, ADNI2	DE-JANet	Serial	AD vs. NC	ADNI1	ADNI2	97.22% (ADNI2)	98.08% (ADNI2)	96.43% (ADNI2)	96.23% (ADNI2)	–
Chen et al. [142]	2025	AD Classification	Multi	sMRI, Clinical Data	Intermediate (Single-Level)	ADNI	(MMDF + ViT + CNN)	Parallel	AD vs. CN vs. MCI	70%–20%–10% (T-V-T)	–	97.65%	96.40%	–	96.98%	–
Illakiya et al. [183]	2023	MCI Prediction	Multi	sMRI, Clinical data	Intermediate (Single-Level)	ADNI	(Swin Transformer + DCPAN + ADF)	Parallel	pMCI vs sMCI	4-fold CV	–	70.21% (Swin Transformer) 79.8% (Fusion)	66.86% (Swin Transformer) 80.23% (Fusion)	–	67.64% (Swin Transformer) 76.66% (Fusion)	–
Luo and He [143]	2024	MCI Prediction	Multi	sMRI, Neurocognitive Metadata	Intermediate (Single-Level)	ADNI1, ADNI2	DAFN	Serial & (Spatial Attention)	pMCI vs. sMCI	5-fold CV	–	81.34%	85.0%	78.0%	–	87.4%
Liu et al. [50]	2023	AD Classification & MCI Prediction	Multi	sMRI, Clinical Data	Hybrid (Multi-Level System)	ADNI, AIBL	3MT	Serial	AD vs. CN	5-fold CV	AIBL	99.4% (ADNI) 96.3% (AIBL)	100.0% (ADNI) 97.5% (AIBL)	98.9% (ADNI) 90.3% (AIBL)	–	99.7% (ADNI) 98.4% (AIBL)
									MCI to AD conversion	5-fold CV (ADNI1)	ADNI2	83.33% (ADNI2)	–	–	–	89.89% (ADNI2)
Gao et al. [184]	2023	AD Classification & MCI Prediction	Multi	T1w-sMRI, Clinical Data	Intermediate (Single-Level)	ADNI1, ADNI2	CNN-Transformer	Serial	AD vs. NC	ADNI1 (with augmentation)	ADNI2	90.5%	84.6%	95.0%	–	93.9%
									pMCI vs. sMCI			73.6%	59.1%	82.1%	–	73.7%
Zhang et al. [62]	2024	AD Classification & MCI Prediction	Multi	Clinical data, T1w-MRI, AV45-PET	Intermediate (Hierarchical)	ADNI, OASIS3, NACC, C-PAS	(CNNs + Image-Tabular Cross-Transformer)	Hierarchical & (Cross Attention)	CN vs. MCI vs. DE (dementia) (COG)	5-fold CV on merged (ADNI /OASIS3 /NACC)	C-PAS	77.9% (Internal) 66.1% (C-PAS)	76.3% (Internal) 65.3% (C-PAS)	88.1% (Internal) 79.3% (C-PAS)	–	90.9% (Internal) 80.3% (C-PAS)
									sMCI vs. pMCI (MCIC)	5-fold CV on merged (ADNI/NACC)	–	76.1%	69.0%	79.3%	–	81.7%
Liu et al. [192]	2024	AD Classification	Multi	T1w-sMRI, SNP (Genetic data), Clinical data	Hybrid (Generative)	ADNI, UK Biobank, 441 SNPs	STAA	Serial	AD vs. NC	5-fold CV	–	87.2% (MRI) 69.7% (SNP)	82.3% (MRI) 62.5% (SNP)	91.1% (MRI) 75.4% (SNP)	–	92.0% (MRI) 71.1% (SNP)
Yu et al. [49]	2024	AD Classification & MCI Prediction	Multi	sMRI, Clinical data, Genetic data	Intermediate (Single-Level)	ADNI-1/2/GO)	AD-Transformer	Serial	AD vs NC	5-fold CV	–	95.9%	95.6%	96.1%	–	99.3%
									pMCI vs sMCI			75.3%	74.5%	75.5%	–	84.5%
Kadri et al. [178]	2022	AD Classification	Multi	sMRI, PET (FDG-PET)	Input-Level	ADNI	EfficientNet V2 + ViT	Serial	AD vs. MCI vs. CN	–	–	96.0%	–	–	–	–
Gao et al. [172]	2023	AD Classification & MCI Prediction	Multi	T1w-sMRI, T2w-sMRI, PET	Hybrid (Generative)	ADNI1, ADNI2, OASIS3	MLG-GAN + Mul-T	Serial	AD vs. CN	ADNI1	ADNI2	94.4% (ADNI2)	93.0% (ADNI2)	95.5% (ADNI2)	–	97.6% (ADNI2)
									pMCI vs. sMCI	ADNI1	ADNI2	77.8% (ADNI2)	75.4% (ADNI2)	79.6% (ADNI2)	–	82.8% (ADNI2)
Tang et al. [156]	2023	AD Classification	Multi	sMRI, PET (FDG-PET)	Intermediate (Attention-Based)	ADNI	CsAGP	Serial & (Cross Attention)	AD vs. CN	60%–20%–20% (T-V-T)	–	99.04%	97.96%	99.54%	–	99.80%
Odusami et al. [148]	2023	AD Classification	Multi	sMRI, PET (FDG-PET)	Input-Level	ADNI	VGG16 + ViT	Serial	AD vs. EMCI	5-fold CV	–	81.25% (MRI) 93.75% (PET)	–	–	–	–
Odusami et al. [149]	2023	AD Classification	Multi	sMRI, FDG-PET	Input-Level	ADNI, OASIS, AANLIB	Mobile ViT (MViTv3)	Serial	AD vs CN	–	(ADNI, OASIS, AANLIB)	99.00% (AANLIB) 96.00% (ADNI)	98.44% (AANLIB) 94.12% (ADNI)	99.00% (AANLIB) 97.00% (ADNI)	–	–
Odusami et al. [151]	2024	AD Classification	Multi	sMRI, FDG-PET	Input-Level	ADNI1, OASIS	Image Fusion (GLP + SPCNN) + (MViT) + (QDO)	Serial	AD vs. CN	60%–40% (T-V)	OASIS	94.73% (ADNI MRI) 80.00% (OASIS MRI)	90.70% (ADNI MRI) 90.00% (OASIS MRI)	100.00% (ADNI MRI) 70.00% (OASIS MRI)	–	–
Odusami et al. [150]	2024	AD Classification	Multi	sMRI, FDG-PET	Input-Level	ADNI, OASIS, AANLIB	MViTv3	Serial	AD vs CN	70%–20%–10% (T-V-T)	–	99.25% (ADNI) 99.50% (AANLIB) 96.00% (OASIS)	–	–	–	–
Khan et al. [182]	2024	AD Classification	Multi	sMRI, PET (FDG-PET)	Intermediate (Hierarchical)	ADNI	Dual-3DM^3^-AD	Hierarchical	AD vs. CN	10-fold CV	–	98.3%	97.4%	97.8%	–	–
Miao et al. [147]	2024	AD Classification	Multi	sMRI, PET (FDG-PET)	Intermediate (Hierarchical)	ADNI	MMTFN	Hierarchical	AD vs. NC	5-fold CV	–	94.61%	92.92%	–	93.89%	99.30%
Tang et al. [181]	2024	AD Classification	Multi	sMRI, PET (FDG-PET)	Intermediate (Hierarchical)	ADNI	Transformer + 3DCNN	Serial	AD vs. CN	10-fold CV	–	98.10%	95.82%	96.75%	99.09%	98.35%
Qiu et al. [193]	2024	AD Classification	Multi	sMRI (GM, WM), PET (FDG-PET)	Hybrid (Systemic)	ADNI-1/2/3, AIBL	MDL-Net	Hierarchical	AD vs. CN	10-fold CV	AIBL	96.37% (ADNI) 90.91% (AIBL)	97.40% (ADNI) 92.05% (AIBL)	95.38% (ADNI) 87.88% (AIBL)	–	98.48% (ADNI) 96.13% (AIBL)
Liu et al. [154]	2024	AD Classification	Multi	MRI (GM, WM, CSF), PET	Hybrid (Systemic)	ADNI	HAMMF	Serial & (Channel & Spatial Attention)	AD vs. NC	5-fold CV	–	93.15%	93.15%	–	93.57%	93.15%
Zhang et al. [155]	2023	AD Classification	Multi	sMRI, PET (FDG-PET), CSF	Intermediate (Attention-Based)	ADNI	MCAD	Serial & (Cross Attention)	AD vs. CN	5-fold CV	–	91.07%	91.03%	91.07%	–	94.07%
Kadri et al. [169]	2023	AD Classification	Multi	MRI, PET, CT	Intermediate (Single-Level)	OASIS-1/3, ADNI	Swin Transformer + Enhanced EfficientNetB0	Parallel	ND vs. VMD vs. MD vs. MoD	–	–	93.23% (OASIS MRI)	93.86% (OASIS MRI)	–	93.52% (OASIS MRI)	–
							Modified CoAtNet	Serial	ND vs. VMD vs. MD vs. MoD			97.33% (OASIS MRI)	97.34% (OASIS MRI)	–	97.35% (OASIS MRI))	–
Kadri et al. [188]	2025	AD Classification	Multi	MRI, PET, DTI, sfMRI	Hybrid (Multi-Level System)	ADNI, OASIS	Transformer Hybrid Model	Serial	AD vs. MCI vs. CN	–	–	99.98% (ADNI) 99.91% (OASIS)	–	–	–	–
Zuo et al. [177]	2023	AD Classification	Multi	DTI, rs-fMRI	Hybrid (Generative)	ADNI	BSFL	Serial & (Cross Attention)	LMCI vs. NC	10-fold CV	–	94.30%	93.42%	95.12%	–	98.12%
Zuo et al. [194]	2023	AD Classification	Multi	fMRI, DTI	Hybrid (Generative)	ADNI	CT-GAN	Serial & (Cross Attention)	LMCI vs. AD (Brainnetcnn)	5-fold CV		95.19%	95.24%	95.12%	–	94.27%

## Frameworks and reproducibility of algorithms

**Fig. 42:**
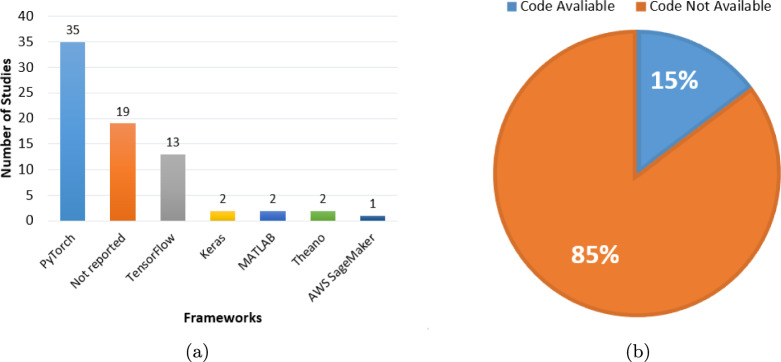
**a** The bar chart displays the frameworks used in the review studies.** b** The pie chart displays the percentage of code available and code not available in the review studies

An in-depth analysis of the frameworks employed in state-of-the-art studies involving ViTs and CViTs reveals a strong preference for a few dominant platforms, with PyTorch and TensorFlow leading the field, as illustrated in Fig. 42a.

- *PyTorch* was utilized in 35 of the reviewed studies, making it the most widely adopted framework in this domain [213]. Its popularity is largely attributed to its dynamic computation graph (a "define-by-run" approach), which offers greater flexibility for research, rapid prototyping, and intuitive debugging compared to the static graphs of earlier frameworks [112]. This flexibility, combined with strong community support and seamless GPU integration, makes it particularly effective for the complex, often bespoke architectures found in transformer-based medical imaging research.
- *TensorFlow* was employed in 13 studies [214]. It continues to be favored in production-scale environments due to its static computation graph, which allows for extensive optimization before execution, and its robust ecosystem for deployment, including tools like TensorFlow Serving and TensorFlow Extended (TFX). Its integration with TensorBoard for visualization also remains a key advantage for monitoring complex training runs.
- *Keras*, used in two studies, serves as a high-level neural networks API that simplifies the process of building and training models [215]. While it can use multiple backends, it is most commonly associated with TensorFlow, providing a more user-friendly interface for its powerful capabilities.
- *Other frameworks*, such as **Theano** [216], MATLAB’s Deep Learning Toolbox, and cloud-based platforms like **AWS SageMaker**, appeared less frequently. Their use often reflects specific institutional expertise or the need for particular engineering or cloud-computing functionalities.

Despite these technological advancements, a critical analysis reveals a significant barrier to scientific progress: a widespread lack of reproducibility. As detailed in Table 6, only ten of the 68 reviewed studies provided publicly accessible source code, with one requiring direct author correspondence for access. This finding, visualized in Fig. 42b, underscores a pervasive lack of transparency that not only hinders the verification and extension of prior work but also exacerbates the risk of methodological bias, such as data leakage, an issue well-documented in the AD classification literature [51]. To move forward, it is essential to first understand the tangible consequences of this reproducibility gap, establish clearer definitions of what reproducibility entails, and then define a set of minimum standards to facilitate it.

### The open-source ecosystem: gaps in contributions, model availability, and licensing

Beyond the general lack of reproducibility, a deeper analysis reveals critical gaps in the open-source ecosystem surrounding AD research that impede collaborative progress, scientific verification, and clinical translation. These deficiencies extend beyond the simple availability of code to include the scarcity of domain-specific pre-trained models and a widespread disregard for the legal and practical implications of software licensing.

***A pervasive lack of open-source contributions***

A central and alarming finding of this systematic review is the pervasive failure of researchers to share their source code. As our analysis in Table 6 demonstrates, a mere 15% of the 68 reviewed studies provided publicly accessible code. This finding is not an anomaly but reflects a systemic issue within the broader medical AI field. For instance, a recent meta-research study focusing on the radiology literature found that only a dismal 11% of DL models shared their code, creating a significant barrier to scientific verification and extension [217]. This practice forces independent research groups into a wasteful process of re-implementation based on incomplete methods, hindering the cumulative advancement of the field [218]. In response, leading journals like PLOS Digital Health have instituted robust policies emphasizing that authors must make all code publicly available and provide persistent identifiers, signaling a necessary shift in publication standards to foster a more transparent and collaborative research environment [219].

***The scarcity of domain-specific pre-trained models***

The majority of the transformer-based studies reviewed rely on a transfer learning paradigm, fine-tuning models that were pre-trained on massive, general-domain image datasets like ImageNet. However, recent research increasingly highlights a significant performance gap when these general-purpose foundation models are applied directly to specialized medical imaging tasks [220]. This underscores the critical need for models pre-trained on large-scale, curated medical data. Yet, the creation and sharing of such domain-specific foundational models remain a major challenge. Community-driven initiatives like "medigan," a library of pre-trained generative models for medical imaging, represent a crucial step forward but simultaneously highlight how rare and fragmented these resources currently are [221]. Unlike in Natural Language Processing, where repositories like Hugging Face have democratized access to thousands of pre-trained models, a centralized, easily accessible hub for pre-trained neuroimaging models is conspicuously absent. This scarcity forces individual labs to repeatedly incur the significant computational costs of pre-training, stifling innovation and creating a high barrier to entry for researchers at less-resourced institutions.

***The unaddressed issue of software licensing***

Even in the rare instances where source code is made available, the legal framework governing its use–the software license–is often an afterthought. This oversight carries profound implications for the scientific and commercial impact of a research artifact. The choice of license dictates whether a model can be legally and practically reused, modified, or integrated into a clinical product [222]. For example, the ad hoc reuse of code with restrictive or "copyleft" licenses, such as the GNU General Public License (GPL), can introduce significant legal risks and inadvertently prevent a promising research model from ever being commercialized [223]. In contrast, permissive licenses (e.g., MIT, Apache 2.0) are designed to encourage the widest possible adoption. Indeed, empirical studies have shown that the choice of a permissive license significantly increases the likelihood of software reuse compared to more restrictive options [224]. For the field to move towards clinically impactful tools that can be validated and deployed at scale, a cultural shift is needed where researchers not only share their code but do so under clear, permissive licenses that maximize its potential for both academic collaboration and translational development.

### The consequences of the reproducibility gap in AD research

The failure to ensure reproducibility is not a minor academic issue; it has tangible and detrimental consequences for the field. Firstly, it leads to a significant waste of resources, as independent research groups expend time and funding attempting to re-implement methods from papers that lack sufficient detail [218]. Secondly, it erodes scientific trust. When findings cannot be independently verified, it becomes difficult to distinguish between robust, generalizable models and those whose performance may be an artifact of a specific, undisclosed experimental setup. Most critically, this lack of transparency is a major impediment to clinical translation. For a diagnostic AI tool to be adopted in clinical practice, it must be thoroughly vetted, validated, and understood—a process that is impossible without full access to the underlying methodology.

### Defining levels of reproducibility in computational neuroscience

To address this challenge, it is essential to first establish a clear understanding of what reproducibility entails in the context of complex DL models [225]. In medical imaging research, two levels are particularly relevant:

- *Exact reproduction:* This refers to obtaining bit-for-bit identical results. However, given the inherent stochasticity in DL (e.g., random weight initialization) and variability in hardware and software environments (e.g., GPU architectures, CUDA/cuDNN versions), this standard is rarely achievable and is often not the primary scientific goal [226–228].
- *Close reproduction (Replicability):* A more practical and meaningful standard is close reproduction, or replicability. This involves achieving consistent trends and statistically similar performance metrics (e.g., accuracy within a small margin of error). This level of reproduction validates the core scientific claim of a method, demonstrating its robustness. However, as our analysis of the reviewed literature shows, the pervasive lack of critical implementation details makes even this more lenient standard profoundly challenging to meet [229–231].

### Pathways to enhanced replicability: from standards to systems

Overcoming the reproducibility crisis requires a multi-faceted approach, combining rigorous reporting standards for individual researchers with the development of community-wide infrastructure.

#### Minimum reporting standards for individual studies

We propose that the following practices, rooted in open science principles, be adopted as minimum standards for publication in the field [232]:

- *Public code and model weights:* The complete, commented source code for all stages of the experiment, along with the final trained model weights, should be deposited in a public repository. This is the bedrock of verification [233, 234].
- *Containerized computational environment:* The entire software stack—including OS, framework versions, and all library dependencies—should be encapsulated in a containerization script (e.g., a Dockerfile). This mitigates one of the largest sources of variability between research environments [235, 236].
- *Explicit hyperparameters and seeds:* All random seeds must be fixed and reported. A comprehensive list of all hyperparameters must be provided, as transformer models are known to be highly sensitive to these settings [237, 238].
- *Detailed preprocessing pipeline:* Preprocessing is a critical source of variability in neuroimaging. The exact software (e.g., FreeSurfer, FSL, SPM), its version, and all parameters used for steps like skull-stripping, registration, and normalization must be explicitly documented and, ideally, scripted. Adherence to standards like the Brain Imaging Data Structure (BIDS) [239, 240] should be strongly encouraged.
- *Unambiguous data splitting:* The exact subject identifiers for training, validation, and test sets must be provided to prevent data leakage and ensure that future models can be benchmarked on an identical basis. This is particularly crucial to avoid hidden stratification, where splits might inadvertently be biased by factors like scanner site or acquisition date [241, 242].

#### Systemic solutions for the broader community

While individual rigor is essential, lasting change requires systemic solutions. The community should work towards:

- *Development of open benchmark platforms:* The field would benefit immensely from standardized, open-source benchmark platforms, such as *Clinica* [243]. These platforms provide fixed data splits, standardized preprocessing pipelines, and common evaluation scripts, allowing for fair, unbiased, and direct comparison of novel algorithms.
- *Cultural shift encouraged by journals and funders:* Journals, conferences, and funding agencies play a crucial role in shaping scientific practice. By requiring code and data availability as a condition of publication or funding, and by encouraging the publication of high-quality replication studies, these institutions can create powerful incentives that align with the goals of transparency and reproducibility [244].

Adopting these combined individual and systemic approaches is fundamental to building a cumulative, trustworthy, and clinically impactful body of scientific knowledge in AD research.

**Table 6:** The framework used in reviewed studies and the open availability of the code repositories and metrics used in AD

Study	Framework	Code availability	Accuracy	Sensitivity recall	Specificity	Precision	F1-Score	AUC
[161]	PyTorch	Code	$\checkmark$	$\checkmark$	$\checkmark$			$\checkmark$
[158]	PyTorch	No	$\checkmark$					$\checkmark$
[134]	TensorFlow	No	$\checkmark$	$\checkmark$	$\checkmark$	$\checkmark$	$\checkmark$	
[207]	PyTorch	No	$\checkmark$	$\checkmark$	$\checkmark$			
[195]	TensorFlow	No	$\checkmark$	$\checkmark$		$\checkmark$	$\checkmark$	
[197]	Not reported	No	$\checkmark$	$\checkmark$	$\checkmark$		$\checkmark$	
[196]	Not reported	No		$\checkmark$		$\checkmark$	$\checkmark$	
[198]	TensorFlow	Code	$\checkmark$	$\checkmark$		$\checkmark$	$\checkmark$	$\checkmark$
[199]	PyTorch	Code	$\checkmark$	$\checkmark$	$\checkmark$	$\checkmark$		
[159]	PyTorch	No	$\checkmark$	$\checkmark$		$\checkmark$		
[200]	TensorFlow	No	$\checkmark$	$\checkmark$	$\checkmark$	$\checkmark$	$\checkmark$	
[201]	Not reported	No	$\checkmark$	$\checkmark$	$\checkmark$		$\checkmark$	
[202]	PyTorch	No	$\checkmark$	$\checkmark$	$\checkmark$	$\checkmark$	$\checkmark$	
[162]	Not reported	No	$\checkmark$	$\checkmark$		$\checkmark$	$\checkmark$	
[166]	PyTorch	No	$\checkmark$	$\checkmark$	$\checkmark$	$\checkmark$	$\checkmark$	$\checkmark$
[203]	Not reported	No	$\checkmark$					
[204]	AWS SageMaker	No	$\checkmark$	$\checkmark$		$\checkmark$	$\checkmark$	
[205]	Not reported	No	$\checkmark$	$\checkmark$	$\checkmark$			$\checkmark$
[206]	PyTorch	No	$\checkmark$	$\checkmark$	$\checkmark$	$\checkmark$	$\checkmark$	$\checkmark$
[163]	TensorFlow	No	$\checkmark$				$\checkmark$	$\checkmark$
[191]	TensorFlow	No	$\checkmark$					
[109]	PyTorch	Code	$\checkmark$	$\checkmark$	$\checkmark$		$\checkmark$	$\checkmark$
[153]	PyTorch	No	$\checkmark$					
[160]	Not reported	No	$\checkmark$	$\checkmark$	$\checkmark$	$\checkmark$		
[209]	PyTorch	No	$\checkmark$	$\checkmark$	$\checkmark$	$\checkmark$		
[81]	Not reported	No	$\checkmark$	$\checkmark$	$\checkmark$			$\checkmark$
[131]	Not reported	No	$\checkmark$	$\checkmark$	$\checkmark$			$\checkmark$
[31]	PyTorch	No	$\checkmark$	$\checkmark$	$\checkmark$			$\checkmark$
[32]	PyTorch	No	$\checkmark$	$\checkmark$	$\checkmark$			$\checkmark$
[145]	TensorFlow	No	$\checkmark$	$\checkmark$	$\checkmark$			$\checkmark$
[30]	TensorFlow	No	$\checkmark$	$\checkmark$	$\checkmark$	$\checkmark$	$\checkmark$	
[144]	Not reported	No	$\checkmark$	$\checkmark$	$\checkmark$		$\checkmark$	
[157]	Not reported	No	$\checkmark$	$\checkmark$		$\checkmark$	$\checkmark$	
[210]	Pytorch	No		$\checkmark$	$\checkmark$		$\checkmark$	$\checkmark$
[152]	Not reported	No	$\checkmark$	$\checkmark$		$\checkmark$	$\checkmark$	
[141]	Pytorch, TensorFlow, Keras, Theano	No	$\checkmark$	$\checkmark$	$\checkmark$	$\checkmark$	$\checkmark$	
[187]	Pytorch, TensorFlow, Keras, Theano	No	$\checkmark$	$\checkmark$	$\checkmark$	$\checkmark$	$\checkmark$	
[211]	Not reported	No	$\checkmark$	$\checkmark$		$\checkmark$	$\checkmark$	
[165]	Pytorch	No	$\checkmark$	$\checkmark$	$\checkmark$		$\checkmark$	$\checkmark$
[212]	TensorFlow	No	$\checkmark$	$\checkmark$	$\checkmark$			$\checkmark$
[140]	Not reported	No	$\checkmark$	$\checkmark$	$\checkmark$		$\checkmark$	$\checkmark$
[146]	PyTorch	Code	$\checkmark$	$\checkmark$	$\checkmark$			$\checkmark$
[102]	PyTorch	No	$\checkmark$	$\checkmark$	$\checkmark$	$\checkmark$	$\checkmark$	
[142]	TensorFlow	No	$\checkmark$	$\checkmark$		$\checkmark$	$\checkmark$	
[183]	Not reported	No	$\checkmark$	$\checkmark$		$\checkmark$	$\checkmark$	
[143]	PyTorch	No	$\checkmark$	$\checkmark$	$\checkmark$		$\checkmark$	$\checkmark$
[50]	PyTorch	Code with permission	$\checkmark$	$\checkmark$	$\checkmark$			$\checkmark$
[184]	PyTorch	No	$\checkmark$	$\checkmark$	$\checkmark$			$\checkmark$
[62]	PyTorch	Code	$\checkmark$	$\checkmark$	$\checkmark$		$\checkmark$	$\checkmark$
[192]	PyTorch	Code	$\checkmark$	$\checkmark$	$\checkmark$			$\checkmark$
[49]	PyTorch	No	$\checkmark$	$\checkmark$	$\checkmark$			$\checkmark$
[178]	PyTorch	No	$\checkmark$					
[172]	PyTorch	No	$\checkmark$	$\checkmark$	$\checkmark$			$\checkmark$
[156]	PyTorch	Code	$\checkmark$	$\checkmark$	$\checkmark$			$\checkmark$
[148]	PyTorch	No	$\checkmark$					
[149]	PyTorch	No	$\checkmark$	$\checkmark$	$\checkmark$	$\checkmark$	$\checkmark$	$\checkmark$
[151]	PyTorch	No	$\checkmark$	$\checkmark$	$\checkmark$			
[150]	PyTorch	No	$\checkmark$					
[182]	MATLAB	No	$\checkmark$	$\checkmark$	$\checkmark$		$\checkmark$	$\checkmark$
[147]	MATLAB	No	$\checkmark$	$\checkmark$		$\checkmark$	$\checkmark$	$\checkmark$
[181]	Not reported	No	$\checkmark$	$\checkmark$	$\checkmark$	$\checkmark$	$\checkmark$	$\checkmark$
[193]	PyTorch	Code	$\checkmark$	$\checkmark$	$\checkmark$			$\checkmark$
[154]	PyTorch	No	$\checkmark$	$\checkmark$		$\checkmark$	$\checkmark$	$\checkmark$
[155]	PyTorch	No	$\checkmark$	$\checkmark$	$\checkmark$			$\checkmark$
[169]	Not reported	No	$\checkmark$	$\checkmark$		$\checkmark$		
[188]	Not reported	No	$\checkmark$		$\checkmark$	$\checkmark$		$\checkmark$
[177]	TensorFlow	No	$\checkmark$	$\checkmark$	$\checkmark$			$\checkmark$
[194]	Not reported	No	$\checkmark$	$\checkmark$	$\checkmark$			$\checkmark$

## Overview of Alzheimer’s disease datasets in reviewed literature

The advancement of DL models for AD research is intrinsically linked to the availability of large, well-curated, and publicly accessible datasets [52, 245]. These resources provide the essential raw material for training, validating, and benchmarking novel computational approaches. This review encompasses studies utilizing several key public datasets that have become cornerstones of the field. A summary of these resources, including their primary characteristics such as subject numbers, modalities, and longitudinal nature, alongside the specific studies in this review that employed them, is detailed in Table 7. Understanding these datasets is crucial for contextualizing the advancements in ML and DL for AD.

- The *Alzheimer’s disease neuroimaging initiative (ADNI)* [246] is a multi-phase, longitudinal project initiated in 2003. It provides comprehensive data, including MRI, PET, DTI, CSF biomarkers, genetics, and clinical/cognitive assessments from over 2,000 participants across various stages (CN, EMCI, LMCI, AD). ADNI’s phased approach (ADNI-1, -GO, -2, -3, and the ongoing ADNI-4) has focused on standardizing protocols [247], identifying early AD markers [248], and understanding disease progression, making it a cornerstone resource for developing and validating AI-driven diagnostic tools. Its longitudinal design is particularly valuable for tracking changes over time, contrasting with some other cross-sectional datasets [44].
- The *Open access series of imaging studies (OASIS)* [249] project offers neuroimaging and clinical data from over 3,000 individuals. It comprises several datasets: OASIS-1 (cross-sectional MRI [249]), OASIS-2 (longitudinal MRI focusing on aging and dementia [250]), OASIS-3 (longitudinal multimodal data including MRI, PET, and CT over 30 years [251]), and OASIS-4 (focused on memory disorders and dementia). OASIS provides rich data for studying AD across different age groups and cognitive states.
- The *Australian imaging, biomarkers, and lifestyle study of ageing (AIBL)* [252], established in 2006, is a large-scale longitudinal study of approximately 2,500 participants. It investigates the roles of neuroimaging (PET, MRI), biomarkers, genetics, and lifestyle factors in AD progression, aiming to improve early detection and prevention strategies.
- *Kaggle AD MRI datasets* [253, 254] refer to several publicly hosted collections of sMRI scans (e.g.,  6,400 or  8,000 images), typically categorized into four clinical stages (ND, VMD, MD, MoD). These preprocessed, cross-sectional datasets facilitate the development and benchmarking of DL models, particularly for AD classification, despite lacking longitudinal follow-up and detailed patient metadata.
- The *UK Biobank* [255] is a large-scale (over 500,000 participants) prospective, longitudinal resource containing extensive health, neuroimaging (structural/functional MRI, DWI, SWI), genetic, and lifestyle data [256]. Its comprehensive nature supports broad research into aging and AD, enabling exploration of risk factors and early biomarkers.
- The *National Alzheimer’s coordinating center (NACC)* dataset [257] aggregates longitudinal clinical, genetic, and neuroimaging data from over 47,000 participants enrolled at U.S. Alzheimer’s disease Research Centers (ADRCs). It provides extensive, standardized information for studying AD progression and associated factors through its Uniform Data Set (UDS).
- The *HUASHAN-MCI dataset* [146], from Huashan Hospital, offers curated T1-weighted sMRI and clinical data for individuals with MCI and CN controls, specifically supporting research into early AD identification and MCI progression within a specific population.
- The *Whole brain atlas (AANLIB) dataset* [258], a Harvard Medical School resource, provides high-resolution MRI, CT, and PET scans of healthy individuals and patients with various neurological conditions, including AD. It serves educational and research purposes, particularly for cross-sectional comparisons of neuroanatomical variation.
- The *Dong-A university hospital cohort*, as described in the work of Shin et al. [162], provides longitudinal clinical, sMRI, and PET data from a Korean population, contributing to biomarker discovery and model development in a non-Western cohort.

Among these, ADNI’s longitudinal design, comprehensive multimodal data, defined collection protocols, and sustained participant involvement have made it a highly utilized and reliable resource in the reviewed AD research, particularly for studies focusing on disease progression [44].

### A critical analysis of dataset bias across the reviewed literature

A fundamental challenge that pervades the findings of this systematic review is the risk of inherent dataset bias, which calls into question the real-world generalizability of the high performance metrics reported. The studies analyzed, while architecturally innovative, are predominantly trained and validated on a small handful of research-centric datasets. This practice creates a substantial risk that the models are learning spurious correlations tied to the specific demographic and technical artifacts of the data, rather than robust, generalizable biomarkers of AD. We can categorize the 68 reviewed ViT and CViT studies into three groups based on their methodological approach to data handling and their resulting susceptibility to bias.

***Category 1: high risk of bias - single-dataset, internally validated studies***

The vast majority of the papers analyzed in this survey fall into this high-risk category. These studies utilize a single dataset–overwhelmingly the ADNI cohort–and report performance based solely on internal validation methods such as k-fold cross-validation or a simple train-test split. This methodology is critically vulnerable to both demographic and technical bias.

- *Demographic bias:* As established in critical reviews [259, 260], the ADNI cohort is not representative of the general population, being predominantly White and highly educated. Models trained exclusively on this data, such as the single-modality ViT from Alp et al. [134] and the efficient CViT from Khatri et al. [30], learn decision boundaries that are optimized for this specific demographic. While achieving high accuracy internally (often >95%), these models carry a high risk of underperforming on minority populations, potentially exacerbating healthcare inequities [261]. The numerous studies leveraging the 4-class Kaggle dataset [196, 197, 200] face a similar risk, as the demographic and acquisition details of this dataset are often poorly documented, making it impossible to assess its representativeness.
- *Technical bias:* When a model is trained and tested on data from the same multi-center study (like ADNI) without explicit harmonization, it may learn to identify scanner-specific artifacts as features. This "shortcut learning" [262] leads to inflated performance. Studies that report near-perfect accuracy on an ADNI-only test set, like Joy et al. [195] and Chen et al. [142], must be interpreted with extreme caution, as their models may have learned to recognize the specific "fingerprint" of ADNI’s imaging protocols rather than generalizable pathology.

***Category 2: moderate risk of bias - multi-dataset pooling without external validation***

A smaller group of studies attempts to increase data volume by pooling multiple datasets for training, such as the work by Mora-Rubio et al. [161] which combined ADNI and OASIS. While this approach can potentially improve model robustness, it significantly amplifies the risk of technical bias if rigorous data harmonization is not performed and reported. Without harmonization, the model can easily learn to differentiate between the datasets based on scanner-specific features (e.g., image contrast, resolution, noise profiles) and assign different decision rules to each, a form of shortcut learning. Because these studies still rely on an internal validation split from the pooled data, their reported performance may reflect this dataset-identification capability rather than true diagnostic accuracy.

***Category 3: lower risk of bias - studies employing rigorous external validation***

A select but growing number of studies, representing the methodological gold standard, demonstrate a clear effort to mitigate bias by performing rigorous external validation. These studies explicitly train their model on one dataset (or set of datasets) and test its performance on a completely separate, unseen cohort. This approach forces the model to learn true, generalizable pathological features that transcend the biases of a single source.

- The CViT models from Xin et al. [31] and Gao et al. [172] exemplify this best practice by training on ADNI and then successfully validating on the AIBL or ADNI-2 datasets, respectively. Their more modest, but more believable, accuracy scores (in the 90-93% and 77% ranges, respectively) are likely a far more realistic measure of real-world performance than the near-perfect scores from internally validated studies.
- The series of multimodal fusion studies by Odusami et al. [149–151] consistently validate their methods across ADNI, OASIS, and AANLIB, providing stronger evidence of generalizability.
- Perhaps the most rigorous example is the work by Zhang et al. [62], which leverages a massive, multi-cohort dataset (ADNI, OASIS-3, NACC, C-PAS) for both training and external validation. By demonstrating consistent, albeit lower, performance across these diverse sources, this study provides a more trustworthy assessment of its model’s capabilities.

***Summary discussion from the perspective of bias***

In summary, while it is impossible to definitively label any single study as "biased" without re-running the experiments, we can conclude that the vast majority of the 68 papers reviewed in this survey exhibit a high methodological risk of bias. This is a direct consequence of the field’s over-reliance on the demographically and technically homogeneous ADNI dataset, coupled with a pervasive lack of rigorous external validation. The studies in Category 1 and 2, which constitute the bulk of the literature, likely report optimistically inflated performance metrics that would not hold up under real-world clinical deployment.

Conversely, the small but significant group of studies in Category 3 are not necessarily "unbiased," but they employ a far more robust methodology designed to actively mitigate the effects of bias. The findings from these externally validated studies should be considered the most trustworthy and representative of the true state-of-the-art. Therefore, a critical conclusion of this survey is that for the field to advance towards clinically meaningful and equitable AI, a fundamental shift in validation practices is required, moving away from internal benchmarking on ADNI and towards a mandatory standard of external validation on diverse, multi-center, and demographically representative cohorts.

**Table 7:** Sources of AD and dementia data

Dataset	Number of subjects	Modalities	Longitudinal	Studies	Link
ADNI [263]	$\sim$2750	MRI, PET, DTI, CSF, Genetic, Clinical data, Cognitive assessments	Yes	[30–32, 49, 50, 62, 81, 102, 109, 131, 131, 134, 140–158, 160–163, 165, 169, 172, 177, 178, 181–184, 187, 188, 191–195, 197, 198, 200–207, 209, 210, 212]	http://adni.loni.usc.edu
OASIS [264]	3000+	sMRI, PET, Cognitive assessments	Yes	[62, 81, 141, 146, 149–151, 153, 158, 161, 169, 172, 187, 188, 191, 196, 202, 210]	http://www.oasis-brains.org
AIBL [265]	$\sim$2500	MRI, PET, CSF, Genetic, Clinical and cognitive assessments, lifestyle	Yes	[31, 32, 50, 191, 193, 209]	https://aibl.csiro.au
The Kaggle AD [253]	6400	sMRI	No	[141, 159, 187, 197–200, 211]	https://www.kaggle.com/datasets/tourist55/alzheimers-dataset-4-class-of-images
The Kaggle AD [254]	8000	sMRI	No	[201]	https://www.kaggle.com/datasets/arjunbasandrai/medical-scan-classification-dataset
UK Biobank [266]	500000+	sMRI, DWI, SWI, genomic, proteomic, lifestyle, and clinical assessments	Yes	[192]	https://www.ukbiobank.ac.uk/
NACC [267]	47,000+	Neuropathology, Genetic, imaging data	Yes	[62]	https://www.niagads.org/datasets/nacc
HUASHAN-MCI	-	sMRI	-	[146]	-
AANLIB [258]	-	sMRI, CT, PET	No	[149, 150]	https://www.med.harvard.edu/aanlib/
The Dong-A University Hospital cohort	593+	sMRI, PET, CSF, Clinical and Cognitive data	Yes	[162]	-

## Large vision models in Alzheimer’s disease diagnosis

The advent of Large AI Models, particularly in the vision and language domains, has catalyzed a paradigm shift across medicine [268–270]. Generalist foundation models like Med-PaLM M [271], BiomedGPT [272], and GMAI-VL [273] have demonstrated remarkable capabilities in handling diverse multimodal data, performing tasks ranging from radiology report generation to complex diagnostic reasoning. These systems are characterized by their massive scale, extensive pretraining on web-scale or large curated datasets, and their ability to perform zero-shot or few-shot tasks. This progress has been enabled by sophisticated techniques for creating high-quality, large-scale training data, often by leveraging powerful models like GPT-4V to denoise and reformat existing medical corpora [273, 274], and advanced training strategies like reinforcement learning to align models with specific clinical objectives [275].

However, a critical disconnect emerges when comparing this broader landscape to the focused domain of AD. The models reviewed in this survey, while powerful, are often developed and evaluated in a more traditional supervised learning paradigm. This raises a critical question, which this review will explore: Are we truly harnessing the "largeness" of these models, or are we primarily using them as powerful feature extractors for a specific, fine-tuned task? This question is particularly salient in light of recent benchmark studies [276], which suggest that the performance benefits of simple domain-adaptive pretraining may be less significant than widely assumed when subjected to rigorous, statistically-sound evaluation.

This section aims to bridge this conceptual gap by providing a critical synthesis of how LVM principles are currently being operationalized within AD research and charting a course for future development. First, we dissect the methodologies in the reviewed literature to understand how they leverage large-scale pretraining. Second, we introduce a critical discussion to reconcile the ambitious progress in general medical AI with the more measured, application-specific findings in the AD domain. Finally, we propose a conceptual framework for a next-generation LVM designed for AD, envisioning a future where the full potential of these foundational models can be realized for more accurate, personalized, and predictive diagnostics.

### LVM methodologies in current AD research

Within the reviewed AD literature, the principles of LVMs are primarily operationalized through two main avenues, though often falling short of a true, generalist foundation model paradigm. The first, and most common, is **transfer learning**, where models pretrained on massive, general-domain image datasets are fine-tuned for AD tasks [81, 152, 157, 199, 202]. The second is domain-specific **self-supervised learning (SSL)**, where architectures are pretrained on substantial medical imaging collections to learn relevant features without extensive manual labeling, a technique successfully applied with methods like DINO [277] in studies such as [146, 166, 205]. The following subsections detail how these LVM-inspired methodologies are applied in both single-modality and multimodality contexts.

#### Single-modality LVM-related applications

This subsection examines studies where LVM principles are applied to a single data modality, most commonly sMRI or PET. These applications, while not always multimodal, represent the foundational use of large pretrained models in the AD domain. They serve as a crucial stepping stone, demonstrating the value of pretrained visual representations before moving to more complex data fusion challenges. The subsequent discussion highlights how different LVM archetypes, such as ViTs and Swin Transformers, are adapted for these unimodal tasks.

***ViTs with general pretraining:***

A common approach in the reviewed literature is the adaptation of standard ViT architectures that have been pretrained on large-scale, general-domain image datasets like ImageNet or JFT-300 M [28, 278]. In this context, the "LVM" is the pretrained ViT backbone itself. Its "largeness" stems not from its parameter count (which may be standard, e.g., ViT-Base) but from the immense scale and diversity of the data used for its initial self-supervised or supervised pretraining. This process endows the model with a powerful and generalizable visual feature hierarchy.

However, it is crucial to distinguish this from more advanced, truly multimodal Large Vision-Language Models (LVLMs)–like BiomedGPT [272], XrayGPT [279], or GMAI-VL [273]. Those models are explicitly architected to process and align both vision and language inputs, often through dedicated cross-attention mechanisms, and are trained on massive image-text corpora. The ViT applications in the AD literature reviewed here typically do not possess this inherent language capability. Instead, they function as powerful vision-only backbones that are subsequently fine-tuned for a specific classification or prediction task. Knowledge distillation is not commonly employed; rather, the process is a direct transfer learning application. This approach is evident across several of the reviewed studies. For instance, Lu et al. [202] employed a DeiT (Data-efficient image Transformer) backbone [135], a variant of ViT, within their RanCom-ViT framework for sMRI classification. In a similar vein, Alshayeji [199] explicitly fine-tuned a "google/vit-base-patch16-224-in21k" model, pretrained on ImageNet-21k, for multi-stage AD diagnosis from MRI. Further demonstrating this trend, the OViTAD model proposed by Sarraf et al. [204] for rs-fMRI or sMRI analysis is also built upon the foundational principles of a pretrained ViT architecture.

***Swin transformers with general pretraining:***

The Swin Transformer [280], with its hierarchical architecture and efficient shifted-window self-attention, represents another LVM archetype frequently utilized in the AD domain. Similar to ViT, its power as an LVM comes from its pretraining on large datasets, which allows it to serve as a robust feature extractor. The reviewed studies then adapt this LVM for AD diagnosis, often within a hybrid CViT framework.

Here, the Swin Transformer acts as the "vision" component, and it is not inherently a multimodal model that accepts text like modern BERT-based or GPT-based LVLMs. Instead, it provides a strong visual foundation upon which more complex systems can be built, leveraging its proven capabilities for hierarchical feature extraction. This approach is exemplified in several of the reviewed studies. For example, Huang et al. [209] used a Swin Transformer as the core processing unit in their RST model for sMRI-based AD classification. In a different but related configuration, Hu et al. [81] developed a serial CViT where a CNN front-end extracts features that are subsequently processed by a Swin Transformer encoder, effectively using the Swin backbone to model global context.

#### Multimodality LVM-related applications

The true strength of the LVM paradigm becomes more apparent in multimodal applications, where the transformer’s ability to process heterogeneous token sequences is leveraged. Many of the reviewed CViT studies fall into this category, using a transformer component as a central fusion hub. A selection of prominent LVM-related models are systematically compared in Table 8.

The models featured in Table 8 were deliberately curated to be representative rather than exhaustive. The selection aims to construct a clear narrative that contrasts the state-of-the-art in the broader medical AI field with the specific LVM-inspired applications reviewed in this survey for AD. The general medical LVMs, such as BiomedGPT and GMAI-VL, were chosen to establish the current frontier of possibility, showcasing massive-scale data curation, advanced training paradigms, and true multimodal capabilities. In contrast, the AD-specific models were selected from the 68 papers systematically reviewed in this work to be representative of the dominant architectural and fusion strategies currently employed in the AD domain. This curated comparison is designed to effectively highlight the conceptual and methodological gap that is central to the discussion in this section, focusing on models that best illustrate key trends in data utilization, architectural design, and fusion mechanisms.

The architectures for these multimodal applications are often complex, but a representative example of the underlying principles is the **AD-Transformer framework** by Yu et al. [49], as illustrated in Fig. 41. This model exemplifies the common strategy of using modality-specific encoders to create a unified sequence of tokens, which are then processed by a central transformer to learn cross-modal relationships for a final diagnostic prediction.

- **CViTs with transformer-based fusion:** These frameworks use a CNN for initial feature extraction from imaging and then a transformer to fuse these features with other data types.
  - The **AD-Transformer** by Yu et al. [49], shown in Fig. 41, is a prime example. It tokenizes sMRI, clinical, and genetic data and processes them in a unified transformer encoder, which learns the complex cross-modal relationships.
  - The **3MT** framework by Liu et al. [50] uses a series of Cascaded Modality Transformers to iteratively integrate sMRI and clinical data, demonstrating a sophisticated LVM-inspired fusion strategy.
  - The **MCAD** network by Zhang et al. [155] employs cross-attention—a hallmark of advanced LVLMs—to deeply integrate features from sMRI, PET, and CSF biomarkers.
- **Graph transformers for multimodal data:** **BIGFormer** by Zou et al. [109] represents a highly specialized LVM-inspired approach. It structures rs-fMRI and genetic (SNP) data into a graph and uses a Graph Transformer to model the interactions. The attention mechanism here operates on a relational data structure, showcasing the adaptability of the transformer core.

Collectively, these multimodal applications highlight a clear and important trend. As AD research progresses, models are evolving from using pretrained transformers as simple feature extractors towards designing complex, integrated systems where the transformer is the central engine for multimodal fusion. Nonetheless, this analysis reveals three critical takeaways. First, current AD research leverages LVM principles primarily through transfer learning, adapting powerful, general-purpose vision backbones like ViT and Swin for specific diagnostic tasks rather than building end-to-end medical foundation models. Second, the application of true, generative LVMs capable of zero-shot reasoning remains largely unexplored within the specific scope of the literature reviewed here. Finally, this points to a significant conceptual and practical gap between the generalist capabilities of large-scale medical AI and the specialized, fine-tuning-heavy approaches that are currently dominant in AD research, a gap that the following discussion will address.

**Table 8:** Systematic Comparison of Representative LVM-related Models for Medical and AD-Specific Diagnosis. This table contrasts general medical foundation models with AD-specific applications reviewed in this survey, focusing on architectural design, data fusion mechanisms, and training paradigms. **Acronyms:** DAPT (Domain-Adaptive Pretraining), SFT (Supervised Fine-Tuning), RL (Reinforcement Learning), RLCF (Reinforcement Learning from Clinical Feedback), TFS (Trained from Scratch), MLP-P (MLP Projector Layer), Concat-Enc (Token Concatenation + Unified Encoder), X-Attn (Cross-Attention)

Model	Core architecture	Model size (Params)	Input modalities	Fusion mechanism	Pretraining data / scale	Adaptation/Fine-tuning strategy
*Category A: General Medical Foundation Models (Illustrative Examples)*						
BiomedGPT [272]	Encoder-Decoder Transformer	182M	Vision (Multiple), Text	Inherent in Generative Framework	14+ Public Medical Datasets	Unified Pretraining + SFT
GMAI-VL [273]	LLaVA-style (ViT + LLM)	7B	Vision (Multiple), Text	MLP-P	GMAI-VL-5.5M (5.5M pairs)	3-Stage (Alignment + IT)
HuatuoGPT-Vision [274]	LLaVA-style (ViT + LLM)	34B	Vision (Multiple), Text	MLP-P	PubMedVision (1.3M VQA)	DAPT via "Unblinded" Pipeline
UMed-LVLM [275]	MedVInT-based (Encoder + LLM)	~7B	Vision (Multiple), Text	Alignment via RL Rewards	MAU Dataset (5.8k images)	2-Stage (SFT + RL)
*Category B: AD-Specific Applications (From This Survey)*						
ViT-TST [134]	Dual Transformer (ViT + TST)	Not Specified	sMRI (single-modality)	Sequential Modeling	General-domain (ImageNet)	Standard SFT on ADNI
AD-Transformer [49]	Hybrid CNN-Transformer	Not Specified	sMRI, Clinical, Genetic	Concat-Enc	TFS on ADNI (1651 subjects)	End-to-end Training
3MT [50]	Hybrid CNN-Transformer	Not Specified	sMRI, Clinical	Cascaded X-Attn	General-domain (Pretrained Backbones)	SFT on ADNI (816 subjects) with Modality Dropout
MCAD [155]	Hybrid CNN-Transformer	Not Specified	sMRI, PET, CSF	Dual-Stream X-Attn	TFS on ADNI (467 subjects)	End-to-end Training with Alignment Loss
BIGFormer [109]	Graph Transformer	Not Specified	rs-fMRI, Genetic (SNP)	Graph-based Attention	TFS on ADNI (708 subjects)	End-to-end Training

### Critical discussion and synthesis: bridging the methodological gap

The analysis of current LVM methodologies in AD reveals a crucial tension in the current state of AD research. On one hand, the field is rapidly adopting powerful transformer architectures, particularly hybrid CViTs, to achieve high classification accuracy on benchmark datasets like ADNI. On the other, the prevailing methodology often involves supervised fine-tuning on these relatively small, homogeneous datasets. This approach, while effective for specific tasks, may not fully harness the zero-shot, generalist potential demonstrated by large foundation models in the broader medical AI landscape [272, 273].

This observation aligns with a growing critical perspective in the wider AI community, which questions whether simple Domain-Adaptive Pretraining (DAPT) [281] is a universally effective strategy. Rigorous benchmark studies, such as the one conducted by Jeong et al. [276], suggest that when subjected to fair, statistically sound comparisons, the performance benefits of DAPT can be marginal. This raises a pivotal question for the future of AD research: if simply training on more medical data is not a guaranteed path to superior models, what alternative or complementary strategies should be pursued?

To answer this, it is instructive to look at pioneering work from the adjacent field of general medical AI, which offers several clues for a more sophisticated path forward:

1. *The importance of high-quality data pipelines:* Progress may depend less on the sheer volume of data and more on the quality of its preparation. For instance, the work on HuatuoGPT-Vision [274] demonstrates that using a powerful MLLM in an "unblinded" capacity to see both an image and its context produces a demonstrably superior training dataset compared to "blind" text-only reformatting methods used by earlier models like LLaVA-Med [282]. This highlights the critical role of innovating the data engineering pipeline itself.
2. *The power of advanced training objectives:* Future breakthroughs may come from moving beyond standard classification loss functions. The development of UMed-LVLM [275] shows that incorporating a specialized reinforcement learning objective–one that explicitly rewards a model for localizing abnormalities and focusing its internal attention on clinically relevant visual evidence–can unlock entirely new capabilities that supervised fine-tuning alone cannot.
3. *The nuanced role of domain specificity:* While broad DAPT may have limitations, highly targeted adaptation can be very effective. Language models like BioBERT [283] and ClinicalBERT [284], as well as more recent efforts like AD-BERT [285], have shown that adapting a language model to the specific linguistic "dialect" of biomedical literature or clinical notes significantly improves performance on downstream NLP tasks. This suggests that the value of domain adaptation is highest when the target domain is narrow and highly specialized.

Synthesizing these external insights with the trends observed in our own review leads to a more refined hypothesis for the future of AD diagnostics. Progress is unlikely to stem from a single source, but rather from a synergistic triad of advanced methodologies. The most impactful models of the future will likely be those that are (a) built upon a foundation of high-quality, meticulously curated multimodal data, (b) trained with sophisticated objectives that go beyond simple accuracy to enforce clinical reasoning and interpretability, such as Reinforcement Learning from Clinical Feedback (RLCF), and (c) architected for true, deep multimodal fusion. This marks a crucial shift from a purely data-centric paradigm to a more holistic, methodology-centric one.

### Conceptual proposal for an advanced LVM in future AD research

**Fig. 43:**
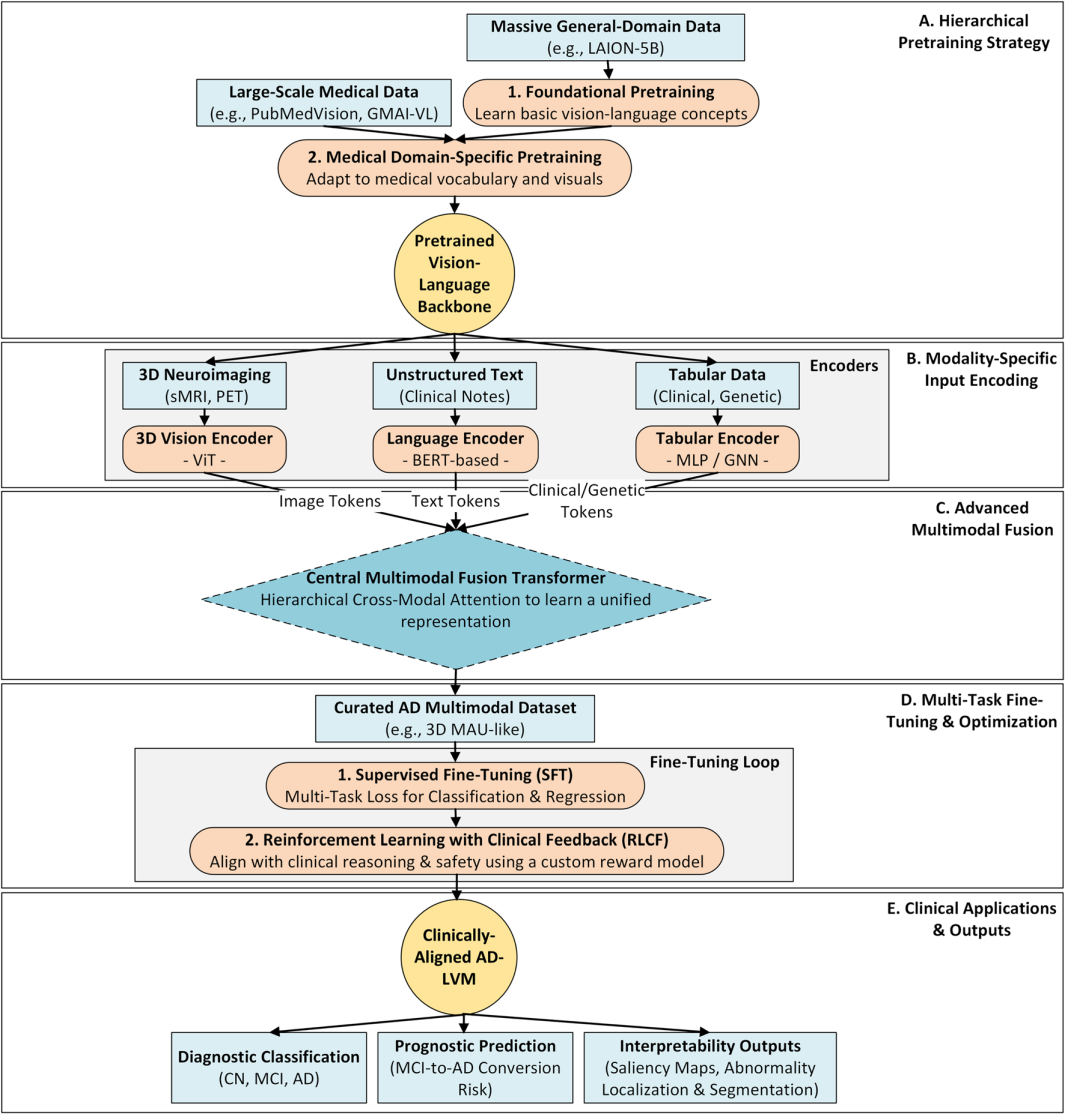
Conceptual architecture for a future-generation LVM designed for holistic AD assessment

Building on the insights from the current literature and the critical synthesis above, the next frontier in AD research lies in developing a foundational LVM specifically architected for the unique challenges of this disease. Such a model must move beyond simple classification to enable holistic, interpretable, and predictive assessment. Fig. 43 presents a conceptual architecture for such a system.

A conceptual LVM-based architecture for comprehensive AD assessment is proposed as follows:

- **Backbone architecture:** The foundation would be a state-of-the-art vision-language model, such as one adapted from architectures like PaLI [286] or Flamingo [287]. The choice of a vision-language backbone is critical, as it provides an inherent, powerful mechanism for integrating unstructured textual data (e.g., clinical notes, radiology reports) alongside visual information.
- **Hierarchical pretraining strategy:** A multi-stage pretraining approach is advocated:
  1. *General Foundational Pretraining:* Initialize with weights from models pretrained on massive, diverse general-domain image-text datasets (e.g., LAION-5B [288]) to capture fundamental visual concepts and language grounding.
  2. *Medical Domain-Specific Pretraining:* Conduct a secondary pretraining phase on a large, aggregated corpus of diverse medical data, such as the PubMedVision [274] or GMAI-VL-5.5M [273] datasets. This stage adapts the model to the specific vocabulary, syntax, and visual characteristics of the medical domain using techniques like contrastive image-text alignment.
- **Modality-specific input encoding and tokenization:** The LVM must effectively process heterogeneous data types native to AD research:
  - *Neuroimaging (3D sMRI, 3D PET):* Direct 3D volumetric processing is paramount. This can be achieved through 3D-native ViT encoders or factorized spatio-temporal attention mechanisms that process the volume as a sequence of 2D slices while explicitly modeling inter-slice dependencies, building on concepts from models like ViT-TST [134].
  - *Tabular and Textual Data:* Clinical scores, demographics, and genetic data (e.g., APOE status, Polygenic Risk Scores) would be projected into a shared embedding space to form "non-image" tokens, following established methods [49]. Unstructured clinical notes would be processed by the model’s inherent language encoder.
- **Advanced multimodal fusion architecture:** A central multimodal fusion transformer would learn a unified, synergistic representation:
  - *Joint Token Sequence Processing:* Token sequences from all modalities (3D imaging, tabular data, text) would be concatenated, augmented with modality-type and positional embeddings, and fed into the fusion encoder, similar to the AD-Transformer approach [49].
  - *Hierarchical Cross-Modal Attention:* The fusion encoder would feature layers of both self-attention (to refine representations within each modality’s token set) and cross-attention (where tokens from one modality serve as queries to attend to tokens from others). This facilitates deep, bidirectional information exchange, as demonstrated in architectures like MCAD [155].
- **Multi-task and reinforcement learning-based fine-tuning:** The final stage would involve fine-tuning the model on a curated, AD-specific dataset (like a 3D version of the MAU dataset [275]) using a combination of supervised and reinforcement learning:
  - *Supervised Fine-Tuning (SFT):* Train the model on multiple tasks simultaneously (e.g., diagnostic classification, prognostic prediction, abnormality segmentation) using a multi-task loss function, a technique shown to improve generalization [154].
  - *Reinforcement Learning with Clinical Feedback (RLCF):* Further refine the model using RL (e.g., PPO [289]) with a reward function designed by clinical experts. This reward would not only score diagnostic accuracy but also penalize clinically irrelevant hallucinations and reward the model for localizing its predictions to pathologically-relevant brain regions (similar to the Abnormal-Aware Rewarding in [275]), thereby ensuring the model’s reasoning aligns with clinical best practices.

This conceptual LVM aims to harness the vast representational power learned from broad pretraining and apply it through a sophisticated multimodal fusion and alignment architecture to unravel the complex, intertwined signatures of AD.

## Literature limitations and future research directions

The ascent of ViTs and particularly CViTs in AD research signifies a paradigm shift towards more sophisticated computational tools for diagnosis and prognosis. The 68 studies synthesized in this review underscore the considerable potential of these architectures. Nevertheless, a meticulous examination of the current research landscape reveals several persistent limitations and methodological gaps that extend from data handling to architectural strategy and the stark practicalities of clinical deployment. Addressing these is not merely an academic exercise but a critical prerequisite for transforming these promising research artifacts into the robust, generalizable, and clinically impactful assets required for integration into standard medical practice [290].

### Current limitations in transformer-based AD research

Despite the reported successes and high performance metrics on benchmark datasets, the current body of literature on transformer-based models for AD is characterized by several fundamental constraints that warrant careful consideration and a concerted effort to overcome.

#### Methodological and data integrity challenges

At the foundational level of data handling and experimental design, several inconsistencies and risks undermine the comparability and reliability of the reported findings.

- *Underutilization and ambiguity in multimodal fusion:* The clinical diagnosis of AD is inherently multimodal, a principle established by modern research frameworks that define the disease biologically through a combination of biomarkers [17]. Despite this, a significant portion of the reviewed works still focuses on single-modal inputs. As noted by the reviewer, there is a clear underutilization of multimodal fusion. When multimodal approaches are employed, they often default to simpler late-fusion strategies without fully exploring the potential of more deeply integrated early or intermediate fusion techniques. These advanced methods are better suited to model the complex, synergistic interactions between different biological processes, a key insight from foundational work in the field [46, 170]. Compounding this issue, the fusion methods are often vaguely described. Key details regarding data alignment, normalization across heterogeneous data types, and the precise mechanisms for feature integration are frequently omitted. This lack of methodological transparency creates a significant reproducibility risk in multimodal fusion, making it nearly impossible for other researchers to validate or build upon these complex architectures, a persistent challenge highlighted in prior systematic reviews [51].
- *Preprocessing inconsistencies:* The field lacks a standardized protocol for neuroimaging preprocessing. As highlighted by the reviewer, studies differ substantially in their choice of software and parameters for fundamental steps like skull-stripping, bias-field correction, and spatial normalization. This heterogeneity introduces significant variability into the final data, making fair and direct comparisons of model performance across studies exceptionally difficult, a widely acknowledged issue in the literature [44]. Indeed, this very problem of non-standardized preprocessing has motivated large-scale community efforts to develop robust, automated pipelines specifically designed to improve the reproducibility of neuroimaging research [291].

#### Architectural strategy and spatiotemporal modeling

- *Underutilization of spatiotemporal volumetric data:* A primary and pervasive limitation across the reviewed literature is the suboptimal handling of the rich, four-dimensional nature of neuroimaging data. This is particularly evident in studies employing pure ViT architectures. To manage computational complexity or adapt models originally designed for 2D images, many studies reduce inherently 3D volumetric data, such as sMRI or PET scans, into sequences of 2D slices [31, 134, 156, 158]. This dimensional reduction, while pragmatic, risks discarding crucial volumetric information and complex 3D spatial relationships, a known challenge in neuroimaging analysis [51]. This issue is significantly compounded in longitudinal research, where the temporal dimension—the critical fourth dimension representing disease progression over time—is often inadequately modeled. This underutilization of the temporal axis, addressed by only a few pioneering models [131, 292], represents a significant missed opportunity for moving beyond simple classification towards true, personalized prognosis.
- *Pragmatic adaptation over De novo design:* While ViTs offer promising results, they were not originally conceived for the specific constraints of medical imaging. As suggested by the reviewer, a more practical and efficient research trajectory may not lie in developing entirely new architectures from the ground up. Instead, a focus on the pragmatic adaptation of existing, well-validated ViT frameworks is warranted. This involves leveraging techniques like transfer learning to address the challenge of smaller neuroimaging datasets and strategically combining transformers with complementary models, such as using CNNs for their proven strength in local feature extraction within an ensemble or a modular hybrid system. This modular approach could improve both efficiency and clinical applicability while building on established methods.

#### Dataset constraints and generalizability challenges

- *Dataset homogeneity and bias:* The field’s progress is heavily reliant on a few well-established, publicly available datasets, with ADNI being the most prominent. This benchmark-centric approach can inadvertently lead to models that are overfitted to the specific demographic characteristics and imaging protocols of that cohort [51]. This raises significant concerns about model fairness and the propagation of data bias [260], as models may perform inequitably on underrepresented groups. Consequently, the high performance metrics reported do not guarantee real-world generalizability, and comprehensive external validation on truly independent, multi-center cohorts remains a significant gap [293].
- *The ’Accuracy Paradox’ and the Illusion of clinical readiness:* A recurring theme in the reviewed literature is the reporting of exceptionally high performance metrics, often accuracies exceeding 99% [195, 196]. While impressive within a research context, these figures must be interpreted with extreme caution, as they risk creating an illusion of clinical readiness. High accuracy on curated, often balanced, test sets does not translate to real-world clinical utility. A model that achieves 99% accuracy distinguishing clear-cut AD from healthy controls is of limited value if it fails on the diagnostically ambiguous and clinically critical task of predicting MCI conversion, where performance is often substantially lower. This disconnect underscores a critical need to shift evaluation beyond simple accuracy towards metrics that reflect genuine clinical utility, such as robust AUC scores on imbalanced classes, performance on external validation cohorts, and model calibration to ensure that predictions are reliable in practice [294].
- *Uncritical use of data augmentation:* While many studies employ data augmentation to expand limited datasets, this is often done without a critical evaluation of the techniques’ clinical validity. Simple geometric augmentations (e.g., random rotations, flips, zooms) may create biologically implausible neuroanatomical variations, leading to models that overfit to artifacts rather than learning true pathological features [295]. The impact of these techniques on model generalizability is rarely tested. Future research must move towards more sophisticated and validated augmentation strategies. This includes leveraging advanced generative models, such as Generative Adversarial Networks (GANs) [296] or diffusion models [297], to create synthetic data that is not only visually realistic but also pathologically consistent with the known course of AD, thereby providing a more robust and clinically relevant method for dataset expansion.

#### Computational demands and scalability issues

The core mechanism of transformer architectures, self-attention, comes with an inherent computational cost that scales quadratically with the length of the input sequence [27]. When applied to high-resolution three-dimensional neuroimaging data, the demand for GPU memory and processing time becomes a significant bottleneck [131, 147]. This computational burden is not just a research inconvenience; it is a direct barrier to real-world adoption. It poses a formidable obstacle to the practical deployment of these models in routine clinical workflows, which often operate within resource-constrained hospital IT environments and demand rapid diagnostic turnaround times.

#### Deficiencies in model trustworthiness and interpretability

- *Robustness and uncertainty quantification:* High accuracy on a clean, curated test set does not ensure model robustness against perturbations common in real-world clinical data, such as scanner artifacts or patient motion [298]. More critically, the vast majority of the reviewed studies do not report any form of uncertainty quantification. A model that provides a categorical prediction without a reliable measure of its own confidence is clinically untenable. For high-stakes medical decisions, such a deterministic "black box" is not just incomplete–it is potentially dangerous [299].
- *The interpretability gap and the failure to bridge the semantic divide:* A formidable barrier to widespread clinical adoption and physician trust remains the inherent opacity of DL models, often termed the "black-box" problem [300]. While several studies incorporate post-hoc XAI techniques such as saliency maps [197, 301], these methods offer, at best, a superficial glimpse into the model’s focus. They fundamentally fail to bridge the *semantic gap* between statistical correlation and clinical causation; they can show *where* a model is looking but cannot explain *why* in a way that aligns with established medical principles [302]. The more profound limitation is that these models operate in an evidence vacuum, functioning as ’knowledge islands’ completely disconnected from the vast repository of biomedical knowledge that underpins human clinical reasoning. The absence of integration with external knowledge bases, a capability now unlocked by Large Language Models (LLMs), prevents these models from articulating a diagnostic argument. Without this knowledge grounding, they cannot justify their outputs in a clinically coherent narrative, severely curtailing their trustworthiness and utility in collaborative diagnostic workflows [303].

#### Barriers to clinical adoption and deployment

Beyond algorithmic performance, the most significant hurdles are often operational, lying at the critical interface between the model and the existing clinical ecosystem. Successfully navigating this "last-mile problem" is essential for any AI tool to achieve real-world impact.

- *Integration and workflow challenges:* A functional model in a research environment is far from a deployable clinical product. There remains a vast engineering chasm in integrating these complex models into legacy hospital infrastructures, particularly with Picture Archiving and Communication Systems (PACS) and Electronic Health Record (EHR) systems [304]. This requires solving significant challenges in data security and interoperability, and crucially, designing systems that can present model outputs to clinicians in an intuitive, actionable, and non-disruptive format.
- *Model lifecycle and maintenance:* A static, "one-and-done" trained model is a research artifact, not a clinical tool. In a dynamic healthcare environment, its performance will inevitably degrade. The field must therefore develop robust strategies for the entire model lifecycle, including continual monitoring, retraining, and updating as new data becomes available or as clinical guidelines evolve. This necessitates a shift towards online or continual learning paradigms capable of adapting to a constantly changing clinical landscape [305].
- *Human-in-the-loop and feedback-based learning:* The future of clinical AI is not autonomous replacement but collaborative intelligence. There is a critical need for systems designed to augment, not replace, clinician expertise. Developing feedback-based learning mechanisms, where clinician inputs are used to refine and personalize the model over time, is an essential step toward building resilient, trustworthy systems that continuously improve and adapt through their interaction with expert users in a real-world setting [302].

#### Ethical AI and data privacy challenges

Beyond algorithmic performance, the application of advanced AI models to AD neuroimaging data raises profound ethical and data privacy challenges that are not fully addressed by the current literature. The patient population central to this research is uniquely vulnerable, often experiencing cognitive decline that can impact their capacity to give fully informed consent for data sharing and reuse [306]. This places an elevated responsibility on researchers to ensure that data governance and privacy protocols are exceptionally robust.

A significant concern is the risk of re-identification from high-dimensional neuroimaging data. While datasets are typically "anonymized" by stripping explicit personal identifiers, studies have shown that facial features can be reconstructed from sMRI scans, and demographic or even genetic information can be inferred from the data itself [307, 308]. Transformer models, with their ability to learn complex, subtle patterns, may inadvertently learn and encode this sensitive, identity-linking information. The potential for a privacy breach, where a patient’s identity and dementia status could be linked, represents a significant ethical risk that requires more advanced privacy-preserving techniques than simple anonymization.

Furthermore, the principles of trustworthy AI, including fairness and accountability, are paramount in this domain [259]. As discussed, models trained on demographically skewed datasets risk perpetuating algorithmic bias. The ethical implications of deploying a diagnostic tool that is less accurate for certain populations are severe. Addressing these challenges requires a shift towards a "privacy-by-design" and "ethics-by-design" approach, where considerations of consent, re-identification risk, and algorithmic fairness are integrated into the research lifecycle from the very beginning, rather than being treated as an afterthought.

### Future research directions

Addressing the identified limitations necessitates a multi-pronged research agenda aimed at enhancing the robustness, generalizability, interpretability, and ultimately, the clinical utility of transformer-based models for AD. This can be conceptualized as four interconnected research pathways.

#### Pathway 1: Embracing longitudinal and multimodal complexity

The future of AD diagnosis lies in moving beyond static, unimodal analysis to capture the full, dynamic complexity of the disease.

- *From volume to function and beyond:* Future research must expand its focus beyond sMRI-derived volumetric data. It is crucial to develop and validate models that can integrate functional data from fMRI and EEG [203, 205], which capture neural connectivity and activity, alongside molecular imaging from advanced PET tracers [162, 166] and microstructural information from DTI [177].
- *Deepening multimodal integration:* The next generation of models must be architected to seamlessly fuse an even wider array of data types. This includes incorporating structured data like genetic profiles (e.g., GWAS, PRS) [49, 192] and fluid biomarkers from CSF [155], as well as unstructured data such as video recordings of patient examinations to capture subtle motor and behavioral signs, and text from clinical notes and patient histories to provide rich contextual information.
- *Longitudinal modeling as a core priority:* There is a critical need for architectures explicitly designed to model disease trajectories over time. This involves integrating temporal attention mechanisms, RNNs, or State-Space Models within hybrid CViT frameworks. The goal is to effectively process longitudinal multimodal data and shift the research focus from static classification to dynamic, personalized prognosis, building on promising early work in this area [131, 292].

#### Pathway 2: Next-generation architectures for integration and efficiency

To overcome current limitations, a new wave of architectural innovation is required that focuses on integration, efficiency, and enhanced capabilities.

- *Lightweight and efficient transformers:* To be clinically viable, models must be deployable. Future research must prioritize the development of computationally efficient architectures that are powerful yet practical for standard hospital IT infrastructure. This involves the exploration and refinement of techniques like sparse attention, linear attention, model quantization, knowledge distillation, and token compression [31, 202].
- *Integrating LLMs and LVMs through modular frameworks:* While the integration of language and vision models shows immense promise, a monolithic architecture may hinder, rather than help, clinical practicality. As suggested by the reviewer, a more pragmatic future lies in developing modular frameworks. This approach offers a direct solution to many real-world deployment challenges: it preserves the interpretability of individual components, allows for flexible operation when certain data modalities are unavailable, and facilitates independent updates and validation. Such a design represents a tangible, pragmatic pathway to clinical integration, aligning powerful AI capabilities with the realities of the diagnostic workflow [309].

#### Pathway 3: From black boxes to integrated clinical partners via advanced XAI

A critical barrier to the real-world clinical integration of advanced AI is their "black-box" nature; thus, building robust explainability is a non-negotiable step toward translation. Future research in XAI must prioritize the development of an interactive and evidence-based explanatory ecosystem. Key research priorities should include:

- *Holistic multimodal explanations:* XAI techniques must evolve to explain the complex interplay between different data modalities. This requires moving beyond single-modality saliency maps to develop methods that can visualize and articulate how cross-modal interactions, as modeled in fusion architectures like MCAD [155] and 3MT [50], shape the model’s final prediction.
- *Clinician-centric interactivity:* To be useful in practice, XAI cannot be static. Future systems should be designed as interactive partners, allowing clinicians to probe a model’s reasoning through natural language questions or to explore "what-if" scenarios. This transforms the model into a dynamic tool for differential diagnosis, expanding on the foundational work seen in [163, 197].
- *Evidence-based and verifiable reasoning:* Leveraging Retrieval-Augmented Generation (RAG), future XAI systems must connect their internal findings to external, authoritative knowledge bases. By "citing its sources," a model can link its predictions not just to the patient’s imaging data but also to established clinical guidelines or landmark studies, providing the verifiable, evidence-based reasoning that is standard in medical practice.

#### Pathway 4: Building a robust and ethical research ecosystem

To ensure that progress is real, cumulative, and ethically sound, the research community must adopt more rigorous scientific and validation practices.

- *Establishing open benchmark platforms:* There is an urgent need for standardized, open-source benchmark platforms that allow for the fair and direct comparison of different models on common datasets with fixed preprocessing and evaluation protocols, thus mitigating issues of data leakage and inconsistent reporting [51].
- *Pioneering privacy-preserving AI through advanced federated learning:* To fundamentally overcome the limitations of dataset homogeneity and build models that generalize across diverse patient populations, pioneering privacy-preserving, distributed learning methodologies is not an option, but an imperative. Federated Learning (FL) stands as the leading paradigm to achieve this, enabling collaborative model training on multi-center datasets without centralizing sensitive patient data, thereby respecting critical regulatory frameworks like GDPR and HIPAA [310]. However, recommending FL as a simple, off-the-shelf solution for large-scale transformer models would be a gross oversimplification of the formidable engineering and algorithmic hurdles that currently impede its widespread clinical deployment. Therefore, a critical future research direction lies not just in applying FL, but in *innovating FL specifically for the demands of high-resolution medical imaging and large transformer architectures*. This research must aggressively tackle the key bottlenecks: developing novel gradient compression and model quantization techniques to mitigate communication overhead [311], investigating advanced aggregation algorithms robust to the statistical heterogeneity of clinical data, and exploring hybrid models like split learning that can alleviate the computational burden on individual institutions [312]. Successfully solving these challenges is essential to unlock the full potential of multi-center collaboration, paving the way for truly robust and equitably performing diagnostic AI.
- *Mandating fairness and bias audits:* All future studies proposing models for clinical application should include a systematic analysis of model performance across different demographic subgroups (e.g., by age, sex, ethnicity, and socioeconomic status). This proactive auditing, as called for by related reviews [57, 260], is essential to identify and mitigate biases, ensuring that these powerful tools are equitable and safe for all populations.

**Fig. 44:**
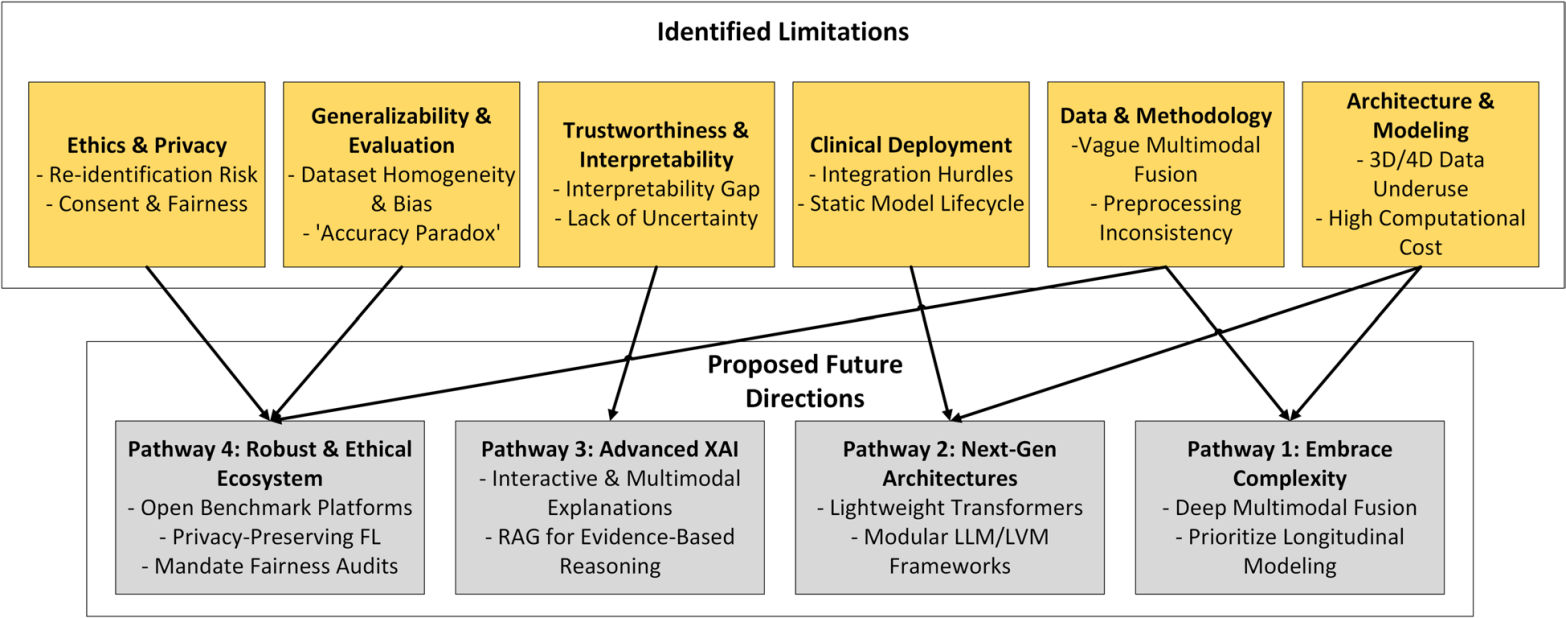
An overview of the strategic pathways for advancing transformer-based AI in AD research. This diagram outlines the key limitations identified in the current literature (above) and maps them to the corresponding future research directions (below) that are discussed in this section, providing a roadmap from current challenges to future solutions

In summary, the path from the promising research artifacts of today to the clinically integrated tools of tomorrow is contingent on addressing the critical limitations identified in this review. The strategic pathways outlined in our future research directions are designed to directly confront these challenges. This mapping from problem to solution is visually encapsulated in Fig. 44, which provides a schematic overview of the necessary steps toward achieving more sophisticated spatiotemporal modeling, deeper multimodal integration, and a foundational commitment to trustworthy, reproducible, and equitable AI.

## Conclusion

This systematic review has charted the rapid evolution and significant impact of transformer-based models on AD research. Through a rigorous, PRISMA-guided analysis of 68 studies, our work confirms a decisive architectural shift in the field. While ViTs established a new baseline for processing neuroimaging data, it is the hybrid CViT architectures that have emerged as the dominant and most promising paradigm, effectively synergizing the local feature extraction of CNNs with the global context modeling of transformers.

Our analysis of the literature reveals several critical trends and gaps. First, while single-modality data, predominantly sMRI, remains a foundational input, the most significant performance gains and the most promising path forward lie in the sophisticated integration of multimodal data. Frameworks that fuse neuroimaging with clinical, genetic, and biomarker data are better equipped to capture the multifaceted nature of AD. Second, a conspicuous research gap exists in prognostic modeling; the majority of studies focus on diagnostic classification, with the clinically vital task of predicting MCI-to-AD progression remaining relatively underexplored.

Furthermore, this review highlights persistent challenges that must be addressed to bridge the gap between high-performance modeling and clinical reality. These include the high computational demands of transformer models, the need for enhanced model interpretability beyond simple attention maps, and the risks of data homogeneity and bias inherent in the field’s reliance on a few key datasets. Furthermore, our review highlights a significant reproducibility crisis, with the vast majority of studies not providing public access to their code, a practice that must change to foster a transparent and collaborative research ecosystem.

Moving forward, the trajectory of this field will be defined by its ability to address these challenges head-on. Future research must prioritize the development of novel and robust multimodal fusion strategies, the engineering of lightweight and computationally efficient transformer architectures, and the deep integration of XAI and LVM principles. By focusing on these areas and committing to rigorous, multi-center validation, the research community can harness the full potential of transformer-based architectures. The ultimate goal is to move beyond academic benchmarks and deliver clinically deployable, trustworthy, and equitable AI solutions that will genuinely impact the early and accurate diagnosis of AD, thereby improving patient care and disease management worldwide. ViT and CViT architectures represent a significant step toward applying AI models in the clinical domain. However, as discussed in the Literature Limitations and Future Research Directions section, several critical challenges remain before these models can achieve successful clinical translation and real-world deployment.

## Data Availability

No datasets were generated or analysed during the current study.
